# Benchmarking organic active materials for aqueous redox flow batteries in terms of lifetime and cost

**DOI:** 10.1038/s41467-023-42450-9

**Published:** 2023-10-21

**Authors:** Dominik Emmel, Simon Kunz, Nick Blume, Yongchai Kwon, Thomas Turek, Christine Minke, Daniel Schröder

**Affiliations:** 1 Institute of Energy and Process Systems Engineering (InES), Technische Universität Braunschweig, Braunschweig, Germany; 2 Institute of Physical Chemistry, Justus-Liebig-University Giessen, Giessen, Germany; 3 Center for Materials Research, Justus-Liebig-University Giessen, Giessen, Germany; 4 Institute of Mineral and Waste Processing, Recycling and Circular Economy Systems, Clausthal University of Technology, Clausthal-Zellerfeld, Germany; 5 Research Center Energy Storage Technologies, Goslar, Germany; 6 Department of Chemical and Biomolecular Engineering, Seoul National University of Science and Technology, Nowon-gu, Seoul Republic of Korea; 7 Institute of Chemical and Electrochemical Process Engineering, Clausthal University of Technology, Clausthal Zellerfeld, Germany

**Keywords:** Energy science and technology, Materials for energy and catalysis, Energy storage, Batteries

## Abstract

Flow batteries are one option for future, low-cost stationary energy storage. We present a perspective overview of the potential cost of organic active materials for aqueous flow batteries based on a comprehensive mathematical model. The battery capital costs for 38 different organic active materials, as well as the state-of-the-art vanadium system are elucidated. We reveal that only a small number of organic molecules would result in costs close to the vanadium reference system. We identify the most promising candidate as the phenazine 3,3′-(phenazine-1,6-diylbis(azanediyl))dipropionic acid) [1,6-DPAP], suggesting costs even below that of the vanadium reference. Additional cost-saving potential can be expected by mass production of these active materials; major benefits lie in the reduced electrolyte costs as well as power costs, although plant maintenance is a major challenge when applying organic materials. Moreover, this work is designed to be expandable. The developed calculation tool (ReFlowLab) accompanying this publication is open for updates with new data.

## Abstract (web summary)

To guide research and implementation of aqueous organic redox flow batteries it is essential to estimate their potential costs. In this perspective, the authors present an overview of the potential cost of organic active materials for aqueous flow batteries and identify cost reduction routes.

## Introduction

With an increasing focus on renewable energy resources, the search for economic stationary energy storage systems is more important than ever^1,2^. One promising electrochemical storage technology is the redox flow battery (RFB) in which the charge carriers are stored in liquid electrolytes and pumped through an electrochemical cell referred to as flow cell. This open cell architecture allows to decouple the place of the electrochemical reaction from the place where the energy is stored; peak power and capacity can be scaled independently from each other^3^. Thus, RFBs are very versatile and can be applied in different ranges of applications^4^. Additionally, in the case where aqueous electrolytes are applied, safety concerns are low in comparison to other energy storage systems like the lithium-ion battery technology^3^.

The most advanced RFB technology is based on vanadium salt electrolytes. Assemblies of all-vanadium redox flow batteries (VRFB) are used in residential storage systems, as well as in large-scale energy storage systems for grid applications^4^. They show good long-time stability with a battery lifetime of up to 20 years^5^. One major disadvantage is the high acquisition cost for the needed electrolytes, as well as the used ion exchange membrane. Moreover, the high costs of vanadium salts are fluctuating because of their connection to industrial steel production^3^. To overcome this burden and to reduce the overall cost of a redox flow system, current research is focused on finding novel active materials^3,6,7^.

Organic active materials are very promising for replacing VRFBs in either aqueous or non-aqueous systems because they can be tailored for the specific application needs^8,9^. These organic compounds are based on, or can be synthesized from, abundant resources^3,10–12^. Furthermore, applying especially larger organic molecules has the potential of further cost reduction due to additional separator options. Thereby, the more expensive ion exchange membranes could be substituted by cheaper size-selective separators, which reduces one of the major cost factors^3,13^. By changing substituents or the base structure of molecules, the solubility or the number of electrons transferred in the redox process of active materials can be improved. Therefore, many tailored active materials and electrolytes, with various energy density, have been proposed in literature^8,9^. The main focus is thereby on the characterization of the active materials and the cycling stability over a few days, while studies on the long-term cycle stability of RFBs, as well as the economic point of view, are less considered^14^. This common practice can overestimate the benefit of the investigated materials without seeing the long-term goal, which is to realize an economic and cost-efficient RFB.

Despite being convincing in terms of their potential advantages, organic active materials for RFBs are still struggling with drawbacks. Organic molecules undergo multiple degradation reactions, which could have a significant impact on the overall battery performance. Currently, the long-term stability of organic active materials cannot compete with their inorganic counterparts^10^. Additionally, many molecules that are studied show low solubility in water-based electrolytes, in some cases not fully compensated by an increased number of transferred electrons, leading to insufficient energy density^1,3^.

Although finding novel organic active materials is still the focus of research, multiple start-up companies on organic RFBs have been founded recently, illustrating the increased relevance of this technology. Companies such as Kemiwatt^15^ (France), CMBlu^16^ (Germany), CERQ^17^ (Germany), or Quino^18^ Energy (USA) are promoting RFBs with organic active materials^19^.

Independent of the organic material being used, the commercial success of RFBs will be governed by the overall system cost. The cost calculation for RFB systems is not trivial, because many aspects such as the stack design, the balance-of-plant, or the maintenance costs, need to be considered. So far, Brushett et al.^14^ presented an economic model that separates the total cost of RFBs into three major parts: stack costs, electrolyte costs, and costs due to electrolyte exchange. In this work, we present a techno-economical (TE) model that extends the already proposed method in order to calculate the total costs of various active materials in an industrial-scaled RFB system. Instead of a general view of organic materials for RFBs, we compare active materials on the cell level. Our approach focuses on the individual properties that change when applying different active materials. As an additional extension, the required number of stacks is calculated by applying a polarization model taking the electrochemical kinetics and mass transfer effects into account. In this study, aqueous negolytes and posolytes of organic active materials are considered and compared to the VRFB. (Note that we use “posolyte” and “negolyte” electrolytes as is common in latest literature reviews instead of the traditional terms “catholyte” and “anolyte” to avoid confusion, since the latter is only valid for discharging^20^.)

The proposed RFB cost model has the potential to be updated continuously, as various parameters that are implemented (such as future active material prices or application-related electrolyte capacity fade rates) can change daily, or can be predicted more precisely in the future. With a comparison at cell level, including capacity and power-determining properties, as well as electrolyte degradation, our study gives the reader an assessment of the capability of the considered active materials. Furthermore, our calculation model can serve as benchmark for the buildup of viable organic RFB systems for large-scale energy storage. Finally, we give the reader an outline of the projected cost results for state-of-the-art active materials and identified the phenazine 1,6-DPAP (3,3’-(phenazine-1,6-diylbis(azanediyl))dipropionic acid) as the most promising one out of 38 studied molecules.

The code of the developed tool ReFlowLab using the herein presented TE model can be obtained from the following link: https://github.com/Domeml94/ReFlowLab^21^.

## Results & discussion

Based on the model described in the section Method we calculated the capital costs of 38 organic active materials. In the following, we present the obtained results and break down the individual factors that contribute to the overall costs of an RFB with the respective active material class.

To adjust for possible changes in costs due to possible optimization states of the RFB system, we discuss the results for both the AqORFB and the VRFB by means of two self-defined scenarios (cf. Table 1), with: (a) “Present Case”, using state-of-the-art values as reported in literature or given by industry/companies. This choice implies that we apply an estimated material price at the present moment given by literature. The Nafion membrane is selected as separator material. Further semipermeable materials like polybenzimidazole (PBI) or anion exchange membranes, to account for different pH values and chemistry of organic active materials, are not considered due to lack of available data and the scope of this work on a general outline of all RFB cost contributions^22^.

**Table 1:** Overview of the assumptions made for both scenarios, Present Case and Future Case, and a statement on why we have made these projections

Parameter	Present Case	Future Case	Remarks
VRFB			
*C*_repl. El_^hl^/$ kg^−1^	0	−2.41 (20.52 $ kg^−1^ V_2_O_5_) −7.45 (63.49 $ kg^−1^ V_2_O_5_) −0.65 (5.51 $ kg^−1^ V_2_O_5_)	Recycling of vanadium electrolytes is assumed to be well-established. The selected vanadium prices reflect the high price fluctuations of V_2_O_5_. See the section Energy costs of first Supplementary Note for more details.
AqORFB			
*C*_active_/$ kg^−1^	9^23^	3.48^23^	Assuming a price drop for organic molecules due to mass production.
Separator	Nafion membrane	size-selective separator	Size-selective separators are assumed to be developed and established in future AqORFBs.

(b) “Future Case”, where on the one hand the mass production of organic active materials and on the other hand the recycling of vanadium electrolytes is well-established on industrial scale. To account for a potential price drop for organic molecules due to mass production and optimized synthesis strategies we apply the active material cost proposed by Gregory et al.^23^ for AqORFBs as future state. Furthermore, to incorporate vanadium recycling we use the selected vanadium electrolyte price as future recycling value assuming no loss of material during the operational lifetime of the RFB: e.g., 20.52 $ kg^−1^ per mass active material leads to 2.41 $ kg^−1^ per mass electrolyte calculated with a solvent density of 1.24 g cm^−3^ ^24^ for 4 M H_2_SO_4_. Due to high fluctuations in the vanadium raw material price, we extrapolate a possible future vanadium price based on the historical development of the last ten years. In addition, with current research aiming to develop enhanced size-selective separators for bulky organic molecules, we assume a future state AqORFB system applying this low-price separator alternative. We use state-of-the-art literature values for the parameters *R*_ASR_ (area-specific resistance) and separator cost (see Table S1 and Table S2) in our calculations.

Table 1 specifies the assumed values and explains the conditions for each of the two scenarios used for the model evaluation.

**Fig. 1:**
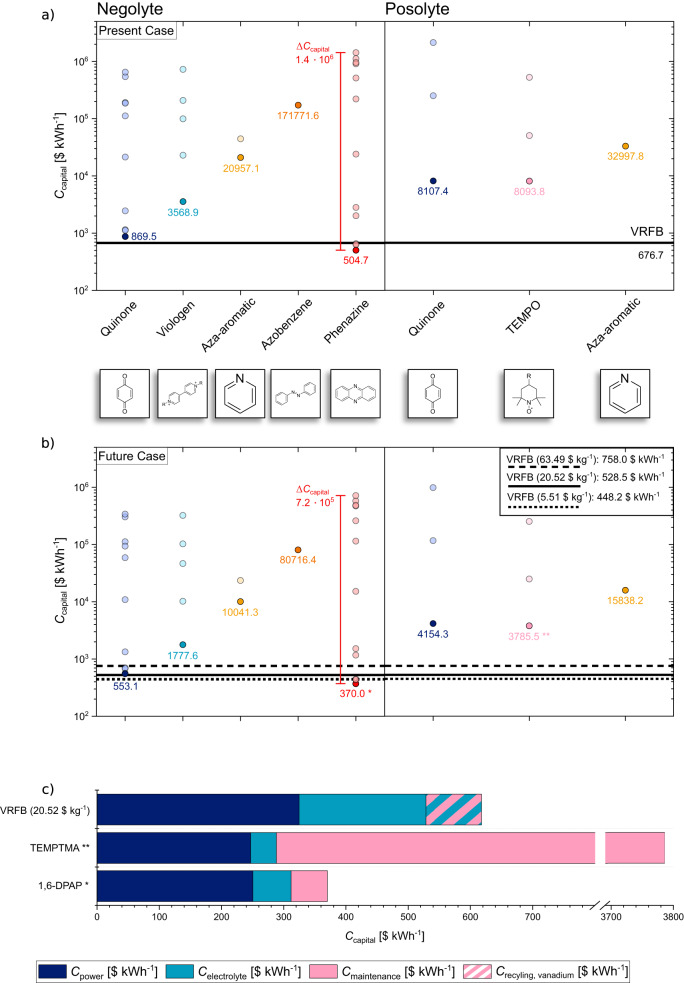
Main result of the calculations performed with the RFB cost model assuming the working point WP1 (0.1 W cm^−2^ specific power). **a** Data points representing the outcome of calculations assuming the Present Case conditions. **b** Points depicted show data obtained by applying assumptions for the Future Case scenario. Negolytes and posolytes are considered full cells with a predefined standard half-cell as counter electrode. A distinction is made between the two scenarios discussed above, Present Case and Future Case, as well as between different classes of molecules and half-cell sides, i.e., for the posolyte and the negolyte. Minimum capital cost per active material group is depicted in darker color. **c** Bar plot that shows the total cost broken down into the three main contributions for the most economical active materials per group (negolyte, posolyte, and VRFB) based on the Future Case results. The dashed field in the VRFB electrolyte cost contribution marks the recycling value of vanadium assumed for the Future Case scenario. (All data points are listed in Tables S10, S11, source data are provided as a Source Data file).

With the predefined RFB system in terms of capacity and power as well as the targeted working point (WP) and operational lifetime the capital cost of the selected active materials is calculated and visualized in Fig. 1.

Figure 1 represents the main results of the evaluated 38 organic compounds and VRFB in view of the aforementioned two different scenarios. Figure 1a, b show the resulting capital cost in $ kWh^−1^ for each active material class. A material class contains several individual derivatives of the base structure. Therefore, several values for the capital cost are displayed for one class, representing an individual derivative, with the lowest value being highlighted for each class with its numeric value. Since most literature reports on organic compounds focus on the design of either the negolyte or posolyte, we group the obtained results respectively for the cost evaluation (see also subsection Energy cost in Supplementary Note 1). The VRFB is evaluated for different material prices corresponding to the discussed scenarios shown as a baseline for better comparison.

Looking at Fig. 1a, b in detail, a wide range (approximately 400 $ kWh^−1^ to 10^6^ $ kWh^−1^) of material cost results for AqORFBs is depicted. In both cases, almost all of the 38 molecules are more expensive than the vanadium reference system (results above the reference lines of VRFB 676.7 $ kWh^−1^ Present Case, up to 758.0 $ kWh^−1^ Future Case, respectively). In the Present Case, only three phenazines yield capital costs underneath the 676.7 $ kWh^−1^ of the vanadium electrolyte, with 1,6-DPAP (3,3’-(phenazine-1,6-diylbis(azanediyl))dipropionic acid) having the lowest cost with 504.7 $ kWh^−1^.

By shifting from the present to the Future Case, two quinone negolytes exhibit lower expected costs than VRFBs in the case of a high vanadium price of 63.49 $ kg^−1^. Here, the anthraquinone DBAQ (4,4’-(9,10-anthraquinone-diyl)dibutanoic acid) scores best within this material class with expected costs of 553.1 $ kWh^−1^. Overall, 1,6-DPAP yields the minimum capital cost with 370.0 $ kWh^−1^ for the Future Case as well. Going from the Present Case to the Future Case shows that the capital cost is reduced for all active materials. Additionally, the span from minimum and maximum costs per molecule class decreases considering the Future Case, using the highlighted scope of phenazines as an example (1.4 × 10^−6^ $ kWh^−1^ in the Present Case to 7.2 × 10^−5^ $ kWh^−1^ in the Future Case). Furthermore, we can also see significantly fewer data points for the posolyte group than for the negolyte half-cell. Additionally, a lower amount of distinguishable material groups on the posolyte side is noticeable. A shift to lower cost values in the Future Case data is visible for the posolyte materials, too.

According to the observations above, the to-be-expected capital cost is highly influenced by the difference in physical and electrochemical properties of active materials. The results reveal that only few promising active materials with Future Case capital costs lower than VRFBs are reported in the literature. Thereby, 1,6-DPAP is the active material with the lowest expected cost. It might be reasonable to steer future research in the direction of finding active materials within the molecule groups of phenazines and quinones for negolytes. Pang et al.^22^ already published highly stable phenazines where the capacity loss was not detectable, further highlighting this material class but excluded in our calculations due to a missing value of the capacity faded rate.

The shown capital costs for industrial-scaled systems represent expected benchmark values due to the required assumptions in this model. The literature values of active materials used for our calculations are based on lab-scale RFB setups. These tests are usually performed under controlled conditions such as a constant room temperature and an inert gas atmosphere. The air stability of the organic active materials may serve as another criteria in assessing applicability within future studies. Maintaining an inert atmosphere to prevent parasitic oxidation of the molecules with atmospheric oxygen would increase the overall RFB costs in real applications^10,25^. A transfer of the herein-gained knowledge to an industrial scale would be particularly useful.

The shift in capital costs of negolytes, but also posolytes materials, to lower values in the future scenario shows the cost-saving potential that would come with the mass production of active materials or with vanadium recycling (see also Fig. S2). However, with the current state of technology, we are limited in displaying the actual impact of mass production because a generalized approach with equal material prices for all active materials is applied according to the self-defined cases. Additional reaction steps can increase costs when upscaling the synthesis, altering the outcome of actual achievable RFB capital costs by applying different organic active materials^26^. The synthesis of the phenazine base structure can be achieved by a 1-step condensation reaction, as shown by Hollas et al.^27^. But to produce the, within this study, favorable 1,6-DPAP, further steps are involved starting with a bromination of the phenazine precursor and functionalization by amino acids afterwards^28^.

The observation that fewer molecule classes for posolytes are discussed in literature indicates the challenge for researchers in this field. With a distinct lack of promising posolyte active materials, much effort needs to be invested into the development of new molecules with high redox potential.

To further understand the cause of the wide price range observed, analyzing merely the capital cost is not sufficient. Therefore, Fig. 1c unravels the three main contributions to *C*_capital_.

Figure 1c is a bar plot of the minimal capital cost for the Future Case scenario from each group (negolyte, posolyte) and the mid-price VRFB, showing a detailed breakdown of the cost contributions. Focusing on the power cost a difference between VRFB and the organic molecules is visible, with the VRFB showing a higher value. The parameter *C*_electrolyte_ reveals a significantly smaller contribution by the organic active materials. The posolyte TEMPTMA (N,N,N−2,2,6,6-heptamethylpiperidinyl-oxy-4-ammonium chloride) shows the lowest electrolyte cost. Moreover, using organic molecules requires maintenance costs, with TEMPTMA exhibiting a substantial cost contribution. The VRFB has negative maintenance costs due to recycling earnings marked as striped bar.

Equation (2) in the section Method shows how *C*_power_ is calculated with the parameter $${C}_{{{{{\rm{stack}}}}},{{{{\rm{system}}}}}}$$ highly influenced by the material selected. The sum of all battery stacks is scaled by the necessary electrode surface area *A* to reach the target system power as well as the area-specific stack price (see Eq. (3)). The more expensive Nafion separator increases the stack cost of VRFB (1661.02 $ per stack instead of 993.82 $ per stack for Future Case, see SI).

To better understand the differences in the electrolyte costs between the selected materials a closer look at the calculation of this summand is necessary. In Eq. (5) the electrolyte cost scales proportionally with the active material price. While a vanadium price of 20.52 $ kg^−1^ is assumed in our model, the costs of the organic materials are calculated with a value of 3.48 $ kg^−1^ for the results depicted in Fig. 1c). The differing outcome in *C*_electrolyte_ of 1,6-DPAP and TEMPTMA result from the used molecule specific parameters (see Table S8). TEMPTMA has a higher solubility (3.2 mol L^−1^ | 3.2 $${{{{{\rm{mol}}}}}}_{{{{{{\rm{e}}}}}}^{-}}{{{{{{\rm{L}}}}}}^{-1}}$$) than 1,6-DPAP (1.005 mol L^−1^ | 2.010 $${{{{{\rm{mol}}}}}}_{{{{{{\rm{e}}}}}}^{-}}$$ L^−1^), and therefore less mass of conducting salt is necessary in the electrolyte with target capacity of 4 MWh. Furthermore, 1,6-DPAP possesses a higher molar mass (354.37 g mol^−1^ vs. 249.803 g mol^−1^) leading to a higher active material mass and therefore resulting price in the calculation.

Next, the plant maintenance cost for the chosen posolyte material stands out. According to Eq. (6) the plant maintenance cost contains costs for the stack as well as the electrolyte exchange. Due to the capacity fading of 0.27% d^−1^ the TEMPTMA electrolyte needs to be exchanged frequently over the operational lifetime of the considered RFB with an annual exchange fraction *f* of 0.99 y^−1^. 1,6-DPAP in comparison is reported with a capacity fading of 0.0015% d^−1^ leading to an annual exchange of 0.005475 y^−1^ ^28,29^.

Based on the analysis above, the major benefits of organic active materials over vanadium lie in the reduced electrolyte cost as well as power cost. Plant maintenance in particular is a major concern when applying organic posolyte materials. The following paragraph further unravels this relation.

**Fig. 2:**
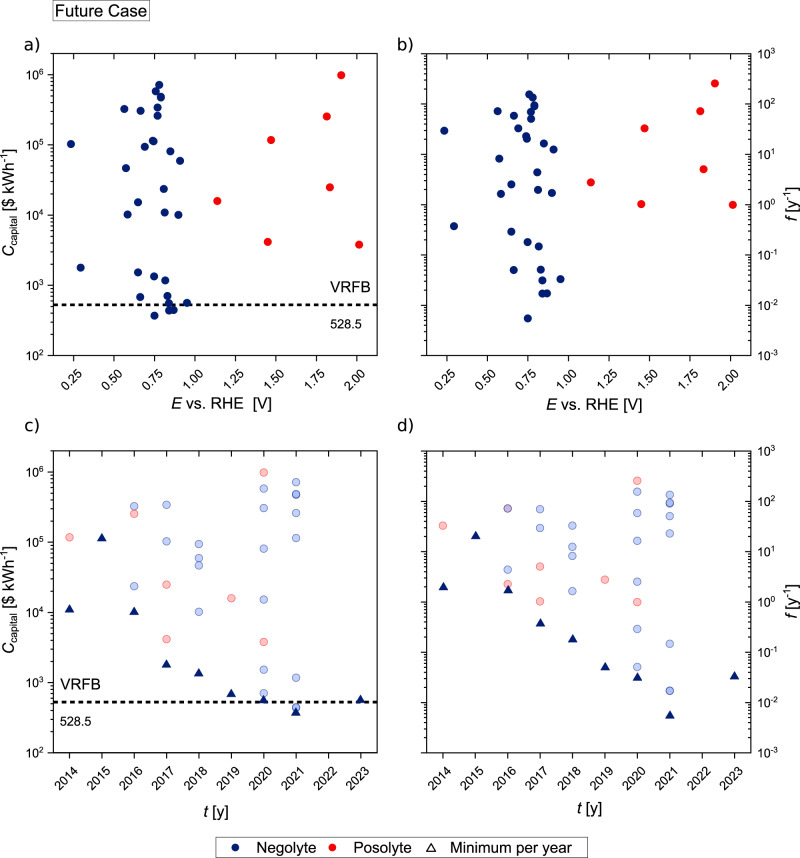
Side-by-side comparison of capital costs and capacity fade rates referenced to the redox potential or publication year of each active material using Future Case calculations. **a** Future Case capital costs *C*_capital_ and **b** capacity fade rate *f*, as a function of the active material redox potential *E* referenced to the proton redox potential at the pH value (RHE) of the individual electrolyte for negolyte and posolyte materials. **c** Overview of Future Case capital costs *C*_capital_, and **d** capacity fade rate *f*, in relation to each active materials individual publication year. The minimum capital cost value for each publication year is represented in dark color triangles while values above the minimum are depicted in lighter color (source data are provided as a Source Data file).

Figure 2a shows the future state capital costs for the active material’s normalized redox potential *E*. Here, the redox potentials are referenced to the potential of hydrogen taking the individual electrolyte pH value into account. Figure 2b shows the capacity fade rate *f* as a function of the same normalized redox potentials *E*. The future state capital costs *C*_capital_ are shown in Fig. 2c) plotted against the publication year of the literature values used to calculate the data points. Figure 2d depicts the capacity fade rate *f* of the active materials for the same years. The VRFB costs for the Future Case scenario and mid-range material cost are marked in both, Fig. 2a, c, as reference lines (dotted line). Negolyte and posolyte active materials are indicated as blue and red solid circles, respectively.

Observing the potential range of the calculated molecules of Fig. 2a the molecules with the lowest cost can be found in the mid-range redox potential of about 0.75 to around 0.85 V vs. RHE. Besides, the overall active materials results spread across the entire potential range, with materials more distant to this potential region showing higher capital costs. Particularly higher potentials above 1.1 V vs. RHE yield higher costs. This trend can be explained by the capacity fade rate shown in Fig. 2b. In both figures, potential values close to one of both ends of the depicted range lie close to the stability window of water, with 0 V vs. RHE defined as *E*(H^+^/H_2_). With the trend observed in Fig. 2a, the least cost-intensive active materials lie in the mid-range redox potential, away from the potentials at which water splitting occurs. The correlating trend of b proves that the elevated costs can in fact be traced back to stability challenges due to redox potentials close to the stability window of water. Therefore, active materials with promising redox potentials, especially for the posolyte side, might not be suitable for application due to lack of stability, which is one of the problems that has to be addressed within future research. However, it should be noted that besides the decomposition of the active materials, crossover effects could also contribute to capacity fade rates, depending on the selected measurement technique^29, 30^. Hence, the search for novel, highly selective separator materials should not be overlooked. In addition, regeneration of already decomposed electrolyte may be an option for selected active materials, further increasing their lifetime and thus reducing capacity fade relative to the overall battery lifetime^31^.

When putting the results in order of publication date (Fig. 2c) it becomes apparent that the active materials with low expected capital costs have been published only in recent years. While the capital costs are scattered over a wide range with each year of publication, the minimum projected AqORFB costs reach lower values. Molecules yielding lower cost than the benchmark value for VRFBs have not been reported before the year 2021. Comparing this plot with the capacity fade rates depicted in Fig. 2d) the same trend holds for the lower cost values. Although only recently published active materials lie below the cost of VRFB, which is the original intent of using organic active materials, it might be likely that this trend will continue. As the graph in Fig. 2d) shows a very similar curve for the capacity fade rate the visible trends in c must be connected to active material degradation. It can therefore be assumed that the stability of the molecules and the corresponding plant maintenance are the main drivers for the often very high expected costs associated with organic active materials.

Figure S3 shows the Future Case capital costs *C*_capital_ as a function of the electrolyte pH. The pH-dependent results reveal that the active materials with the lowest capital costs lie in the pH range of 8 to 12, including 1,6-DPAP at pH 8. While acidic RFBs are not represented in the data as much, a higher number of active materials can be found in neutral to alkaline pH ranges, with low-cost active materials being applied in alkaline solutions. Therefore, the alkaline pH range of 8 to 12 may be of interest for future research. It is remarkable that so far, no organic posolytes have been proposed for the alkaline pH range. With a lack of posolytes in the pH area that is most interesting for the negolytes, a suitable combination is missing to create an RFB comprising organic active materials at the both positive and negative sides. For this reason, the aforementioned pH range should have priority in future research for posolyte materials.

The inorganic ferrocyanide salt represents an inorganic posolyte that is already used in neutral to alkaline pH range. Luo et al.^32^ showed that [Fe(CN)_6_]^4-^ gains stability when combined with ammonium cations NH_4_^+^. Table S12 lists the expected cost of RFB applying (NH_4_)_4_[Fe(CN)_6_] as posolyte material. The calculation was performed for an RFB with a Nafion separator and an active material price of 1.28 $ kg^−1^ ^32^. A capital price of 651.7 $ kWh^−1^ for an RFB including an ammonium ferrocyanide electrolyte is calculated, applying the herein presented model. In comparison, the cost of the inorganic active material is significantly lower than the result for the organic TEMPTMA (3785.5 $ kWh^−1^). Those results lead to the conclusion that instead of an all-organic RFB, a combination with inorganic materials, leading to a hybrid RFB, may be a promising concept for future development.

As an additional working point, further calculations were performed based on the fixed discharge voltage efficiency (*ε*_v, d_) of 0.916. The corresponding results are depicted in Figure S5 and a detailed discussion can be found in the corresponding subsection of Supplementary Note 3. The VRFB reveals a significant increase of *C*_capital_ for both the Present Case and Future Case scenarios due to major increase in *C*_power_ and *C*_maintenance_.

At this working point, the VRFB is operating at a very low specific power of 6.31 × 10^−4^ W cm^−2^ (see the polarization curve in Fig. S6). This leads to a significant increase of necessary cell stacks (443 stacks with WP1 vs 70227 stacks for WP2) to reach the target power, increasing *C*_power_ as well as the stack replacement costs in *C*_maintenance_. The considered organic active materials are less influenced by this working point change due to improved kinetics of the electrochemical conversion (1,6-DPAP: 5.13 × 10^−4 ^cm s^−1^ ^28^ vs. VO^2+^: 3.0 × 10^−7 ^cm s^−1^ ^33^) and therefore a less prominent activation region (see Fig. S6).

Figure S7 shows a local sensitivity analysis with the input for 1,6-DPAP at WP1 (fixed specific power) as reference point. The input parameters are varied by ±50% and categorized (see section 4. Supplementary Note: Sensitivity analysis of Supplementary Information for a detailed discussion).

The influences of certain input parameters depicted in Figure S7 indicate that by adapting the properties of the best-performing organic active material 1,6-DPAP even lower *C*_capital_ might be achievable. A lower redox potential or higher solubility might help reaching the goal of low-cost organic RFBs while already optimized parameters like the annual electrolyte replacement fraction *f* show less potential for further improvement.

### Summary and perspective

A comprehensive model for redox flow batteries was introduced as a capital cost benchmarking tool for comparing various electrolyte compositions. Based on this model, we calculated the capital costs for 38 different electrolytes with organic active materials, as well as for the VRFB system, with assumptions for a present (no vanadium recycling; low production volume of organic active materials) and a future state (vanadium recycling, vanadium price fluctuations; mass production of organic active materials, size selective separator). A wide range of costs for AqORFBs was obtained, of which just a few lie close to the calculated VRFB reference values. Only the active material phenazine 1,6-DPAP would imply results below that of the vanadium system in both scenarios. Comparing the present state capital cost with the future state indicates possible cost-saving potentials. Notably overall, there was a lack of data as well as limited options for low-cost organic active materials for the positive electrode side. This deficit could be attributed to the fact that active materials with higher redox potentials are less stable and degrade faster and are thus currently an under-explored topic of research. Calculations with ferrocyanide show a less-expensive inorganic alternative as posolyte material. Additionally, we use a local sensitivity analysis to reveal that adapting the physico-chemical properties of the 1,6-DPAP active material can lead to lower capital cost results.

In conclusion, based on the costs obtained by our model proposed, it appears that AqORFBs could be a future alternative to the current commonly applied VRFB. In this regard, the molecule base structures phenazine as well as quinone show the most promising results on the negolyte side and should therefore be in focus in future research regarding this half-cell side. Furthermore, an analysis of the publication dates of the molecules considered reveals a trend that allows speculation about future low-cost active materials. However, our study also shows that the selection of active materials is still very limited and that the lack of organic posolyte materials makes the future deployment of an all-organic, aqueous RFB uncertain. Nevertheless, a hybrid cell with an inorganic posolyte, such as ferrocyanide, is a conceivable alternative.

It should be mentioned, however, that the calculations presented can only outline trends that can help to identify promising candidates rather than predict potential future systems. The input values used are based on results from laboratory tests and are therefore a limited representative for the final application. Furthermore, the selection of only two separator materials represents the present- and future-case scenarios. Further semipermeable materials might need to be considered in the following studies to reflect possible technological developments. In addition, the calculations are based on using an idealized counter electrode. It remains to be seen through upscaling tests whether the results shown can also be confirmed for industrial applications along the entire lifetime of a RFB system.

For this reason, the calculation tool developed in our study was designed to be expandable. If more and more accurate data becomes available in the future as a result of further industry reports, or if additional active materials are published, these can be added to the tool as required and further cost calculations can be performed. Combining the latest trends of artificial intelligence in material search^7,34^ and material synthesis, and testing can help to propel data acquisition regarding novel molecular structures. Adding more and more data consistently to the tool/database used herein might allow more accurate calculations in the future, and should thus be established as a regular feedback tool in upcoming RFB research, and the search for the best active material candidates.

## Method

The calculations for our study are based on a model proposed by Brushett et al.^14^, a further developed TE model by Dmello et al.^35^, which is an extension of the approach by Darling et al.^36^. Additionally, we combine the aforementioned studies with reasonable assumptions for the size and costs of redox flow stack components by Minke et al.^5,37^. Furthermore, we extend the TE model approach with a more detailed polarization model that incorporates kinetic as well as transport phenomena to account for individual differences in the physico-chemical properties of the active material. This section provides an overview of the calculation model used in our study. For an in-depth description, we refer the reader to section 1 in Supplementary Information.

We consider a comprehensive model incorporating all relevant cost contributions in detail (see the first section in Supplementary Information for all applied equations) to enable a comparison of active materials. Our study focuses on the individual properties that change while applying different active materials and compare systems in detail accounting for the individual parameters on the cell level. We only consider water-based electrolytes, i.e., the aqueous organic redox flow battery (AqORFB), due to a lack of relevant cyclization data for non-aqueous systems. Furthermore, hybrid-RFBs with non-flow counter half-cells like in the work of Xia et al.^38^ or solid capacity boosting as shown by Zanzola et al.^39^ and Huang et al.^40^ are not considered due to the additional complexity and low technical development state. Recently published separator materials (e.g., Ye et al.^41^) are also excluded from our study due to a lack of possible future market prices of those materials. Additionally, we apply an idealized counter half-cell without electrode polarization, electrolyte degradation, and the stability window of water at the individual pH value as redox potential. This approach allows in the end the comparison of RFB costs for various active materials.

For this study, we use a battery energy capacity ${E}_{{{{{\rm{d}}}}}}$ of 4 MWh and a discharge time ${t}_{{{{{\rm{d}}}}}}$ of 4 h resulting in a discharge power ${P}_{{{{{\rm{d}}}}}}$ of 1 MW as target system. A battery stack with 40 cells ${n}_{{{{{\rm{cell}}}}}}$ and a single cell electrode area ${A}_{{{{{\rm{cell}}}}}}$ of 0.06 m^2^ are assumed^5^. Furthermore, a fixed working point (WP) needs to be specified to scale the necessary number of battery stacks. In our calculation, the model is analyzed for a specific power of 0.1 W cm^−2^ (WP1)^42^. As an alternative option (see subsection Power cost in first Supplementary Note), a discharge voltage efficiency (*ε*_v, d_ = *U*_d_/*U*_OCV_) of 0.916 was used in the calculation (WP2)^36^.

In this TE model, the overall RFB price ${C}_{{{{{\rm{capital}}}}}}$ shown in Eq. (1) includes three major contributions: electrolyte cost ${C}_{{{{{\rm{electrolyte}}}}}}$, power cost ${C}_{{{{{\rm{power}}}}}}$, and the plant maintenance cost ${C}_{{{{{\rm{maintenance}}}}}}$.

1 $${C}_{{{{{\rm{capital}}}}}}\left(\frac{{{{{\rm{\$}}}}}}{{{{{\rm{kWh}}}}}}\right)={C}_{{{{{\rm{electrolyte}}}}}}+\frac{{C}_{{{{{\rm{power}}}}}}}{{t}_{{{{{\rm{d}}}}}}}+{C}_{{{{{\rm{maintenance}}}}}}$$

With: $${C}_{{{{{\rm{capital}}}}}}$$ = capital cost ($ kWh^−1^), $${t}_{{{{{\rm{d}}}}}}$$ = discharge time (h), $${C}_{{{{{\rm{electrolyte}}}}}}$$ = electrolyte cost ($ kWh^−1^), $${C}_{{{{{\rm{power}}}}}}$$ = power cost ($ kW^−1^), $${C}_{{{{{\rm{maintenance}}}}}}$$ = plant maintenance cost ($ kWh^−1^)

Herein the power cost ${C}_{{{{{\rm{power}}}}}}$ includes the costs for the cell stack and balance-of-plant equipment like pumps as well as manufacturing cost and further contributions. Therefore, it represents the overall capital cost for the completion of an RFB system without any capacity-determining contributions.

2 $${C}_{{{{{\rm{power}}}}}}\left(\frac{{{{{\rm{\$}}}}}}{{{{{\rm{kW}}}}}}\right)={C}_{{{{{\rm{stack}}}}},{{{{\rm{system}}}}}}\cdot \frac{{t}_{{{{{\rm{d}}}}}}}{{E}_{{{{{\rm{d}}}}}}}+{C}_{{{{{\rm{BOP}}}}}}+{C}_{{{{{\rm{add}}}}}}$$

With: $${C}_{{{{{\rm{stack}}}}},{{{{\rm{system}}}}}}$$ = system stack cost ($), $${C}_{{{{{\rm{BOP}}}}}}$$ = balance-of-plant cost ($ kW^−1^), $${C}_{{{{{\rm{add}}}}}}$$ = addition to capital cost ($ kW^−1^), $${E}_{{{{{\rm{d}}}}}}$$ = energy delivered by battery system (kWh)

The stack cost is scaled by the electrode area and calculated by the following equation.

3 $${C}_{{{{{\rm{stack}}}}},{{{{\rm{system}}}}}}\left({{{{\rm{\$}}}}}\right)={C}_{{{{{\rm{stack}}}}},{{{{\rm{area}}}}}}\cdot {A}_{{{{{\rm{total}}}}}}$$

With: $${C}_{{{{{\rm{stack}}}}},{{{{\rm{area}}}}}}$$ = stack cost per unit area ($ m^−2^), $${A}_{{{{{\rm{total}}}}}}$$ = electrode area (m^2^)

The electrode surface area *A* is calculated as follows:

4 $${A}_{{{{{\rm{total}}}}}}\left({{{{{\rm{m}}}}}}^{2}\right)=\frac{{E}_{{{{{\rm{d}}}}}}}{{\varepsilon }_{{{{{\rm{sys}}}}},{{{{\rm{d}}}}}}\cdot {i}_{{{{{\rm{d}}}}}}\cdot {U}_{{{{{\rm{d}}}}}}\cdot {t}_{{{{{\rm{d}}}}}}}=\frac{{P}_{{{{{\rm{d}}}}}}}{{\varepsilon }_{{{{{\rm{sys}}}}},{{{{\rm{d}}}}}}\cdot {i}_{{{{{\rm{d}}}}}}\cdot {U}_{{{{{\rm{d}}}}}}}$$

With: ${\varepsilon }_{{{{{\rm{sys}}}}},{{{{\rm{d}}}}}}$ = efficiency accounts for losses associated with auxiliary equipment (including power conversion, electrolyte pumps, and heat exchanger during discharge), ${i}_{{{{{\rm{d}}}}}}$ = discharge current density (A m^−2^), ${U}_{{{{{\rm{d}}}}}}$ = discharge cell voltage (V), ${P}_{{{{{\rm{d}}}}}}$ = discharge power (W)

To take differences in active material properties into account, a polarization curve is considered incorporating ohmic resistance, charge transfer polarization, and concentration overpotentials (see subsection power cost in first Supplementary Note for more details). Depending on the selected working point the necessary electrode surface area is calculated by Eq. (4).

The electrolyte cost ${C}_{{{{{\rm{electrolyte}}}}}}$ (Eq. (5)) includes the acquisition of active materials, conducting salt, and solvent as well as the cost for the electrolyte tanks.

5 $$	{C}_{{{{{\rm{electrolyte}}}}}}\left(\frac{{{{{\rm{\$}}}}}}{{{{{\rm{kWh}}}}}}\right)=\frac{{C}_{{{{{\rm{electrolyte}}}}}}\left({{{{\rm{\$}}}}}\right)}{{E}_{{{{{\rm{d}}}}}}} \\ 	=\frac{\frac{{{C}_{{{{{\rm{active}}}}}}}^{{{{{\rm{P}}}}}}\cdot {s}^{{{{{\rm{P}}}}}}\cdot {M}^{{{{{\rm{P}}}}}}}{{\chi }^{{{{{\rm{P}}}}}}\cdot {{z}_{{{{{\rm{e}}}}}}}^{{{{{\rm{P}}}}}}}+\frac{{{C}_{{{{{\rm{active}}}}}}}^{{{{{\rm{N}}}}}}\cdot {s}^{{{{{\rm{N}}}}}}\cdot {M}^{{{{{\rm{N}}}}}}}{{\chi }^{{{{{\rm{N}}}}}}\cdot {{z}_{{{{{\rm{e}}}}}}}^{{{{{\rm{N}}}}}}}+2\cdot {r}_{{{{{\rm{avg}}}}}}\cdot {M}_{{{{{\rm{salt}}}}}}\cdot {C}_{{{{{\rm{salt}}}}}}+\frac{2}{{b}_{{{{{\rm{avg}}}}}}}\cdot {C}_{{{{{\rm{solvent}}}}}}+{C}_{{{{{\rm{tank}}}}}}}{F\cdot {U}_{{{{{\rm{d}}}}}}\cdot {\varepsilon }_{{{{{\rm{sys}}}}},{{{{\rm{d}}}}}}\cdot {\varepsilon }_{{{{{\rm{q}}}}},{{{{\rm{rt}}}}}}}$$

With: $${r}_{{{{{\rm{avg}}}}}}$$ = mean molar salt ratio (mol mol^−1^) $$\left({r}_{{{{{\rm{avg}}}}}}=\frac{{r}^{{{{{\rm{P}}}}}}+{r}^{{{{{\rm{N}}}}}}}{2}\right)$$, $${b}_{{{{{\rm{avg}}}}}}$$ = mean actives molality (mol kg^−1^) $$\left({b}_{{{{{\rm{avg}}}}}}=\frac{2{b}^{{{{{\rm{P}}}}}}{b}^{{{{{\rm{N}}}}}}}{{b}^{{{{{\rm{P}}}}}}+{b}^{{{{{\rm{N}}}}}}}\right)$$, $${C}_{{{{{\rm{salt}}}}}}$$ = salt cost per unit mass ($ kg^−1^), $${C}_{{{{{\rm{solvent}}}}}}$$ = solvent cost per unit mass ($ kg^−1^), $${C}_{{{{{\rm{tank}}}}}}$$ = electrolyte tank costs per quantity ($ mol^−1^), *M*^s^ = molecular weight of species s (g mol^−1^) (P: Posolyte; N: Negolyte), *s*^s^ = stoichiometric coefficient of species s, $${\varepsilon }_{{{{{\rm{q}}}}},{{{{\rm{rt}}}}}}$$ = round-trip coulombic efficiency, $$\chi$$ = maximum SOC range, $${z}_{{{{{\rm{e}}}}}}$$ = number of electrons per battery storage reaction

The plant maintenance cost ${C}_{{{{{\rm{maintenance}}}}}}$ is the third major expense accounted for in this study by Eq. (1). Here, we separate maintenance cost due to necessary electrolyte exchange and stack replacement with parameters ${C}_{{{{{\rm{NPV}}}}},{{{{\rm{replacement}}}}}}$ and ${C}_{{{{{\rm{NPV}}}}},{{{{\rm{stack}}}}}}$, respectively.

6 $${C}_{{{{{\rm{maintenance}}}}}}\left(\frac{{{{{\rm{\$}}}}}}{{{{{\rm{kWh}}}}}}\right)={C}_{{{{{\rm{NPV}}}}},{{{{\rm{replacement}}}}}}+{C}_{{{{{\rm{NPV}}}}},{{{{\rm{stack}}}}}}$$

### Supplementary information

Supplementary Information

### Source data

Source Data

## Data Availability

All data generated in this study are included in the manuscript and Supplementary Information file. Source data are provided in this paper.

## Code availability

The calculation tool used to calculate the shown results is available via GitHub repository: https://github.com/Domeml94/ReFlowLab^21^.
