# Supplementary material for: Benchmarking organic active materials for aqueous redox flow batteries in terms of lifetime and cost
Source: Nat Commun. 2023 Oct 21;14:6672. doi: 10.1038/s41467-023-42450-9 (PMC10590391; doi:10.1038/s41467-023-42450-9)
Supplement: Supplementary file 1 — Supplementary Information [file 41467_2023_42450_MOESM1_ESM.pdf]

Supplementary Information

Benchmarking organic active materials for aqueous redox flow batteries in terms of lifetime and cost

Dominik Emmel[1], Simon Kunz[2,3], Nick Blume[4, 5], Yongchai Kwon[6], Thomas Turek[5,7], Christine Minke[4, 5]*, Daniel Schröder[1]*

[1] Institute of Energy and Process Systems Engineering (InES), Technische Universität Braunschweig, Langer Kamp 19B, D-38106 Braunschweig, Germany

[2] Institute of Physical Chemistry, Justus-Liebig-University Giessen, Heinrich-Buff-Ring 17, D-35392 Giessen, Germany

[3] Center for Materials Research, Justus-Liebig-University Giessen, Heinrich-Buff-Ring 16, D-35392 Giessen, Germany

[4] Institute of Mineral and Waste Processing, Recycling and Circular Economy Systems, Clausthal University of Technology, Walther-Nernst-Str. 9, D-38678 Clausthal-Zellerfeld, Germany

[5] Research Center Energy Storage Technologies, Am Stollen 19A, D-38640 Goslar, Germany

[6] Department of Chemical and Biomolecular Engineering, Seoul National University of Science and Technology, 232 Gongneung-ro, Nowon-gu, Seoul, 01811, Republic of Korea

[7] Institute of Chemical and Electrochemical Process Engineering, Clausthal University of Technology, Leibnizstraße 19, D-38678 Clausthal Zellerfeld, Germany

* corresponding authors: christine.minke@tu-clausthal.de;

d.schroeder@tu-braunschweig.de

1. Supplementary Note: Method – Model description

In this section the model applied in this work is explained in more detail. The overall RFB price C_{capital} shown in equation (S1) includes three major contributions: electrolyte cost $C_{\text{electrolyte}}$, power cost C_{power} and the plant maintenance cost $C_{\text{maintenance}}$.

$$C_{\text{capital}}\left(\frac{\$}{\text{kWh}}\right) = C_{\text{electrolyte}} + \frac{C_{\text{power}}}{t_{\text{d}}} + C_{\text{maintenance}} \tag{S1}$$

With: C_{capital} = capital cost (\$ kWh^{-1})

t_{d} = discharge time (h)

$C_{\text{electrolyte}}$ = electrolyte cost (\$ kWh^{-1})

C_{power} = power cost (\$ kW^{-1})

$C_{\text{maintenance}}$ = plant maintenance cost (\$ kWh^{-1})

Power cost:

The power cost C_{power} includes the stack cost of the overall RFB system $C_{\text{stack, system}}$. This quantity accounts for all contributions by the RFB stack in the considered battery system. It is expected that the time necessary for full charging will exceed the time for discharging. Therefore, as in the model by Darling et al.[1], we assume that the electrode area is determined by the discharging process of RFB applications. This implies that the discharging power density is the limiting factor. Besides the RFB stack cost, the power cost also includes additional cost C_{add} and cost generated due to balance-of-plant equipment C_{bop}. With the cost factor C_{bop}, the cost of all additional components necessary for operating the RFB plant such as pumps, pipes, power conditioning equipment, controls, sensors, fans, filters, valves and heat exchangers, is calculated by the model suggested by Darling et al.[2]. C_{add} is the markup of material costs to the total system price. This parameter includes manufacturing cost, sales, administration cost, research and development cost as well as profit quantities. By implication C_{power} represents the overall capital cost for the completion of a RFB system without any capacity determining contributions.

The total system costs per power are given by equation (S2).

$$C_{\text{power}}(\$/\text{kW}) = C_{\text{stack, system}} \cdot \frac{t_{\text{d}}}{E_{\text{d}}} + C_{\text{bop}} + C_{\text{add}} \tag{S2}$$

With: $C_{\text{stack, system}}$ = system stack cost ($)

C_{bop} = balance-of-plant cost ($ kW^{-1})

C_{add} = addition to capital cost ($ kW^{-1})

E_{d} = energy discharged by battery system (kWh)

The stack cost is scaled by the electrode area and calculated by the following equation.

$$C_{\text{stack, system}} = C_{\text{stack, area}} \cdot A_{\text{total}} \tag{S3}$$

With: $C_{\text{stack, area}}$ = stack cost per unit area ($ m^{-2})

A_{total} = electrode area (m^2)

Brushett et al.[3] apply in their techno-economic (TE) model benchmark values as cost per power conversion unit. However, in our study, we calculate the individual stack cost based on a method by Minke et al.[4]. The power subsystem of RFBs is comprising of cell stacks. To scale the cost per stack, a general measure for an industrial application is defined with 40 cells n_{cell} each with a single cell electrode area A_{cell} of 0.06 m^2.

With this, the total number of cells n_{total} can be calculated with the total surface area A and the active area of each individual cell A_{cell}:[4]

$$n_{\text{total}} = \frac{A_{\text{total}}}{A_{\text{cell}}} \tag{S4}$$

$$n_{\text{stack}} = \frac{n_{\text{total}}}{n_{\text{cell}}} \tag{S5}$$

The component costs necessary to calculate the overall stack cost are listed in Table S1.

Table S1: List of cost input parameters for stack components (single cell area of 0.06 m²), euro values converted to dollars at the exchange rate on 12.08.22.

Component	Material	Thickness	Number	Unit cost	Reference
**Membrane (vanadium)**	Nafion N-117	183 µm	n_{total}	(300 € m^{-2})<br>308 \$ m^{-2}	[5]
**Membrane (AqORFB)**	Size-selective separator	-	n_{total}	30 \$ m^{-2}	[6]
**Felt electrode**	GFD4.6	5 mm	2 n_{total}	(50 € m^{-2})<br>51 \$ m^{-2}	[5]
**Bipolar plate, flow field**	PPG86	5 mm	n_{total} + n_{stack}	(100 € m^{-2})<br>103 \$ m^{-2}	[5]
**Current collector**	Cu	5 mm	2 n_{stack}	(16 €)<br>16 \$ per piece	[5]
**Stack frame**	PVC (PVC housing)		n_{stack}	(300 €) 308 \$ per stack	[5]
**Assembly**				(0.50 €) 0.51 \$ per component (83.64 \$ per stack)	[5]

The use of size-selective separators as a cost-effective alternative replacing the common Nafion separator could be incorporated by applying larger organic molecules as active material of AqORFBs. However, since studies on this type of separator were rarely conducted so far, a possible application in industrial scale and thus a competitive price is not available at the moment. For the calculation, using a size-selective separator is assumed as a solution for the Future Case scenario, while Nafion is considered as second-best option, which corresponds to the Present Case. Accounting for the values listed in Table S1 the cost of a VRFB stack is 692.09 \$ m^{-2} (1661.02 \$ per stack), while when organic active materials are used, the cost for the stack in a Future Case situation is reduced to 414.09 \$ m^{-2} (993.82 \$ per stack).

The overall electrode size for planning a battery system is determined by the battery energy capacity E_d and discharge time t_d which decides the target discharge power P_d which is a target parameter. To specify the battery model, we define an industrial system with a capacity of 4 MWh and a discharge time t_d of 4 h to set the discharge power P_d to 1 MW. Furthermore, a specific working point is used to calculate the surface area of the electrode. In our calculation the battery model for each individual electrolyte is analyzed for a specific power of 0.1 W cm^{-2}.[7] As alternative option, a discharge voltage efficiency ($\varepsilon_{v,d} = U_d/U_{OCV}$) of 0.916 was used in the calculation.[1]

The electrode surface area A_{total} is calculated as follows:

$$A_{\text{total}} = \frac{E_{\text{d}}}{\varepsilon_{\text{sys, d}} \cdot i_{\text{d}} \cdot U_{\text{d}} \cdot t_{\text{d}}} = \frac{P_{\text{d}}}{\varepsilon_{\text{sys, d}} \cdot i_{\text{d}} \cdot U_{\text{d}}} \tag{S6}$$

With: E_{d} = energy delivered by the battery system (kWh)

$\varepsilon_{\text{sys, d}}$ = efficiency accounts for losses associated with auxiliary equipment (including power conversion, electrolyte pumps and heat exchanger during discharge)

i_{d} = discharge current density (A m^{-2})

U_{d} = discharge cell voltage (V)

P_{d} = discharge power (W)

To address the difference in polarization curves for different active material, a suitable polarization curve should be applied to determine the discharge current density i_{d} and the resulting cell voltage V_{d}. Equation (S7) describes the calculation of the discharge voltage U_{d} and includes all resistances considered in this work.

$$U_{\text{d}} = U_{\text{OCV}} - \eta_{\text{ct}} - \eta_{\text{ohm}} - \eta_{conc} \tag{S7}$$

With: U_{OCV} = thermodynamically reversible potential (V)

η_{ct} = charge transfer overpotential (V)

η_{ohm} = ohmic overpotential (V)

η_{diff} = diffusion overpotential (V)

The open cell voltage (OCV) U_{OCV} is calculated by the Nernst equation (see formula (S10)). In our study, an approximate polarization model including simplified equations for ohmic resistance, charge transfer polarization as well as concentration overpotentials is assumed. The charge transfer overpotential η_{ct} describes the polarization of the electrode occurring due to the electrochemical conversion of the active materials at the electrode, while the ohmic overpotential η_{ohm} generally accounts for the two main contributions: the ionic conductivity of electrolyte and separator. Additionally, the mass transfer of the active material is coupled with the charge transfer via the concentration overpotential η_{conc}. To facilitate the computation a state of charge (SOC) value of 0.5 is used. Additional losses due to the battery stack like shunt currents are being neglected.[8,9]

The generalized half cell reactions of a simple redox process taking place in a battery system is written as follows:

$$\frac{z}{n_{\mathrm{red}}}\left(\mathrm{A_{red}}^{a} \rightarrow \mathrm{A_{ox}}^{(a+n_{\mathrm{red}}-x)} + n_{\mathrm{red}}\ \mathrm{e}^{-} + x\ \mathrm{H}^{+}\right)$$
$$= \nu_{\mathrm{N}}\ \mathrm{A_{red}}^{a} \rightarrow \nu_{\mathrm{N}}\ \mathrm{A_{ox}}^{(a+n_{\mathrm{red}}-x)} + z\ \mathrm{e}^{-} + z_x\ \mathrm{H}^{+} \quad \text{(S8)}$$

$$\frac{z}{n_{\mathrm{ox}}}\left(\mathrm{C_{ox}}^{b} + n_{\mathrm{ox}}\ \mathrm{e}^{-} + y\ \mathrm{H}^{+} \rightarrow \mathrm{C_{red}}^{(b-n_{\mathrm{ox}}+y)}\right)$$
$$= \nu_{\mathrm{P}}\ \mathrm{C_{ox}}^{b} + z\ \mathrm{e}^{-} + z_y\ \mathrm{H}^{+} \rightarrow \nu_{\mathrm{P}}\ \mathrm{C_{red}}^{(b-n_{\mathrm{ox}}+y)} \quad \text{(S9)}$$

With: $\nu_{\mathrm{s}} = \frac{z}{n_{\mathrm{s}}}$ = stoichiometric coefficient of species s

z = numbers of electrons transferred in one battery reaction step

N = negolyte reactant

P = posolyte reactant

n_{s} = numbers of electrons transferred in one elementary reaction step

x, y = numbers of protons involved in one elementary reaction step

With the aforementioned equation, the Nernst equation for a RFB full cell is:

$$U_{\mathrm{OCV}} = E^{0}_{\mathrm{P}} - E^{0}_{\mathrm{N}} - \frac{RT}{zF}\ln\left(\frac{c_{\mathrm{P,\,red}}^{\nu_{\mathrm{P}}}}{c_{\mathrm{P,\,ox}}^{\nu_{\mathrm{P}}}\ c_{\mathrm{H+}}^{y}}\ \frac{c_{\mathrm{N,\,ox}}^{\nu_{\mathrm{N}}}\ c_{\mathrm{H+}}^{x}}{c_{\mathrm{N,\,red}}^{\nu_{\mathrm{N}}}}\right) \quad \text{(S10)}$$

With: E^{0}_{s} = standard redox potential of species s [V]

R = universal gas constant [J mol^{-1} K^{-1}]

T = temperature [K]

z = numbers of electrons transferred in one reaction step

F = Faraday constant [C mol^{-1}]

c_{s} = concentration of species s [mol L^{-1}]

The activation polarization η_{ct} is calculated by using the Butler-Volmer kinetic.[10]

$$i_d = i_{0,\,P}\left(e^{\frac{(1-\alpha_{P,\,red})\,n_P F\ \eta_{ct,\,P}}{RT}} - e^{-\frac{\alpha_{P,\,red}\,n_P F\ \eta_{ct,\,P}}{RT}}\right) \tag{S11}$$

$$i_d = i_{0,\,N}\left(e^{\frac{\alpha_{N,\,ox}\,n_N F\ \eta_{ct,\,N}}{RT}} - e^{-\frac{(1-\alpha_{N,\,ox})\,n_N F\ \eta_{ct,\,N}}{RT}}\right) \tag{S12}$$

$$\eta_{ct} = \eta_{ct,\,P} + \eta_{ct,\,N} \tag{S13}$$

With: $\alpha_{P,\,red}$ = charge transfer coefficient of positive electrode side

$\alpha_{N,\,ox}$ = charge transfer coefficient of negative electrode side

i_d = current density [A cm^{-2}]

$i_{0,\,P}$ = exchange current density of positive electrode side [A cm^{-2}]

$i_{0,\,N}$ = exchange current density of negative electrode side [A cm^{-2}]

n_P = number of electrons transferred in one reaction step positive electrode side

n_N = number of electrons transferred in one reaction step negative electrode side

$\eta_{ct,\,P}$ = charge transfer overpotential of positive electrode [V]

$\eta_{ct,\,N}$ = charge transfer overpotential of negative electrode [V]

The exchange current density is proportional to the concentration of reduced and oxidized species at the electrode surface. In our model the bulk concentration of the electrolytes applied at SOC of 0.5 is considered assuming a fast charge transfer kinetic in comparison to the mass transfer process of active material to the electrode. To determine the exchange currents for the individual active material the standard reaction rate k_0 and charge transfer coefficient α_i should be determined. These are usually obtained by using Koutecký–Levich analysis of LSV studies with a rotating disc electrode.[11,12] Literature values for reaction rates and charge transfer coefficients where preferably taken from the matching process. This means the values for the reduction reaction were taken for the cathode, while the ones for the oxidation reaction were from the anode. In case literature values were only available in opposite direction of the electrochemical reaction, these reaction rate constants were adopted for the calculation under consideration and corresponding charge transfer coefficients were calculated with equation (S16).

$$i_{0,P} = n_P\, F\, k_{0,\,ox}\, c_{ox,\,P}{}^{1-\alpha_{ox}} c_{red,\,P}{}^{\alpha_{ox}} \tag{S14}$$

$$i_{0,N} = n_N\, F\, k_{0,\,red}\, c_{red,\,N}{}^{1-\alpha_{red}} c_{ox,\,N}{}^{\alpha_{red}} \tag{S15}$$

$$\alpha_{ox} = 1 - \alpha_{red} \tag{S16}$$

With: $k_{0,\,ox}$ = reaction rate for the reduction reaction of the oxidized species of the cathode half-cell side [cm s^{-1}]

$k_{0,\,red}$ = reaction rate for the oxidation reaction of the reduced species of the anode half-cell side [cm s^{-1}]

$c_{ox,\,hc}$ = bulk concentration of oxidized species for half-cell hc [mol L^{-1}]

$c_{red,\,hc}$ = bulk concentration of reduced species for half-cell hc [mol L^{-1}]

The concentration polarization η_{conc} considers the deviation between the concentration of active material near electrode surface and the concentration in bulk solution. This relation is expressed as function of surface concentration c_{surf} or limiting current density i_{limit}.[11,13]

$$\eta_{conc,\,hc} = \frac{R\,T}{z\,F} v_p \ln\left(\frac{c_{surf,\,p}}{c_{0,\,p}}\right) = \frac{R\,T}{z\,F} v_p \ln\left(1 - \frac{i_d}{i_{limit,\,p}}\right) \tag{S17}$$

$$c_{surf,\,p} = c_{0,\,p} + \frac{\delta_p \cdot i_d}{D_p \cdot F} \cdot \frac{v_p}{z} \tag{S18}$$

$$i_{limit,\,p} = n\,F\,D_p\,\frac{c_{0,\,p}}{\delta_p} \tag{S19}$$

$$\eta_{conc} = \eta_{conc,\,C} + \eta_{conc,\,A} \tag{S20}$$

With: $\eta_{conc,\,hc}$ = concentration polarization of half-cell side hc (V)

v_p = stoichiometric constant of component p

$c_{surf,\,p}$ = near electrode surface concentration of component p (mol L^{-1})

$c_{0,\,p}$ = solution bulk concentration of component p (mol L^{-1})

δ_p = diffusion layer thickness (m)

D_p = diffusion coefficient of component p (cm^2 s^{-1})

RFBs are operated with electrolyte being pumped via forced convection. When non-slipping conditions are assumed at the electrode surface, a thin layer of non-moving electrolyte is present

whose thickness depends on the applied flow velocity as well as flow behavior of the electrode medium. For our calculation, a linear electrolyte flow velocity of 0.101 m s^{-1} proposed by Watt-Smith et al. is assumed.[14] To simply describe the complex flux behavior of a felt electrode, the Sherwood equation is applied. Here, the dimensionless Sherwood number Sh_p (see equation (S23)) describes a ratio of convective mass transfer and diffusive mass transfer. This is expressed in terms of Reynolds number Re (see equation (S22)), which characterizes the present flow pattern, and the Schmidt number Sc_p (equation (S21), which gives a ratio of kinematic viscosity and diffusion coefficient. In addition, the flow behavior in porous electrode materials can be characterized by a characteristic length; here a fiber diameter of 10 µm is considered for the characteristic length. By applying equation (S23), a correlation taken from Becker 2020, the Sherwood number for a carbon felt electrode material can be calculated.[15] This number is adopted in formula (S24) to determine the diffusion layer thickness, in the end yielding the concentration polarization.

$$Sc_p = \frac{\mu_{el,\,hc}}{D_p \cdot \rho_{el,\,p}} \tag{S21}$$

With: $\mu_{el,\,hc}$ = Electrolyte viscosity of half-cell hc (Pa s)

$\rho_{el,\,hc}$ = Density of the electrolyte of half-cell hc (kg m^{-3})

$$Re_{hc} = \frac{u \cdot \rho_{el,\,hc} \cdot d_{fiber}}{\mu_{el,\,hc}} \tag{S22}$$

With: u = Electrolyte flow velocity (m s^{-1})

d_{fiber} = carbon fiber diameter (m)

$$Sh_p = 0.07\ Re_{hc}^{\ 0.66}\ Sc_p^{\ 0.45} \tag{S23}$$

$$\delta_p = \frac{d_{fiber}}{Sh_p} \tag{S24}$$

To account for the IR drop, a zero-gap cell assembly in which the ionic conductivity of the electrolyte can be neglected is assumed. With a high electrically conductive electrode material, the main contribution to ohmic polarization is due to the ionic conductivity of the separator in the cell.

$$\eta_{ohm} = i_d \cdot R_{ASR} \tag{S25}$$

Due to a lack of data, the same resistance for each individual separator listed in Table S2 was used. Here, it is needed that vanadium electrolyte is connected with a Nafion membrane due to the crossover issues of vanadium ions, while separators for bigger organic molecules can be expected to be available in the Future Case as a cheaper, size-selective separator.

Table S2: Area-specific resistance values (R_{ASR}) for considered separator materials in this study.

Separator	R_{ASR} / Ohm cm^{-2}	Ref.
Nafion	1	[3]
Size-selective separator	0.42	[6]

Applying a specific power as working point enables to directly calculate the electrode surface. With a target discharge voltage efficiency as working point, the total overpotential of RFB cell is defined and the appropriate current density for each RFB cell system can be calculated. Using equation (S6) results in the necessary electrode surface area to the preset working point and thus enables the determination of costs for a power conversion unit.

Besides the individual stack cost, also the balance-of-plant costs C_{BOP} vary depending on the active material selected. This behavior is because C_{BOP} incorporates cost contributions that scale with the system size. For a better comparison of different RFB systems, C_{BOP} for each individual case was calculated by equation (S26). The input parameters used for this calculation were taken from Minke et al.[5] and shown in Table S3.

$$C_{BOP} = C_{HE} + C_{Inverter} + C_{Pipelines,\ fittings} + C_{cabling} + \frac{C_{process}}{P_d} + C_{Pumps} \tag{S26}$$

With: C_{BOP} = Balance-of-plant costs (\$/kW)

C_{HE} = heat exchanger costs (\$/kW)

$C_{Inverter}$ = costs for power inverters (\$/kW)

$C_{Pipelines,\ fittings}$ = costs for pipelines and fittings (\$/kW)

$C_{cabling}$ = cost for cabling (\$/kW)

$C_{process}$ = Process control system costs (\$ per battery system)

C_{Pumps} = pump costs (\$/kW)

The cost for necessary pumps C_{pump} scales with the number of RFB cell stack. With the number of pumps the required pump power is also specified, which is taken into account with energy efficiency $\varepsilon_{sys,\ d}$ of the battery's individual system. Regarding $\varepsilon_{sys,\ d}$, according to Milshtein et al.[6] the impact of change in energy requirement on $\varepsilon_{sys,\ d}$ is usually small and thus is neglected in this work.

$$C_{pump} = \frac{n_{pump\ per\ stack} \cdot C_{pump\ per\ item} \cdot n_{stack}}{P_d} \tag{S27}$$

With: $n_{pump\ per\ stack}$ = number of pumps per stack (in this work we use a value of 2)

$C_{pump\ per\ item}$ = Pump costs per item (\$ per item)

n_{stack} = number of stacks

Table S3: Cost input values for various peripherals taken from Minke et al.[5], euro values converted to dollars with *the exchange rate* from *12.08.22.*

Parameter	Symbol	Value	Ref.
**heat exchanger costs**	C_{HE}	411 $/m² plate (400 €/m² plate)<br>With 27 m² per MW: 11 $/kW	[5]
**costs for power inverters**	$C_{Inverter}$	103 $/kW (100 €/kW)	[5]
**costs for pipelines and fittings**	$C_{Pipelines,\ fittings}$	25 $/kW (24 €/kW)	[5]
**costs for cabling**	$C_{cabling}$	2 $/kW (2 €/kW)	[5]
**Process control system costs**	$C_{process}$	153,960 $ per battery system (150,000 €)	[5]
**Pump costs per item**	$C_{pump\ per\ item}$	205 $ per item (200 €/item)	[5]

Energy costs:

$C_{electrolyte}$ in formula (S1) describes the cost contributions corresponding to the total amount of electrochemically stored energy in terms of capacity provided by the considered battery. This function sums up the overall electrolyte costs including active material, salt, and solvent for negolyte and posolyte. The cost of electrochemically stored capacity in form of active material is determined by equation (S28).

$$C_{active}(\$) = C_{active}{}^{P} \cdot m_{active}{}^{P} + C_{active}{}^{N} \cdot m_{active}{}^{N} \qquad \text{(S28)}$$

With: $C_{active}{}^{P}$ = Costs per mass of positive active material ($ kg^{-1})

$m_{active}{}^{P}$ = Mass of positive active material (kg)

$C_{active}{}^{N}$ = Costs per mass of negative active material ($ kg^{-1})

$m_{active}{}^{N}$ = Mass of negative active material (kg)

Here, by using Faraday's law, a correlation of the conducted electrical charges and the corresponding mass of active material is established. Equation (S29) shows this relation with an example of one species, while the round-trip coulombic efficiency is determined by a ratio of charge and discharge capacity that are connected to external power grid.[1]

$$m_{\text{active}} = \frac{M\, s\, Q_{\text{c}}}{z_{\text{e}}\, F\, \chi} = \frac{M\, s\, Q_{\text{d}}}{\varepsilon_{\text{q, rt}}\, z_e\, F\, \chi} = \frac{M\, s\, E_{\text{d}}}{\varepsilon_{\text{sys, d}}\, U_{\text{d}}\, \varepsilon_{\text{q, rt}}\, z_{\text{e}}\, F\, \chi} = \frac{MW\, s\, E_{\text{d}}}{\varepsilon_{\text{sys, d}}\, U_{\text{d}}\, \varepsilon_{\text{q, rt}}\, z_{\text{e}}\, F\, \chi} \tag{S29}$$

With: M = molecular weight (g mol^{-1})

s = stoichiometric coefficient

Q_{c} = charge capacity (C)

Q_{d} = discharge capacity (C)

$z_{\text{e}} = n_{\text{e}}^{\text{P}} \cdot n_{\text{e}}^{\text{N}}$ = number of electrons per battery storage reaction

F = Faraday constant (C mol^{-1})

χ = maximum SOC range

$\varepsilon_{\text{q, rt}}$ = round-trip coulombic efficiency

U_{OCV} = thermodynamically reversible potential (V)

The overall electrolyte cost can be calculated by the sum of active material cost C_{active} and further electrolyte cost consisting of solvent and salt costs compiled as parameters of $C_{\text{electrolyte}}^{\text{P}}$ and $C_{\text{electrolyte}}^{\text{N}}$ for posolyte and negolyte side, with the adoption of solubility L of each active material, $L = m_{\text{active}} / m_{\text{electrolyte}}$.

$$C_{\text{electrolyte}}(\$) = \left(C_{\text{active}}^{\text{P}} + \frac{C_{\text{electrolyte}}^{\text{P}}}{L_{\text{P}}}\right) \cdot m_{\text{active}}^{\text{P}} + \left(C_{\text{active}}^{\text{N}} + \frac{C_{\text{electrolyte}}^{\text{N}}}{L_{\text{N}}}\right) \cdot m_{\text{active}}^{\text{N}} \tag{S30}$$

The model by Darling et al.[1] stops at this level of detail and does not consider the individual cost items due to solvent and different salt concentrations as well as their costs, for which Dmello et al.[2] have extended the method. The additional electrolyte costs $C_{\text{electrolyte}}^{\text{C}}$ and $C_{\text{electrolyte}}^{\text{A}}$ can be written as the sum of salt costs and costs due to the solvent:

$$C_{\text{electrolyte}}^{\text{hc}}(\$) = L_{\text{salt}}^{\text{hc}} \cdot C_{\text{salt}}^{\text{hc}} + \left(1 - L_{\text{salt}}^{\text{hc}}\right) \cdot C_{\text{solvent}}^{\text{hc}} \tag{S31}$$

With: $L_{\text{salt}}^{\text{hc}}$ salt mass ratio to electrolyte mass of half cell hc (kg kg^{1}), $L_{\text{salt}}^{\text{hc}} = \frac{m_{\text{salt}}^{\text{hc}}}{m_{\text{salt}}^{\text{hc}} + m_{\text{solvent}}^{\text{hc}}}$

$C_{\text{salt}}^{\text{hc}}$ = salt cost per unit mass (\$ kg^{-1})

$C_{\text{solvent}}^{\text{hc}}$ = solvent cost per unit mass (\$ kg^{-1})

The molar salt ratio of the individual electrolyte is calculated by the amount of substances ratio of salt to active material, shown in formula (S32).

$$r^{hc}\left(\frac{mol}{mol}\right) = \frac{n_{salt}}{n_{active}} = \frac{s^{hc}\, M^{hc}\, {L_{salt}}^{hc}}{\chi^{hc}\, {z_e}^{hc}\, {M_{salt}}^{hc}\, L_{hc}} \quad \text{(S32)}$$

Furthermore, the individual electrolyte active species molality is determined by equation (S33).

$$b^{hc}\left(\frac{mol}{kg}\right) = \frac{n_{active}}{m_{solvent}} = \frac{\chi^{hc}\, {z_e}^{hc}\, L_{hc}}{s^{hc}\, M^{hc}\left(1 - {L_{salt}}^{hc}\right)} \quad \text{(S33)}$$

Since the concentration c is a more common value in literature than a mass ratio L, the density ρ of specific electrolyte is used to determine the mass ratio of the evaluated system (more details in SI):

$$L = c \cdot M/\rho \quad \text{(S34)}$$

Inserting term (S29) for each electrochemically active species in equation (S30) in combination with the by Dmello et al.[2] suggested extensions for the additional electrolyte costs ${C_{electrolyte}}^{C}$ and ${C_{electrolyte}}^{A}$, shown in equation (S31) to (S33), leads to the expression in equation (S35) for the overall costs of total amount of electrochemical stored energy capacity. We added additional costs for the electrolyte vessels embodied in parameter C_{tank} as it scales with energy. For the electrolyte tank we assume 411 \$ m^{-3} (400 € m^{-3}) with a maximum fill level of 80%.

$$C_{electrolyte}\left(\frac{\$}{kWh}\right) = \frac{C_{electrolyte}(\$)}{E_d} = \frac{\frac{{C_{active}}^{P} \cdot s^{P} \cdot M^{P}}{\chi^{P} \cdot {z_e}^{P}} + \frac{{C_{active}}^{N} \cdot s^{N} \cdot M^{N}}{\chi^{N} \cdot {z_e}^{N}} + 2 \cdot r_{avg} \cdot M_{salt} \cdot C_{salt} + \frac{2}{b_{avg}} \cdot C_{solvent} + C_{tank}}{F \cdot U_d \cdot \varepsilon_{sys,\,d} \cdot \varepsilon_{q,\,rt}} \quad \text{(S35)}$$

With: r_{avg} = mean molar salt ratio (mol mol^{-1}), $r_{avg} = \frac{r^{P} + r^{N}}{2}$

b_{avg} = mean actives molality (mol kg^{-1}), $b_{avg} = \frac{2\, b^{P}\, b^{N}}{b^{P} + b^{N}}$

C_{salt} = salt cost per unit mass (\$ kg^{-1})

$C_{solvent}$ = solvent cost per unit mass (\$ kg^{-1})

C_{tank} = electrolyte tank costs per quantity (\$ mol^{-1})

$$C_{tank} = \frac{{C_{tank}}^{P}}{{c_L}^{P} \cdot x_V} + \frac{{C_{tank}}^{N}}{{c_L}^{N} \cdot x_V} \quad \text{(36)}$$

With: ${C_{tank}}^{P}$ (${C_{tank}}^{N}$) = tank cost per volume for positive (negative) half-cell side (\$ m^{-3})

${c_L}^{P}$ (${c_L}^{N}$) = solubility of species on positive (negative) half-cell side (mol m^{-3})

x_V = max. fill level of tank

There is currently a wide range of organic molecules discussed in literature for future RFB applications. The synthetic routes of the selected organic active materials differ widely, with various groups of active materials like quinones, TEMPOL derivatives or phenazines as well as different substituents as options. Furthermore, upscaling the active material production will have a significant influence on the to be expected material price. The prediction of this factor is not trivial. Therefore, further studies are needed to determine the actual material prices for organic active materials in RFB application.

Darling et al.[1] estimated within their TE study that a target active material price of ≤ 5 $ kg^{-1} is necessary to reach the recommended sales price of a grid-scale energy storage system. In the work of Gregory et al.[16] the sales price of organic active materials was estimated by analyzing the production process based on anthraquinones. They found a strong connection between the annual production margin to the price of the synthesized molecule. With a low annual volume of 200 MWh/y they predict an AQDS sells price of 9 $ kg^{-1} while a capacity of 10 GWh/y would lead to a price of 3.48 $ kg^{-1}. Since determining the projected cost for every tailored molecule considered in this study is only vaguely possible, we use the low-margin price of 9 $ kg^{-1} to discuss the present case. Assuming a reasonable high material demand on the market in the Future Case scenario, we apply 3.48 $ kg^{-1} as price for organic active materials. Additionally, given that the predicted values are associated with uncertainty, only future research and development can unveil more precise values. Moreover, it should be noted that organic active materials can be tailored and thus a wide range of prices can be expected as a result of the variety of synthetic routines. Therefore, our calculation tool can be updated if more recent or specific prices are available in future (https://github.com/Domeml94/ReFlowLab).

To evaluate the commercialized all-vanadium RFBs (VRFB), we use the price C_{V2O5} of 20.52 $ kg^{-1} (20.00 € kg^{-1}, converted to dollars at the exchange rate on August 12th in 2022) for vanadium pentoxide (V_2O_5) suggested by Minke et al.[5]. Vanadium pentoxide is often discussed in literature as precursor material for large-scale production of vanadium electrolytes. The material is dissolved in aqueous solution containing sulfuric acid and electrochemically reduced to an equimolar V^{3+}/VO^{2+} electrolyte afterwards.[17] As the vanadium raw material price is known to be highly fluctuating over time, this work also considers the historical price development of the last ten years. During the selected time period the minimum vanadium price was 5.51 $ kg^{-1} in 2015 whereas the highest raw material price of 63.49 $ kg^{-1} was reached during a short season in 2018.[18]

Our calculations are based on a 1.6 M vanadium electrolyte with a 4 M sulfuric acid, reflecting commercially available solutions.[17,19] Only half the amount of precursor material is needed to reach the predefined vanadium concentration, since per molecule V_2O_5 two vanadium ions are received. As the calculation tool has no option of two different input concentrations for either the starting material

or the finished electrolyte solution, we incorporate the factor of ½ by applying half of the vanadium pentoxide molar mass (90.94 g mol^{-1}) in the calculations for the VRFB systems. Additional parameters necessary for the calculations of VRFBs are selected for the actual vanadium species located at the negolyte or posolyte, respectively.

Further parameters necessary to calculate $C_{\text{electrolyte}}$ are retrieved from literature and listed inTable S4. We assume a C_{add} price contribution proposed by Darling et al.[1] that excludes installation costs as those costs are highly variable and specific to the individual project.

Table S4: List of fixed parameter values necessary for the model price calculations in our *study.*

Variable	Symbol	Value	Ref.
**Discharge time**	t_d	4 h	
**System discharge efficiency**	$\varepsilon_{\text{sys, d}}$	0.94	[1]
**Round-trip coulombic efficiency**	$\varepsilon_{\text{q, rt}}$	0.97	[1]
**Allowable state-of-charge range, AqORFB**	χ	0.80	[1]
**Allowable state-of-charge range, VRFB**	χ_{VRFB}	0.80	[1]
**Additional price contributions**	C_{add}	87.5 \$ kW^{-1}	[2]

Maintenance costs:

When developing tailored organic molecules for the application as active materials in RFBs the cycling stability significantly determines whether an individual molecule is viable or not. Molecules with high degradation rates need to be exchanged more frequently over the overall lifetime of an RFB plant, which yields additional cost contributions. Besides the costs that are governed by the active material, the components of the battery stack must also be replaced after a certain time due to wear. The aforementioned points are aggregated into the total cost of plant maintenance shown in equation (S37).

$$C_{\text{maintenance}} = C_{\text{NPV, replacement}} + C_{\text{NPV, stack}} \tag{S37}$$

In the work by Brushett et al.[3] the TE-model of the Dmello working group was further extended. With an additional parameter for the expected costs of the electrolyte exchange, the TE-model is able to display a full review about the cost contributions of a fully organic RFB. Brushett et al.[3] are using degradation rates obtained by cell cycling in lab-scaled RFB setups converted into an annual electrolyte replacement fraction *f*. With a standard discounting of the future payments with an interest rate *r* the present value of electrolyte exchange costs whose payment is due in the future can

be determined. While this calculation assumes a yearly exchange of degraded electrolyte, we extended the formula by considering a degradation threshold for maintenance as well as accounting for possible replacement costs. Reference values for f used for these calculations are listed in Table S8. We are utilizing a target operational lifetime n of 20 years.[3,5] In practice, there is no need for an annual electrolyte replacement. Instead, a certain capacity threshold T is selected depending on the considered application up to which the battery system is still operable. Formula (S38) takes the discount into account for the time in which this threshold is reached. The maintenance fraction F represents the individual maintenance strategy in which portions or the entire electrolyte can be replaced. In our calculations we assume an exchange of the entire electrolyte volume to prevent a possible long-term effect by side reactions of degradation products. With the aforementioned, the overall maintenance costs for electrolyte exchange are summed up in equation (S38).

$$C_{\mathrm{NPV,\,replacement}} = C_{\mathrm{replacement}} \cdot \frac{1}{(1+r)^n} + (C_{\mathrm{replacement}} + C_{\mathrm{electrolyte}} \cdot F) \cdot \left(\sum_{t=1}^{n_{\mathrm{repl}} \cdot t > n} \frac{1}{(1+r)^{n_{\mathrm{repl}} \cdot (t-1)}} \right) \quad \text{(S38)}$$

With: $C_{\mathrm{NPV,\,replacement}}$ = net present value of future costs for electrolyte replacement ($\$\ \mathrm{kWh}^{-1}$)

T = relative capacity threshold for maintenance

$n_{\mathrm{repl}} = \frac{1-T}{f}$ = Electrolyte operation time

$C_{\mathrm{replacement}} = (C_{\mathrm{replacement}}{}^{\mathrm{C}} + C_{\mathrm{replacement}}{}^{\mathrm{A}})$ = replacement cost ($\$\ \mathrm{kWh}^{-1}$)

F = maintenance fraction

The actual replacement of the electrolyte leads to further costs. Most common RFB solvents are based on acids or bases whose disposal leads to additional costs for AqORFBs. The replacement cost per electrolyte mass is determined by the sum of recycling costs as well as waste disposal costs and further maintenance efforts. Replacement costs of AORFBs are not included for evaluation of maintenance costs in this work due to a lack of data and must be revised in future calculations. In the particular case of vanadium RFBs we consider a negative replacement cost value to account for possible recycling earnings. A reuse of already cycled vanadium electrolytes is conceivable, after undergoing some reconditioning process.[20] We assume in the Future Case scenario a residual value of the recyclable electrolyte that equals the net present value of the investment costs.

$$C_{\mathrm{replacement}}{}^{\mathrm{hc}} = \frac{\dfrac{C_{\mathrm{repl.\,el}}{}^{\mathrm{hc}} \cdot s^{\mathrm{hc}} \cdot M^{\mathrm{hc}}}{L^{\mathrm{hc}} \cdot \chi^{\mathrm{hc}} \cdot z_{\mathrm{e}}{}^{\mathrm{hc}}}}{F \cdot U_{\mathrm{d}} \cdot \varepsilon_{\mathrm{sys,\,d}} \cdot \varepsilon_{\mathrm{q,\,rt}}} \quad \text{(S39)}$$

With: $C_{\text{replacement}}{}^{\text{hc}}$ = replacement costs for electrolyte on half cell hc ($ kWh^{-1})

$C_{\text{repl. el}}{}^{\text{hc}}$ = replacement costs per mass electrolyte for electrolyte on half cell hc ($/kg)

L^{hc} = solubility active material (in kg active material per kg electrolyte)

Besides the electrolyte degradation, the battery stack components exhibit wear during long-term operation. The stack replacement costs contain the costs for cell components, pumps, and the maintenance effort. We assume the same stack replacement frequency independently of the active material due to lack of data. Therefore, we use a stack replacement after 10 years.[5] In this procedure not only the stack components are exchanged but also peripherals like pumps and power inverter. We are using the sum of the total specific stack costs $\frac{C_{\text{stack, system}}}{P_{\text{d}}}$, inverter costs ($C_{\text{inverter}}$ = 103 $ kW^{-1} [5]) and costs for pumps ($C_{\text{pump per item}}$ = 205 $ per item[5]), each discounted for the stack operational lifetime.

$$C_{\text{repl. stack}} = \frac{C_{\text{stack, system}}}{P_{\text{d}}} + C_{\text{inverter}} + C_{\text{pump}} \tag{S40}$$

$$C_{\text{NPV, stack}} = \frac{C_{\text{repl. stack}}}{t_{\text{d}}} \cdot \frac{1}{(1+r)^{n_{\text{repl. stack}}}} \tag{S41}$$

With: $C_{\text{repl. stack}}$ = Stack replacement costs ($ / kW)

$n_{\text{repl.stack}}$ = Stack operational lifetime

r = interest rate

We are considering an interest rate of 4%. With an inflation of 2% taken into account we are using a net discount rate of 2% in our calculations.[21]

Table S5: Constants that are considered in net present value calculation of plant maintenance costs.

Variable	Symbol	Value	Ref.
**Discount rate**	r	0.02	[21]
**Operational lifetime / y**	n	20	[5]
**Stack operational lifetime / y**	$n_{\text{repl. stack}}$	10	[5]
**Threshold**	T	0.8	

Table S8 lists all input parameters that are used for the cost calculations of the individual cited active materials. In the herein presented study we focus on aqueous systems due to a higher technological development state and thus a larger amount of available data regarding performance and component information.[6] Instead of symmetric cell cycling like F. Brushett, M. Aziz and K. Rodby proposed for capacity fade values, we made use of available full cell measurement results. Besides the fact that this

approach increases the number of available data extensively, the exclusion by the symmetric cell cycling technique of active material crossover through the semipermeable membrane is far from state-of-the-art application conditions. Most listed capacity fade values in literature are normalized to the number of cycles in which the degradation was measured. We use for each cited system the noted current density, electrode surface area as well as the active material concentration to calculate the theoretical cycle time. With this parameter we were able to standardize the degradation rates to a time region instead of the cycle number.

The costs for individual solvents and salts refer to industrial grade in metric tons scale. Table S9 lists mass specific costs for various salts that are used in the calculations for the herein presented study.

Ideal counter electrode:

For a better comparison of the individual selected organic molecules, we calculate the economic efficiency in reference to a previously specified ideal half cell rather than full cell combinations. The benchmarking parameters for the standard half cell are listed in Table S6. Furthermore, on the half-cell side of the ideal counter electrode no activation overpotential is taken into account. We assume the same material type on both half-cell sides. Due to this assumption, we use the same active material cost for the ideal counter electrode that we apply for the specified active material on the working electrode.

Table S6: Benchmark values of chemical properties for calculations with an ideal aqueous half-cell.

Variable	Symbol	Value	Ref.
**Molecular weight**	M	100 g mol^{-1}	[1]
**Active material solubility**	L_{active}	0.2 kg kg^{-1} (for 2 mol L^{-1})	[2]

We choose the maximum reversible potential of the ideal half cell active material as the electrochemical stability window of the individual solvent. To categorize the selected molecule in negolyte or posolyte we use the maximum cell voltage with one of the two possible water splitting reactions.

Table S7: Potential limits for various solvents at electrolysis with carbon electrodes and mass specific costs of the solvents.

Solvent	$E_{oxidation}$ / V	$E_{reduction}$ / V	$C_{solvent}$ / \$ kg^{-1}

**Water, pH 0, carbon-based electrode**	1.6 vs NHE [22]	-0.6 vs NHE [22]	0.1 [2]

As the stability potential window of water depends on the proton activity of the electrolyte, we are using the following equations to determine the potential values for both reactions in dependency of the pH value:

$$\text{Positive electrode: } E_\text{P} \approx -0.6\ \text{V} + \frac{0.06\ \text{V}}{2} \cdot \log\left(c_{\text{H}_3\text{O}^+}{}^2\right) = -0.6\ \text{V} - 0.06\ \text{V} \cdot pH \quad \text{(S42)}$$

$$\text{Negative electrode: } E_\text{N} \approx 1.6\ \text{V} + \frac{0.06\ \text{V}}{2} \cdot \log\left(c_{\text{H}_3\text{O}^+}{}^2\right) = 1.6\ \text{V} - 0.06\ \text{V} \cdot pH \quad \text{(S43)}$$

2. Supplementary Note: Input values

Table S8: Overview of the main input values used for the cost calculations: various RFB systems form literature that were successfully electrochemically cycled in lab-scale RFB systems.

Active material	Structural formula	Formula	M / g mol^{-1}	n_e / n_H	*Salt*	L / mol L^{-1}	E / V	k_0 / cm s^{-1} (α)<br>D / cm^2 s^{-1}<br>(Ox./Red.: oxidized/reduced molecule)	*Fade rate* / % d^{-1}	f / y^{-1}
**Inorganic salts:**										
**VRFB**		V^{3+} // $VOSO_4$*<br>(VO^{2+})	181.878 *<br>(hydrate)<br>(V_2O_5)	1 / 0<br>// 1 / 2	4M<br>H_2SO_4	1.6 [5]	-0.25 vs NHE [22] // 1.00 vs NHE [22]	(1M H_2SO_4)<br>Ox: 4 · 10^{-3} cm s^{-1} (0.5) [23]<br>Ox: 2.4 · 10^{-6} cm^2 s^{-1} [24] //<br>Ox: 3.0 · 10^{-7} cm s^{-1} (0.42) [23] Ox: 3 · 10^{-6} cm^2 s^{-1} [25]		0 [5]
**$(NH_4)_4[FeCN_6]$****		$C_6H_{16}FeN_{10}$	284.11	1 / 0	1M<br>NH_4Cl	[1M NH_4Cl]<br>1.30 [26]	0.39 vs NHE [26]	(1M NH_4Cl)<br>Ox:<br>1.82 · 10^{-1} cm s^{-1} (N/A)<br>6.31 · 10^{-6} cm^2 s^{-1} [26]	0.0168%/d<br>(0.0007%/h) [26]	0.0613<br>2 [26]
**Quinones:**										
**BQDS (also referred as DHBDS)**<br>(1,2-dihydroxybenzene-3,5-disulfonic acid)	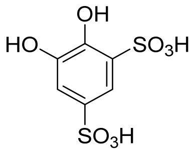	$C_6H_6O_8S_2$	270.226	2 / 2	1M<br>H_2SO_4	4 [acid form] [27]	[1mM DHBDS in 1M H_2SO_4]<br>0.87 V vs NHE [28]	(1mM DHBDS in 1M H_2SO_4)<br>Red: 1.55 · 10^{-4} cm s^{-1} (0.582)<br>3.80 · 10^{-6} cm^2 s^{-1} [28]	9%/d<br>(0.833%/cycle)<br>[28]	32.9<br>[28]

Active material	Structural formula	Formula	*M* / g mol^{-1}	n_e / n_H	*Salt*	*L* / mol L^{-1}	*E* / V	k_0 / cm s^{-1} (α)<br>*D* / cm^2 s^{-1}<br>(Ox./Red.: oxidized/reduced molecule)	*Fade rate* / % d^{-1}	*f* / y^{-1}
**DHDMBS**<br>(3,6-dihydroxy-2,4-dimethylbenzenesulfonic acid)	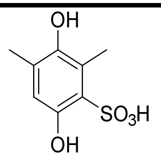	$C_8H_{10}O_5S$	218.223	2 / 2	1 M H_2SO_4	[1M H_2SO_4]<br>~ 2M [29]	[1mM DHDMBS in 1M H_2SO_4]<br>0.85 V vs NHE [30]	(1mM DHDMBS in 1M H_2SO_4)<br>Red: 1.30 · 10^{-4} cm s^{-1} (N/A)<br>4.12 · 10^{-6} cm^2 s^{-1} [29]	0.28%/d<br>(0.05%/cycle)<br>[29]	1.02<br>[29]
**DHBQ**<br>(2,5-dihydroxy-1,4-benzoquinone)	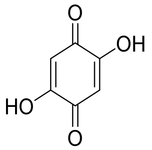	$C_6H_4O_4$	140.094	2 / 0<br>(pH > 10)	2 M KOH	(1 M KOH)<br>> 4.31M [31]	[1mM DHBQ + 1M NaCl + KOH, pH 14]<br>-0.72 V vs SHE [31]	(1mM DHBQ in 1M KOH)<br>Ox: 2.12 · 10^{-3} cm s^{-1} (0.33)<br>5.66 · 10^{-6} cm^2 s^{-1} [31]	8.95%/d<br>(0.24%/cycle)<br>[31]	32.68<br>[31]
**2,7-AQDS**<br>(anthraquinone-2,7-disulfonic acid)	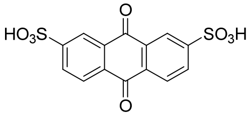	$C_{14}H_8O_8S_2$	368.33	2 / 2	1M H_2SO_4	[H_2SO_4]<br>> 1M [32]	[1mM AQDS in 1M H_2SO_4, pH = 0]<br>0.213 V vs SHE [33]	(1mM AQDS in 1M H_2SO_4)<br>Ox: 7.2 · 10^{-3} cm s^{-1} (0.474)<br>3.8 · 10^{-6} cm^2 s^{-1} [33]	0.54%/d<br>(0.16%/cycle)<br>[34]	1.96<br>[34]
**ARS**<br>(3,4-Dihydroxy-9,10-anthraquinone-2-sulfonic acid)	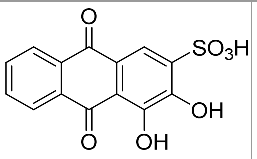	$C_{14}H_8O_7S$	320.271	2 / 2	1M H_2SO_4	[pH 1]<br>0.64M [35]	[1mM ARS in 1M H_2SO_4]<br>0.082 V vs SHE [35]	(1mM ARS in 1M H_2SO_4)<br>Ox: 3.6 · 10^{-3} cm s^{-1} (N/A)<br>2.14 · 10^{-6} cm^2 s^{-1} [35]	16%/d [36]	58.40<br>[36]

Active material	Structural formula	Formula	*M* / g mol^{-1}	n_e / n_H	*Salt*	*L* / mol L^{-1}	*E* / V	k_0 / cm s^{-1} (α)<br>*D* / cm^2 s^{-1}<br>(Ox./Red.: oxidized/reduced molecule)	*Fade rate* / % d^{-1}	*f* / y^{-1}
**AQS**<br>(Anthraquinone-2-sulfonic acid)	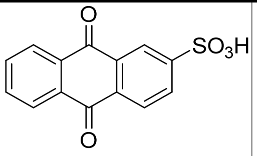	$C_{14}H_8O_5S$	288.273	2 / 2	2M H_2SO_4	[2M H_2SO_4]<br>> 1M [32]	[1mM AQS in 1M H_2SO_4]<br>0.187 V vs SHE [32]	(1mM AQS in 1M H_2SO_4)<br>Ox:<br>$2.25 \cdot 10^{-4}$ cm s^{-1} (0.677)<br>$3.71 \cdot 10^{-6}$ cm^2 s^{-1} [28]	19%/d [32]	69.35 [32]
**DHAQ**<br>(2,6-dihydroxyanthraquinone)	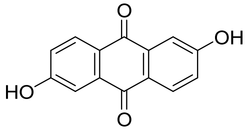	$C_{14}H_8O_4$	240.214	2 / 0 (pH > 12)	1M KOH	[1M KOH]<br>> 0.6M [37]	(1mM 2,6-DHAQ in 1M KOH, pH > 11.7)<br>-0.684 V vs SHE [37]	1mM 2,6-DHAQ in 1M KOH)<br>(Computation):<br>Ox:<br>$7 \cdot 10^{-3}$ cm s^{-1} (0.5)<br>$4.8 \cdot 10^{-6}$ cm^2 s^{-1} [37]	5.60%/d (0.1%/cycle) [37]	20.43 [37]
**DBEAQ**<br>(4,4'-((9,10-anthraquinone-2,6-diyl)dioxy)dibutyrate)	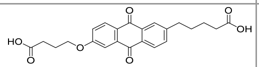	$C_{23}H_{22}O_7$	410.422	2 / 0	1M KOH	[pH 14]<br>1.1M [38]	[5mM 2,6-DBEAQ in 1M KOH]<br>-0.680 V vs SHE [38]	(5mM 2,6-DBEAQ in 1M KOH)<br>(Computation):<br>Ox:<br>$7 \cdot 10^{-3}$ cm s^{-1} (0.5)<br>Ox:<br>$1.58 \cdot 10^{-6}$ cm^2 s^{-1} [38]	< 0.05%/d (0.001%/cycle) [38]	0.18 [38]
**DPivOHAQ**<br>(3,'0-(9,10-anthraquinone-diyl)bis(3-methylbutanoic acid))	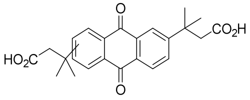	$C_{24}H_{24}O_6$	408.450	2 / 0 (pH > 11)	0.01 M KOH	[pH 12]<br>0.74M [39]	[pH > 11]<br>-0.48 V vs SHE[39]	5mM DPivOHAQ in 1M KCl (pH 12)<br>Ox:	0.014%/d (0.00031%/cycle) [full cell cycling] [39]	0.051 [39]

Active material	Structural formula	Formula	M / g mol^{-1}	n_e / n_H	*Salt*	L / mol L^{-1}	E / V	k_0 / cm s^{-1} (α)<br>D / cm^2 s^{-1}<br>(Ox./Red.: oxidized/reduced molecule)	*Fade rate* / % d^{-1}	f / y^{-1}
								$2.48 \cdot 10^{-3}$ cm s^{-1} (0.49)<br>$2.39 \cdot 10^{-6}$ cm^2 s^{-1} [39]		
**DBAQ**<br>(4,4'-(9,10-anthraquinone-diyl)dibutanoic acid)	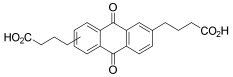	$C_{22}H_{20}O_6$	380.396	2 / 0	0.01 M KOH	[pH 12]<br>1.0M [39]	[pH 12]<br>-0.47 V vs SHE<br>[39]	5mM DBAQ in 1M KCl (pH 12)<br><br>Ox:<br>$2.87 \cdot 10^{-3}$ cm s^{-1} (0.50)<br>$2.54 \cdot 10^{-6}$ cm^2 s^{-1} [39]	0.0084%/d [39]	0.031<br>[39]
**2,3-HCNQ**<br>(2-Hydroxy-3-Carboxy-1,4-Naphthoquinone)	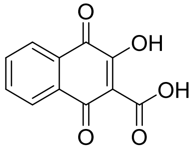	$C_{11}H_6O_5$	218.164	2 / 0	1M KOH	[1.0M KOH]<br>1.2M	(2mM 2,3-HCNQ in 1.0M KOH, pH 14)<br>-0.52 V vs SHE<br>[40]	(2mM 2,3-HCNQ in 1.0M KOH)<br><br>Ox:<br>$2.07 \cdot 10^{-3}$ cm s^{-1} (0.71)<br>$3.44 \cdot 10^{-6}$ cm^2 s^{-1} [40]	3.4%/d [40]	12.41<br>[40]
**2,6-DPPEAQ** (((9,10-dioxo-9,10-dihydroanthracene-2,6-diyl)bis(oxy))bis(propane-3,1-diyl))bis(phosphonic acid)	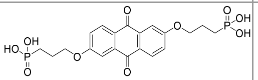	$C_{20}H_{22}O_{10}P_2$	484.33352	2 / 2<br>(pH < 9)	1.0 10^{-4} M KOH	[pH 9]<br>0.75M<br>[41]	[pH 9]<br>-0.47 V vs SHE<br>[41]	(5mM 2,6-DPPEAQ in 1M KOH)<br><br>Ox: (DBAQ)<br>$2.87 \cdot 10^{-3}$ cm s^{-1} (0.50) [39]<br>Ox:<br>$1.37 \cdot 10^{-6}$ cm^2 s^{-1}<br>[41]	0.014%/d<br>(0.00036%/cycle)<br>[41]	0.05<br>[41]

**Active material**	**Structural formula**	**Formula**	***M* / g mol^{-1}**	***n*$_e$ / *n*$_H$**	***Salt***	***L* / mol L^{-1}**	***E* / V**	***k$_0$ / cm s^{-1} (α)*** ***D* / cm^2 s^{-1}** **(Ox./Red.: oxidized/reduced molecule)**	***Fade rate* / % d^{-1}**	***f* / y^{-1}**
**TMHQ** (Tetramorpholinohydroquinone)	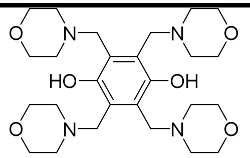	$C_{26}H_{42}N_4O_6$	506.644	2 / 2 (TMQ$^+$, 5.20 < pH < 8.57)	1M NH_4Cl	2M [H_2O, TMHQ^{4+}] [42]	[1M HCl, pH 0] 0.89 V vs SHE [42]	[TMHQ^{1+}] Red: 6.69 · 10^{-4} cm s^{-1} (0.76) 6.58 · 10^{-6} cm^2 s^{-1} [42]	70%/d (0.67%/cycle) [36]	255.5 [36]
**2-2PEAQ** (2-2-propionate ether anthraquinone)	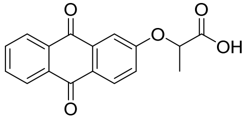	$C_{17}H_{12}O_5$	296.28	2 / 0	1 M KOH	1.0 M (pH 14) [43]	[5 mM 2-2PEAQ in 1M KOH] -0.477 V vs SHE [43]	[5 mM 2-2PEAQ in 1M KOH] Ox: 1.75 · 10^{-3} cm s^{-1} (N/A) 2.23 · 10^{-6} cm^2 s^{-1} [43]	0.009%/d [43]	0.0329 [43]
**Viologens:**										
**MVi** (Methyl viologen; 4,4-dimethyl bipyridinium dichloride)	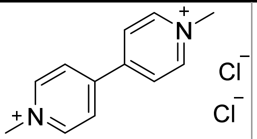	$C_{12}H_{14}Cl_2N_2$	257.158	1 / 0	1 M NaCl	[pH 7] 3.0M [44]	[4mM MVi in 0.5M NaCl, pH 7] -0.45 V vs NHE [44]	(1mM MVi in 0.5 NaCl) Ox: 2.8 · 10^{-4} cm s^{-1} (N/A) 2.57 · 10^{-5} cm^2 s^{-1} [44]	19.70%/d (0.1/cycle) [44]	71.91 [44]
**BTMAP-Vi** (Bis(3-trimethylammonio)propyl viologen tetrachloride)	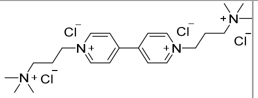	$C_{22}H_{38}Cl_4N_4$	500.374	(2x1) / 0 [45]	1 M NaCl ***	2.0M [H_2O, pH 7] [46]	[1.0mM BTMAP-Vi in 0.5M NaCl] -0.72 V vs NHE [45]	(1mM BTMAP-Vi in 0.50M NaCl) Ox: 2.2 · 10^{-2} cm s^{-1} (0.47) 3.3 · 10^{-6} cm^2 s^{-1} [46]	0.10%/d (0.0057%/cycle) [46]	0.37 [46]

Active material	Structural formula	Formula	*M* / g mol^{-1}	n_e / n_H	*Salt*	*L* / mol L^{-1}	*E* / V	k_0 / cm s^{-1} (α)<br>*D* / cm^2 s^{-1}<br>(Ox./Red.: oxidized/reduced molecule)	*Fade rate* / % d^{-1}	*f* / y^{-1}
**MTMAP-Vi**<br>(1-methyl-1'-[3-(trimethylammonio)propyl]-4,4'-bipyridinium<br>Trichloride)	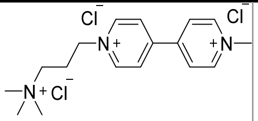	$C_{17}H_{26}Cl_3N_3$	378.766	2 / 0	2 M NaCl	[2M NaCl, pH 7] 1.4M [45]	[4mM MTMAP-Vi in 0.5M NaCl]<br><br>-0.78 V vs NHE [45]	(1.0mM MTMAP-Vi in 0.5M NaCl)<br><br>Ox:<br>>3.60 · 10^{-1} cm s^{-1} (N/A)<br>5.29 · 10^{-6} cm^2 s^{-1}<br>[45]	8.06 %/d<br>(0.18%/cycle)<br>[45]	29.42
**BTMAP-TTZ-Vi**<br>(4,4'-(thiazolo[5,4-d]thiazole-2,5-diyl)bis(1-(3-(trimethylammonio)propyl)pyridine-1-ium) tetrachloride)	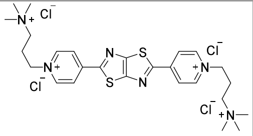	$C_{26}H_{38}Cl_4N_6S_2$	640.552	2 / 0	2 M NaCl	[2M NaCl, pH 7]<br>1.1M [47]	[4mM BTMAP-TTZ-Vi in 0.5M NaCl]<br><br>Avg.: -0.44 V vs NHE [47]	(4mM BTMAP-TTZ-Vi in 0.5M NaCl)<br><br>Ox:<br>> 2.8 · 10^{-1} cm s^{-1} (N/A)<br>3.15 · 10^{-6} cm^2 s^{-1}<br>[47]	2.24%/d<br>(0.03%/cycle)<br>[47]	8.17<br>[47]
**$(SPr)_2$-Vi**<br>(1,1,'-bis(3-sulfonatopropyl)-4,4'-bipyridinium)	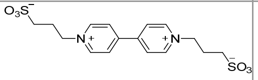	$C_{16}H_{20}N_2O_6S_2$	400.464	1 / 0	2 M KCl	[H_2O, pH 7]<br>2.0M [48]	[4.0mM $(SPr)_2$-Vi in 0.5M KCl]<br><br>-0.43 V vs NHE [48]	(4.0mM $(SPr)_2$-Vi in 0.5M KCl)<br><br>Ox:<br>> 2.8 · 10^{-1} cm s^{-1} (N/A)<br>3.26 · 10^{-6} cm^2 s^{-1} [48]	0.45%/d<br>(0.01%/cycle)<br>[48]	1.63<br>[48]
**TEMPO:**										

Active material	Structural formula	Formula	*M* / g mol^{-1}	n_e / n_H	*Salt*	*L* / mol L^{-1}	*E* / V	k_0 / cm s^{-1} (α)<br>*D* / cm^2 s^{-1}<br>(Ox./Red.: oxidized/reduced molecule)	*Fade rate* / % d^{-1}	*f* / y^{-1}
**4-OH-TEMPO**<br>(4-hydroxy-2,2,6,6-tetramethylpiperidine-1-oxyl)	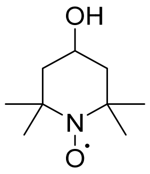	$C_9H_{18}NO_2$	172.248	1 / 0	0.5 M NaCl	[H_2O, pH 7]<br>~ 2.1M<br>[44]	[4mM 4-OH-TEMPO in 0.5M NaCl, pH 7]<br>0.8 V vs NHE<br>[44]	(1mM 4-OH-TEMPO in 0.5 NaCl)<br>Red:<br>$2.6 \cdot 10^{-4}$ cm s^{-1} (N/A)<br>$2.95 \cdot 10^{-5}$ cm^2 s^{-1} [44]	19.70%/d<br>(0.1/cycle) [44]	71.91<br>[44]
**TEMPOSP**<br>(TEMPO-4-sulfate potassium salt)	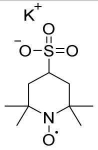	$C_9H_{17}KNO_4S$	274.3963	1 / 0	2 M NH_4Cl	[2M $ZnCl_2$ + 2M NH_4Cl]<br>>1M [49]	[1mM TEMPOSP in 0.05M $ZnCl_2$ + 0.05M NH_4Cl]<br>0.82 V vs SHE<br>[49]	(0.014M TEMPOSP in 0.05M $ZnCl_2$ + 0.05M NH_4Cl)<br>Red:<br>$1.91 \cdot 10^{-3}$ cm s^{-1} (0.68)<br>$2.98 \cdot 10^{-6}$ cm^2 s^{-1} [49]	1.38%/d<br>(0.0058%/cycle)<br>[49]	5.04<br>[49]
**TEMPTMA**<br>(N,N,N-2,2,6,6-heptamethylpiperidinyl-oxy-4-ammonium chloride)	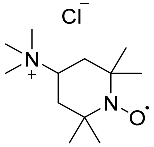	$C_{12}H_{26}ClN_2O$	249.803	1 / 0	0.3 M NaCl	3.2M [0.3M NaCl]<br>[50]	[0.1M NaCl, pH 7]<br>1.00 V vs SHE<br>[50]	(4.6 mM TEMPTMA in 0.1M NaCl)<br>Red:<br>$4.2 \cdot 10^{-3}$ cm s^{-1} (0.58)<br>$4.8 \cdot 10^{-6}$ cm^2 s^{-1} [50]	0.27 %/d<br>(0.026 %/cycle)<br>[36]	0.99<br>[36]
**Aza-aromatics:**										

Active material	Structural formula	Formula	M / g mol^{-1}	n_e / n_H	*Salt*	L / mol L^{-1}	E / V	k_0 / cm s^{-1} (α)<br>D / cm^2 s^{-1}<br>(Ox./Red.: oxidized/reduced molecule)	*Fade rate* / % d^{-1}	f / y^{-1}
**ACA**<br>(Alloxazine-COOH)	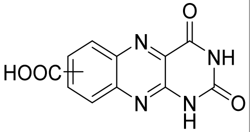	$C_{11}H_6N_4O_4$	258.193	2 / 0	1 M KOH	[1M KOH, pH 14]<br><br>1.0M [51]	[2mM ACA in 1M KOH, pH 14]<br><br>-0.62 V vs SHE [51]	(2mM ACA in 1M KOH)<br><br>Ox:<br>$1.2 \cdot 10^{-5}$ cm s^{-1} (0.47) [51]<br><br>$2.5 \cdot 10^{-5}$ cm^2 s^{-1}[52]	1.2 %/d<br>(0.015 %/cycle) [51]	4.38 [51]
**FMN-Na**<br>(flavin mononucleotide sodium salt)	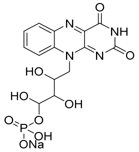	$C_{14}H_{14}N_4NaO_9P$	436.24853	2 / 0<br>(pH > 8.6)	1 M KOH	[1M KOH + 3M nicotinamide]<br><br>1.5M [53]	[10mM FMN-Na in 1M KOH, pH 13]<br><br>-0.53 V vs SHE [53]	(10mM FMN-Na in 1M KOH)<br><br>Ox:<br>$5.3 \cdot 10^{-3}$ cm s^{-1} (0.50)<br>$1.3 \cdot 10^{-6}$ cm^2 s^{-1} [53]	0.47%/d<br>(0.01 %/cycle) [53]	1.70 [53]
**MB**<br>(methylene blue)	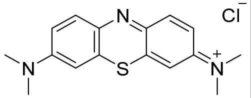	$C_{16}H_{18}ClN_3S$	319.851	2 / 2	3.5M H_2SO_4	[H_2O + acetic acid + H_2SO_4]<br><br>~ 1.8M [54]	[3M H_2SO_4]<br>0.57 V vs NHE [54]	(3.0M H_2SO_4)<br>Ox:<br>$3.20 \cdot 10^{-1}$ cm s^{-1} (N/A)<br>$2.51 \cdot 10^{-6}$ cm^2 s^{-1} [54]	0.76%/d<br>(0.074%/cycle) [54]	2.77 [54]
**Azobenzene:**										

Active material	Structural formula	Formula	*M* / g mol^{-1}	n_e / n_H	*Salt*	*L* / mol L^{-1}	*E* / V	k_0 / cm s^{-1} (α)<br>*D* / cm^2 s^{-1}<br>(Ox./Red.: oxidized/reduced molecule)	*Fade rate* / % d^{-1}	*f* / y^{-1}
**AADA**<br>(4-amino-1,1'-azobenzene-3,4'-disulfonic acid monosodium salt)	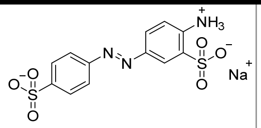	$C_{12}H_{10}N_3NaO_6S_2$	379.33677	2 / 0	2M NaOH	[2M NaOH]<br>2M [55]	[10mM AADA in 2M NaOH]<br>-0.58V vs NHE [55]	(10mM AADA in 2M/4M urea in 2M NaOH)<br>Ox:<br>3.66/3.47 · 10^{-4} cm s^{-1} (N/A)<br>2.49/3.03 · 10^{-6} cm^2 s^{-1} [55]	4.477%/d<br>(0.05%/cycle)<br>[55]	16.34<br>[55]
**Phenazine:**										
**HP**<br>(2-Hydroxyphenazine)	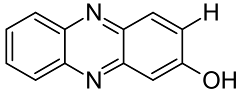	$C_{12}H_8N_2O$	196.20472	2 / 2	1M KOH	[1M KOH]<br>1.70M<br>[56]	[2mM HP in 1M KOH]<br>-0.67 V vs SHE<br>[56]	(2mM HP in 1M KOH)<br>Ox: 1.42 · 10^{-3} cm s^{-1} (0.77)<br>3.79 · 10^{-6} cm^2 s^{-1} [56]	42.31%/d<br>(0.378 %/cycle)<br>[56]	154.44<br>[56]
**AHP**<br>(2-amino-3-hydroxyphenazine)	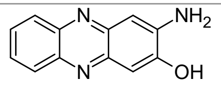	$C_{12}H_9N_3O$	211.21936	2 / 2	1M KOH	[1M KOH]<br>0.43M<br>[56]	[2mM AHP in 1M KOH]<br>-0.78 V vs SHE<br>[56]	(2mM AHP in 1M KOH)<br>Ox: 1.63 · 10^{-3} cm s^{-1} (0.71)<br>2.85 · 10^{-6} cm^2 s^{-1} [56]	0.69%/d [56]	2.52<br>[56]
**BHPC**<br>(benzo[a]hydroxyphenazine-7/8-carboxylic acid)	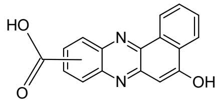	$C_{17}H_{10}N_2O_3$	290.2729	2 / 2	1M KOH	[1M KOH]<br>1.55M<br>[56]	[2mM BHPC in 1M KOH]<br>-0.78 V vs SHE<br>[56]	(2mM BHPC in 1M KOH)<br>Ox: 1.55 · 10^{-3} cm s^{-1} (0.77)<br>3.52 · 10^{-6} cm^2 s^{-1} [56]	0.08%/d [56]	0.29<br>[56]

Active material	Structural formula	Formula	*M* / g mol^{-1}	n_e / n_H	*Salt*	*L* / mol L^{-1}	*E* / V	k_0 / cm s^{-1} (α)<br>*D* / cm^2 s^{-1}<br>(Ox./Red.: oxidized/reduced molecule)	*Fade rate* / % d^{-1}	*f* / y^{-1}
**2,7-DGAP**<br>(2,7-Diglycineamino phenazine)	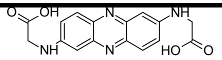	$C_{16}H_{14}N_4O_4$	326.30676	2 / 2	1M KCl, pH 12 (KOH)	[H_2O] 0.886M [57]	[1mM 2,7-DGAP in 1M KCl, pH 12]<br>-0.52 V vs SHE [57]	[1mM 2,7-DGAP in 1M KCl, pH 12]<br>Ox: 1.04 · 10^{-3} cm s^{-1} (0.55)<br>2.87 · 10^{-6} cm^2 s^{-1} [57]	24.71%/d [57]	90.19 [57]
**2,7-DAAP**<br>(2,7-Dialanineamino phenazine)	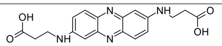	$C_{18}H_{18}N_4O_4$	354.35992	2 / 2	1M KCl, pH 12 (KOH)	[H_2O] 1.002M [57]	[1mM 2,7-DAAP in 1M KCl, pH 12]<br>-0.54 V vs SHE [57]	[1mM 2,7-DAAP in 1M KCl, pH 12]<br>Ox: 8.60 · 10^{-4} cm s^{-1} (0.35)<br>3.39 · 10^{-6} cm^2 s^{-1} [57]	13.88%/d	50.66 [57]
**2,7-DPAP**<br>(phenazine-2,7-diylbis(azanediyl))dipropionic acid)	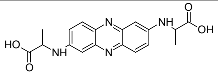	$C_{18}H_{18}N_4O_4$	354.35992	2 / 2	1M KCl, pH 12 (KOH)	[H_2O] 1.047M [57]	[1mM 2,7-DPAP in 1M KCl, pH 12]<br>-0.52 V vs SHE [57]	[1mM 2,7-DPAP in 1M KCl, pH 12]<br>Ox: 1.03 · 10^{-3} cm s^{-1} (0.61)<br>2.87 · 10^{-6} cm^2 s^{-1} [57]	25.75%/d [57]	93.99 [57]
**2,7-DBAP**<br>(2,7-Di-γ-aminobutyric amino phenazine)	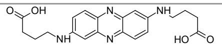	$C_{20}H_{22}N_4O_4$	382.41308	2 / 2	1M KCl, pH 12 (KOH)	[H_2O] 0.996M [57]	[1mM 2,7-DBAP in 1M KCl, pH 12]<br>-0.53 V vs SHE [57]	[1mM 2,7-DBAP in 1M KCl, pH 12]<br>Ox: 1.56 · 10^{-3} cm s^{-1} (0.42)<br>2.38 · 10^{-6} cm^2 s^{-1} [57]	36.95%/d [57]	134.87 [57]

Active material	Structural formula	Formula	*M* / g mol^{-1}	n_e / n_H	*Salt*	*L* / mol L^{-1}	*E* / V	k_0 / cm s^{-1} (α)<br>*D* / cm^2 s^{-1}<br>(Ox./Red.: oxidized/reduced molecule)	*Fade rate* / % d^{-1}	*f* / y^{-1}
**1,8-DGAP**<br>(1,8-Diglycineamino phenazine)	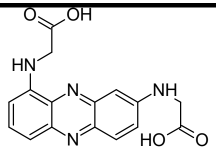	$C_{16}H_{14}N_4O_4$	326.30676	2 / 2	1M KCl, pH 12 (KOH)	[H_2O] 0.924M [57]	[1mM 1,8-DGAP in 1M KCl, pH 12]<br>-0.57 V vs SHE [57]	[1mM 1,8-DGAP in 1M KCl, pH 12]<br>Ox: 6.46 · 10^{-4} cm s^{-1} (0.34)<br>3.71 · 10^{-6} cm^2 s^{-1} [57]	6.24%/d [57]	22.78 [57]
**1,6-DPAP**<br>(3,3'-(phenazine-1,6-diylbis(azanediyl))dipropionic acid)	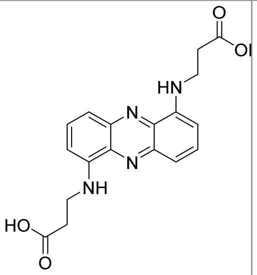	$C_{18}H_{18}N_4O_4$	354.37	2 / 2	1M KCl, pH 8 (KOH)	[H_2O] 1.005 [57]	[1mM 1,6-DPAP in 1M KCl, pH 12]<br>-0.56 V vs SHE [57]	[1mM 1,6-DPAP in 1M KCl, pH 12]<br>Ox: 5.13 · 10^{-4} cm s^{-1} (0.53)<br>4.08 · 10^{-6} cm^2 s^{-1} [57]	0.0015 %/d [57]	0.0054 75 [57]
**1,6-PFP**<br>(3,3'-(phenazine-1,6-diyl)dipropionic acid)	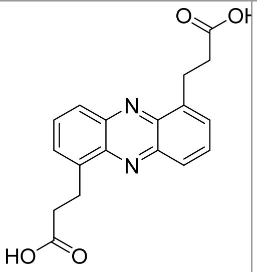	$C_{18}H_{16}N_2O_4$	324.34	2 / 2	pH 12 (KOH)	[H_2O, pH 12] 1.02 [58]	[1mM 1,6-PFP in 1M KOH, pH 14]<br>-0.560 V vs SHE [58]	[1M KOH, pH 14]<br>Ox: 3.08 · 10^{-5} cm s^{-1} (0.52)<br>2.90 · 10^{-6} cm^2 s^{-1} [58]	0.00469 %/d [58]	0.0171 [58]
**1,8-PFP**<br>(3,3'-(phenazine-1,8-diyl)dipropionic acid)	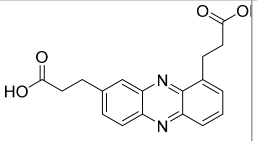	$C_{18}H_{16}N_2O_4$	324.34	2 / 2	pH 12 (KOH)	[H_2O, pH 12] 1.09 [58]	[1mM 1,8-PFP in 1M KOH]<br>-0.588 V vs SHE [58]	[1M KOH, pH 14]<br>Ox: 1.92 · 10^{-4} cm s^{-1} (0.45)<br>2.55 · 10^{-6} cm^2 s^{-1} [58]	0.00465 %/d [58]	0.0170 [58]

Active material	Structural formula	Formula	*M* / g mol^{-1}	n_e / n_H	*Salt*	*L* / mol L^{-1}	*E* / V	k_0 / cm s^{-1} (α)<br>*D* / cm^2 s^{-1}<br>(Ox./Red.: oxidized/reduced molecule)	*Fade rate* / % d^{-1}	*f* / y^{-1}
**2,7-PFP**<br>(3,3'-(phenazine-2,7-diyl)dipropionic Acid)	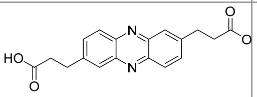	$C_{18}H_{16}N_2O_4$	324.34	2 / 2	pH 12 (KOH)	[H_2O, pH 12]<br>0.79 [58]	[1mM 1,8-PFP in 1M KOH]<br>-0.611 V vs SHE [58]	[1M KOH, pH 14]<br>Ox: 6.15 · 10^{-4} cm s^{-1} (0.58)<br>2.70 · 10^{-6} cm^2 s^{-1} [58]	0.0401 %/d [58]	0.1464 [58]

*: *C*(V_2O_5) = 20.52 $ kg^{-1}; 63.49 $ kg^{-1}; 5.51 $ kg^{-1}; Assumption: vanadium electrolyte operational lifetime of 20 years[5]; We apply with 90.939 g mol^{-1} half of the 181.878 g mol^{-1} to account for the stoichiometric number of vanadium per molecule V_2O_5

**: *C*($(NH_4)_4[FeCN_6]$) = 1.28 $/kg [26]

***: The literature source does not mention the use of salt additive. We used 1M NaCl as conductive salt addition for the evaluation to be closer to realistic application conditions.

Table S9: Mass specific costs for different salts considered for electrolyte cost calculation.

Salt	C_{salt} / $ kg^{-1}	*Ref.*
**H_2SO_4**	0.170	https://www.echemi.com/produce/pr2204301195-sulfuric-acid-99-liquid-industrial-flask.html, 13.08.22
**KCl**	0.092	https://www.echemi.com/produce/pr2206041069-potassium-chloride-potassium-sulphate-99-potassium-chloride.html, 13.08.22
**KOH**	0.700	https://www.echemi.com/produce/pr2206041076-potassium-hydroxide-99-99-flakes-koh.html, 13.08.22

**NaCl**	0.080	https://www.echemi.com/produce/pr2206225026-nacl-sodium-chloride-snow-melting-99-white-powder.html, 13.08.22
**NaOH**	0.420	https://www.echemi.com/produce/pr2204301002-caustic-soda-flakes-pearls-99-99-non-white-solid-naoh-caustic-soda.html, 13.08.22
**NH_4Cl**	0.010	https://www.echemi.com/produce/pr2204211945-cas-no-12125-02-9-fcc-usp-ep-bp-nh4cl-white-crystal-low-price-high-purity-ammonium-chloride.html, 13.08.22

3. Supplementary Note: Results

Working point 1 - spec. power 0.1 W cm^{-2}

Table S10: Calculation results: Future Case – $C_{a,\ org.}$ = 3.48 \$/kg, size selective membrane, vanadium residual value. (WP1 – spec. power 0.1 W cm^{-2})

Active material	$U_{d,\ cell}$ / V	I_d / A cm^{-2}	n_{stack}	$C_{electrolyte}$ / \$ kWh^{-1}	$C_{maintenance}$ / \$ kWh^{-1}	C_{power} / \$ kW^{-1}	$C_{Capital}$ / \$ kWh^{-1}
**Inorganic salts:**							
**VRFB (20.52 \$ kg^{-1})**	0.74	0.14	442.78	293.13	-89.54	1,299.47	**528.46**
**VRFB (63.49 \$ kg^{-1})**	0.74	0.14	442.78	832.69	-399.54	1,299.47	**758.01**
**VRFB (5.51 \$ kg^{-1})**	0.74	0.14	442.78	104.66	18.72	1,299.47	**448.24**
**Quinones:**							
**BQDS**	1.42	0.07	425.94	42.99	116,843.4	980.41	**117,131.49**
**DHDMBS**	1.39	0.07	438.64	45.05	3,859.65	998.23	**4,154.26**
**DHBQ**	1.48	0.07	427.71	34.62	93,491.56	982.88	**93,771.9**

Active material	$U_{d, cell}$ / V	I_d / A cm^{-2}	n_{stack}	$C_{electrolyte}$ / \$ kWh^{-1}	$C_{maintenance}$ / \$ kWh^{-1}	C_{power} / \$ kW^{-1}	$C_{Capital}$ / \$ kWh^{-1}
**2,7-AQDS**	1.35	0.07	442.93	64.93	10,577.84	1,004.26	**10,893.83**
**ARS**	1.5	0.07	442.94	63.02	303,987.66	1,004.26	**304,301.75**
**AQS**	1.37	0.07	439.94	59.32	339,769.11	1,000.06	**340,078.45**
**DHAQ**	1.42	0.07	441.91	66.18	111,711.68	1,002.83	**112,028.56**
**DBEAQ**	1.42	0.07	441.34	63.9	1,018.6	1,002.02	**1,333.01**
**DPivOHAQ**	1.33	0.08	442.66	74.47	379.26	1,003.87	**704.7**
**DBAQ**	1.32	0.08	441.88	66.75	235.69	1,002.78	**553.14**
**2,3-HCNQ**	1.25	0.08	440.6	57.23	58,715.7	1,000.98	**59,023.18**
**2,6-DPPEAQ**	1.5	0.07	441.26	70.15	359.68	1,001.91	**680.31**
**TMHQ**	1.88	0.05	435.32	46.61	983,387.91	993.57	**983,682.91**
**2-2PEAQ**	1.21	0.08	442.68	68.62	241.95	1,003.9	**561.55**
**Viologens:**							
**MVi**	1.57	0.06	437.93	54.41	323,164.46	997.24	**323,468.18**
**BTMAP-Vi**	1.88	0.05	436.59	46.37	1,482.42	995.35	**1,777.64**
**MTMAP-Vi**	1.94	0.05	431.78	42.01	102,113.44	988.6	**102,402.6**
**BTMAP-TTZ-Vi**	1.6	0.06	438.19	68.54	46,306.72	997.6	**46,624.65**

Active material	$U_{d, cell}$ / V	I_d / A cm^{-2}	n_{stack}	$C_{electrolyte}$ / \$ kWh^{-1}	$C_{maintenance}$ / \$ kWh^{-1}	C_{power} / \$ kW^{-1}	$C_{Capital}$ / \$ kWh^{-1}
**$(SPr)_2$-Vi**	1.59	0.06	438.64	72.85	9,875.5	998.23	**10,197.91**
**TEMPO:**							
**4-OH-TEMPO**	1.74	0.06	442.29	42.53	252,613.5	1,003.35	**252,906.87**
**TEMPOSP**	1.79	0.06	440.7	58.87	24,566.96	1,001.13	**24,876.11**
**TEMPTMA**	1.99	0.05	430.17	41.99	3,496.93	986.34	**3,785.5**
**Aza-aromatics:**							
**ACA**	1.24	0.08	434.92	63.98	23,208.66	993.01	**23,520.9**
**FMN-Na**	1.27	0.08	441.42	68.76	9,721.96	1,002.14	**10,041.25**
**MB**	1.1	0.09	438.7	67.56	15,521.03	998.32	**15,838.17**
**Azobenzene:**							
**AADA**	1.3	0.08	436.35	59.55	80,408.15	995.02	**80,716.45**
**Phenazine:**							
**HP**	1.41	0.07	437.56	45.29	577,657.09	996.71	**577,951.56**
**AHP**	1.51	0.07	441.4	71.31	14,909.14	1,002.1	**15,230.97**
**BHPC**	1.52	0.07	435.65	48.38	1,224.28	994.04	**1,521.17**
**2,7-DGAP**	1.37	0.07	441.5	63.4	472,243.86	1,002.25	**472,557.83**

Active material	$U_{d,\ cell}$ / V	I_d / A cm^{-2}	n_{stack}	$C_{electrolyte}$ / \$ kWh^{-1}	$C_{maintenance}$ / \$ kWh^{-1}	C_{power} / \$ kW^{-1}	$C_{Capital}$ / \$ kWh^{-1}
**2,7-DAAP**	1.39	0.07	442.51	62.14	259,990.64	1,003.66	**260,303.69**
**2,7-DPAP**	1.37	0.07	438.76	62.15	482,399.4	998.41	**482,711.15**
**2,7-DBAP**	1.38	0.07	439.24	64.25	715,631.26	999.07	**715,945.28**
**1,8-DGAP**	1.41	0.07	438.48	60.71	114,269.14	998.01	**114,579.35**
**1,6-DPAP**	1.4	0.07	440.5	61.37	58.37	1,000.85	**369.95**
**1,6-PFP**	1.21	0.08	439.52	68.46	126.75	999.47	**445.07**
**1,8-PFP**	1.29	0.08	441.45	63.46	121.91	1,002.17	**435.91**
**2,7-PFP**	1.33	0.08	442.53	67.44	855.14	1,003.69	**1,173.5**

Table S11: Calculation results: Present Case – $C_{a, org.}$ = 9 \$/kg, Nafion membrane, no vanadium residual value. (WP1 – spec. power 0.1 W cm^{-2})

Active material	$U_{d, cell}$ / V	I_d / A cm^{-2}	n_{stack}	$C_{electrolyte}$ / \$ kWh^{-1}	$C_{maintenance}$ / \$ kWh^{-1}	C_{power} / \$ kW^{-1}	$C_{Capital}$ / \$ kWh^{-1}
**Inorganic salts:**							
**VRFB**	0.74	0.14	442.78	293.13	58.70	1,299.47	**676.70**
**Quinones:**							
**BQDS**	1.38	0.07	439.01	92.35	250,940.68	1,291.65	**251,355.94**
**DHDMBS**	1.34	0.07	442.14	90.46	7,692.4	1,298.15	**8,107.4**
**DHBQ**	1.44	0.07	439.79	68.98	186,217.3	1,293.28	**186,609.6**
**2,7-AQDS**	1.31	0.08	442.76	128.55	20,883.6	1,299.42	**21,337**
**ARS**	1.46	0.07	442.23	111.97	540,039.86	1,298.32	**540,476.41**
**AQS**	1.33	0.08	440.06	113.29	648,837.92	1,293.82	**649,274.67**
**DHAQ**	1.38	0.07	440.78	113.34	191,271.62	1,295.32	**191,708.79**
**DBEAQ**	1.38	0.07	440.37	128.56	1,990.33	1,294.47	**2,442.5**

Active material	$U_{d, cell}$ / V	I_d / A cm^{-2}	n_{stack}	$C_{electrolyte}$ / \$ kWh^{-1}	$C_{maintenance}$ / \$ kWh^{-1}	C_{power} / \$ kW^{-1}	$C_{Capital}$ / \$ kWh^{-1}
**DPivOHAQ**	1.28	0.08	440.86	144.09	679.08	1,295.5	**1,147.05**
**DBAQ**	1.28	0.08	441.49	133.41	412.73	1,296.79	**870.34**
**2,3-HCNQ**	1.2	0.08	442.22	108.66	111,423.3	1,298.31	**111,856.54**
**2,6-DPPEAQ**	1.46	0.07	440.99	138.19	651.97	1,295.76	**1,114.1**
**TMHQ**	1.85	0.05	442.72	101.37	2,138,709.69	1,299.33	**2,139,135.89**
**2-2PEAQ**	1.15	0.09	441.79	132.49	412.7	1,297.42	**869.54**
**Viologens:**							
**MVi**	1.53	0.07	425.27	121.95	724,188.75	1,263.19	**724,626.49**
**BTMAP-Vi**	1.85	0.06	431.29	100.65	3,149.3	1,275.67	**3,568.87**
**MTMAP-Vi**	1.91	0.05	438.69	85.4	207,537.43	1,291	**207,945.58**
**BTMAP-TTZ-Vi**	1.56	0.06	442.68	146.22	98,725.53	1,299.25	**99,196.56**
**$(SPr)_2$-Vi**	1.55	0.07	438.06	165.93	22,418.12	1,289.7	**22,906.47**
**TEMPO:**							
**4-OH-TEMPO**	1.7	0.06	430.67	88.63	526,346.02	1,274.39	**526,753.25**
**TEMPOSP**	1.76	0.06	442.94	120.22	50,108.39	1,299.79	**50,553.56**
**TEMPTMA**	1.96	0.05	436.76	93.04	7,679	1,287.01	**8,093.79**

**Active material**	$U_{d, cell}$ / V	I_d / A cm^{-2}	n_{stack}	$C_{electrolyte}$ / \$ kWh^{-1}	$C_{maintenance}$ / \$ kWh^{-1}	C_{power} / \$ kW^{-1}	$C_{Capital}$ / \$ kWh^{-1}
**Aza-aromatics:**							
**ACA**	1.19	0.08	440.25	121.12	43,881.71	1,294.23	**44,326.38**
**FMN-Na**	1.22	0.08	442.81	145.36	20,486.86	1,299.52	**20,957.1**
**MB**	1.04	0.1	440.87	141.88	32,532.06	1,295.51	**32,997.81**
**Azobenzene:**							
**AADA**	1.26	0.08	438.24	126.92	171,322.12	1,290.07	**171,771.56**
**Phenazine:**							
**HP**	1.36	0.07	442.25	87.74	1,118,999.61	1,298.38	**1,119,411.95**
**AHP**	1.47	0.07	442.74	112.77	23,541.47	1,299.39	**23,979.09**
**BHPC**	1.48	0.07	438.91	96.48	2,384.5	1,291.44	**2,803.84**
**2,7-DGAP**	1.32	0.08	440.29	121.79	907,049.57	1,294.31	**907,494.94**
**2,7-DAAP**	1.34	0.07	441.73	122.56	512,779.99	1,297.3	**513,226.87**
**2,7-DPAP**	1.32	0.08	439.34	123.39	957,678.21	1,292.35	**958,124.69**
**2,7-DBAP**	1.34	0.08	441.31	127.91	1,424,493.16	1,296.43	**1,424,945.18**
**1,8-DGAP**	1.37	0.07	442.35	117.02	220,198.53	1,298.57	**220,640.19**
**1,6-DPAP**	1.36	0.07	443.09	120.97	58.72	1,300.11	**504.72**

Active material	$U_{d, cell}$ / V	I_d / A cm^{-2}	n_{stack}	$C_{electrolyte}$ / \$ kWh^{-1}	$C_{maintenance}$ / \$ kWh^{-1}	C_{power} / \$ kW^{-1}	$C_{Capital}$ / \$ kWh^{-1}
**1,6-PFP**	1.16	0.09	442.75	135.28	193.97	1,299.4	**654.11**
**1,8-PFP**	1.24	0.08	441.62	125.88	184.48	1,297.05	**634.62**
**2,7-PFP**	1.29	0.08	441.78	127.51	1,564.86	1,297.39	**2,016.72**

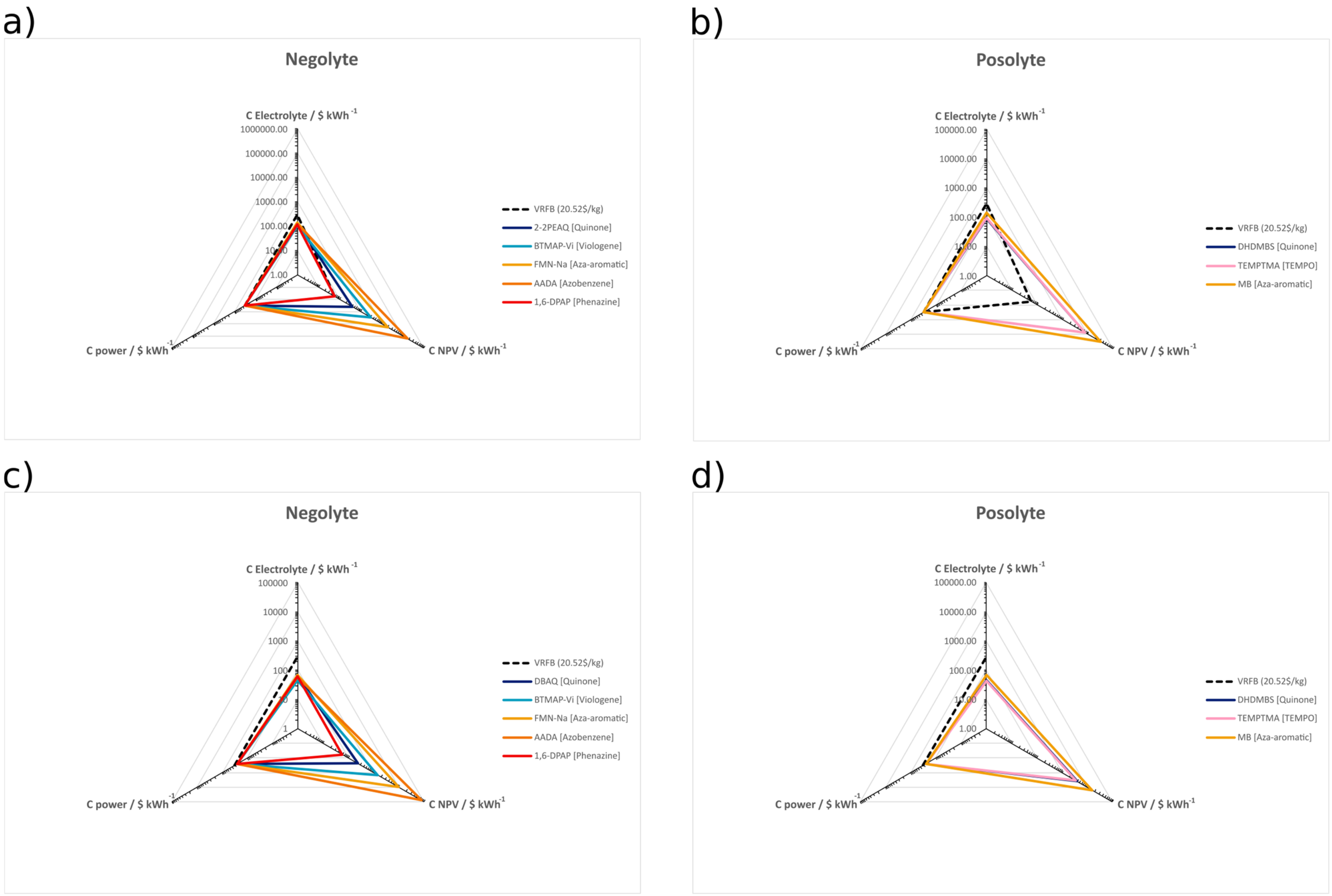

Figure S1: ***Spiderweb plot of active materials with lowest capital cost value for each molecular type assuming WP1 (spec. power 0.1 W cm^{-2}).*** *a), b) Present Case; c), d) Future Case.*

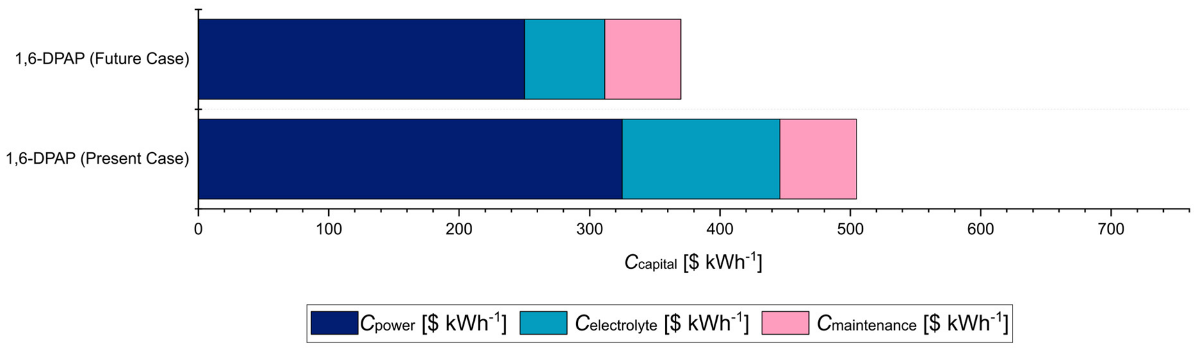

Figure S2: ***Side-by-side comparison of the different scenarios on the individual cost contributions.*** *Comparison of the individual cost contributions for the two different discussed scenarios "Present Case" and "Future Case" of 1,6-DPAP. (WP1 – spec. power 0.1 W cm^{-2})*

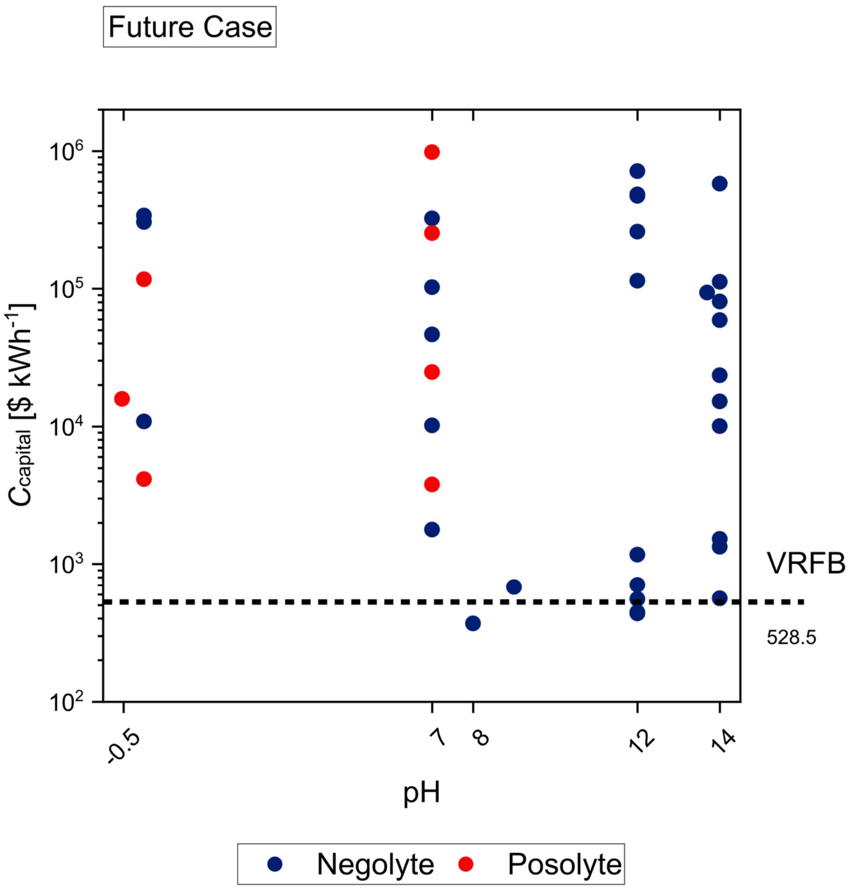

Figure S3: ***Capital costs in relation to the pH value of each electrolyte.*** *Future Case capital costs $C_{capital}$ as a function of the electrolyte pH value at which the active materials have been electrochemically cycled for negolyte and posolyte active materials (WP1: spec. power 0.1 W cm^{-2}) (source data are provided as a Source Data file).*

Table S12: Cost results for the inorganic posolyte material ammonium ferrocyanide $(NH_4)_4[FeCN_6]$ calculated for both considered working points: fixed specific power and fixed voltage efficiency, respectively.

Working Point	$U_{d,\,cell}$ / V	I_d / A cm^{-2}	n_{stack}	$C_{electrolyte}$ / \$ kWh^{-1}	$C_{maintenance}$ / \$ kWh^{-1}	C_{power} / \$ kW^{-1}	$C_{Capital}$ / \$ kWh^{-1}
specific power (0.1 W cm^{-2})	1.33	0.076	441.34	43.83	283.71	1,296.49	**651.66**
voltage efficiency (0.916)	1.29	0.114	117.31	45.20	278.98	1,006.63	**575.84**

Working point 2 - discharge voltage efficiency 0.916

Table S13: Calculation results: Future Case – $C_{a,\ org.}$ = 3.48 $/kg, size selective membrane, vanadium residual value. (WP2 –discharge voltage efficiency 0.916)

Active material	$U_{d,\ cell}$ / V	I_d / A cm^{-2}	n_{stack}	$C_{electrolyte}$ / $ kWh^{-1}	$C_{maintenance}$ / $ kWh^{-1}	C_{power} / $ kW^{-1}	$C_{Capital}$ / $ kWh^{-1}
**Inorganic salts:**							
**VRFB (20.52 $ kg^{-1})**	1.17	0.00054	70,227.39	185.97	5,832.54	145,824.78	**42,474.70**
**VRFB (63.49 $ kg^{-1})**	1.17	0.00054	70,227.39	528.27	5,635.87	145,824.78	**42,620.34**
**VRFB (5.51 $ kg^{-1})**	1.17	0.00054	70,227.39	66.40	5,901.22	145,824.78	**42,423.81**
**Quinones:**							
**BQDS**	1.35	0.21	155.19	45.49	123,632.13	600.32	**123,827.7**
**DHDMBS**	1.33	0.17	195.36	47.15	4,016.49	656.71	**4,227.82**
**DHBQ**	1.38	0.29	110.68	37.03	99,968.6	537.84	**100,140.09**
**2,7-AQDS**	1.27	0.26	136.43	69.18	11,240.04	573.99	**11,452.72**
**ARS**	1.41	0.25	128.46	67.27	324,453.65	562.8	**324,661.63**
**AQS**	1.31	0.18	186.1	62.17	356,033.65	643.72	**356,256.75**
**DHAQ**	1.33	0.26	130.07	70.59	119,129.77	565.06	**119,341.63**
**DBEAQ**	1.33	0.26	127.19	68.19	1,056.75	561.01	**1,265.19**
**DPivOHAQ**	1.25	0.22	159.8	79	375	606.79	**605.7**

Active material	$U_{d, cell}$ / V	I_d / A cm^{-2}	n_{stack}	$C_{electrolyte}$ / \$ kWh^{-1}	$C_{maintenance}$ / \$ kWh^{-1}	C_{power} / \$ kW^{-1}	$C_{Capital}$ / \$ kWh^{-1}
**DBAQ**	1.25	0.24	151.26	70.92	222.31	594.8	**441.93**
**2,3-HCNQ**	1.18	0.23	165.16	60.61	62,151.07	614.32	**62,365.26**
**2,6-DPPEAQ**	1.41	0.24	129.57	74.86	353.7	564.35	**569.65**
**TMHQ**	1.74	0.36	70.89	50.24	1,059,970.87	481.98	**1,060,141.61**
**2-2PEAQ**	1.14	0.2	189.76	72.37	230.7	648.84	**465.28**
**Viologens:**							
**MVi**	1.5	0.16	190.51	57.09	339,033.65	649.9	**339,253.22**
**BTMAP-Vi**	1.75	0.37	68.45	50.01	1,563.28	478.55	**1,732.93**
**MTMAP-Vi**	1.8	0.38	64.03	45.34	110,178.64	472.35	**110,342.07**
**BTMAP-TTZ-Vi**	1.49	0.31	95.38	73.57	49,674.88	516.36	**49,877.54**
**$(SPr)_2$-Vi**	1.48	0.3	100.99	78.13	10,558.35	524.24	**10,767.53**
**TEMPO:**							
**4-OH-TEMPO**	1.66	0.15	181.76	44.57	264,692.86	637.61	**264,896.83**
**TEMPOSP**	1.68	0.18	145.21	62.7	26,135.96	586.3	**26,345.23**
**TEMPTMA**	1.84	0.35	67.8	45.29	3,737.11	477.64	**3,901.81**
**Aza-aromatics:**							

Active material	$U_{d,\,cell}$ / V	I_d / A cm^{-2}	n_{stack}	$C_{electrolyte}$ / \$ kWh^{-1}	$C_{maintenance}$ / \$ kWh^{-1}	C_{power} / \$ kW^{-1}	$C_{Capital}$ / \$ kWh^{-1}
**ACA**	1.27	0.04	777.07	62.35	22,648.42	1,473.33	**23,079.11**
**FMN-Na**	1.19	0.24	155.25	73	10,293.33	600.4	**10,516.43**
**MB**	1.04	0.22	192.2	71.18	16,330.03	652.27	**16,564.28**
**Azobenzene:**							
**AADA**	1.24	0.2	182.1	62.76	84,720.35	638.1	**84,942.63**
**Phenazine:**							
**HP**	1.32	0.26	130.17	48.28	615,782.83	565.2	**615,972.42**
**AHP**	1.42	0.22	142.68	75.79	15,816.31	582.76	**16,037.79**
**BHPC**	1.42	0.28	112.62	51.74	1,278.23	540.56	**1,465.11**
**2,7-DGAP**	1.29	0.21	160.89	67.15	500,153.92	608.31	**500,373.15**
**2,7-DAAP**	1.31	0.2	171.61	65.77	275,179.65	623.37	**275,401.27**
**2,7-DPAP**	1.29	0.22	153.95	65.9	511,483.87	598.58	**511,699.41**
**2,7-DBAP**	1.3	0.23	151.21	68.26	760,167.82	594.73	**760,384.75**
**1,8-DGAP**	1.34	0.18	184.56	64.03	120,492.35	641.54	**120,716.77**
**1,6-DPAP**	1.33	0.2	167.52	64.8	35.41	617.63	**254.62**
**1,6-PFP**	1.22	0.08	482.53	68.13	130.04	1,059.84	**463.13**

Active material	$U_{d,\,cell}$ / V	I_d / A cm^{-2}	n_{stack}	$C_{electrolyte}$ / \$ kWh^{-1}	$C_{maintenance}$ / \$ kWh^{-1}	C_{power} / \$ kW^{-1}	$C_{Capital}$ / \$ kWh^{-1}
**1,8-PFP**	1.25	0.14	261.54	65.52	108.84	749.62	**361.76**
**2,7-PFP**	1.27	0.19	186.78	70.94	875	644.66	**1,107.1**

Table S14: Calculation results: Present Case – $C_{a, org.}$ = 9 $/kg, Nafion membrane, no vanadium residual value. (WP2 –discharge voltage efficiency 0.916)

Active material	$U_{d, cell}$ / V	I_d / A cm^{-2}	n_{stack}	$C_{electrolyte}$ / $ kWh^{-1}	$C_{maintenance}$ / $ kWh^{-1}	C_{power} / $ kW^{-1}	$C_{Capital}$ / $ kWh^{-1}
**Inorganic salts:**							
**VRFB**	1.17	0.00054	70,227.39	185.97	5,926.58	145,824.78	**42,568.75**
**Quinones:**							
**BQDS**	1.35	0.1	315.72	94.87	257,800.69	1,036.32	**258,154.65**
**DHDMBS**	1.33	0.09	382.28	91.57	7,780.62	1,174.16	**8,165.73**
**DHBQ**	1.38	0.13	254.42	71.83	193,886	909.36	**194,185.17**
**2,7-AQDS**	1.27	0.11	308.62	132.36	21,491.09	1,021.62	**21,878.86**
**ARS**	1.41	0.12	267.27	116.29	560,833.17	935.97	**561,183.45**
**AQS**	1.31	0.09	375.63	114.77	657,309.52	1,160.4	**657,714.4**
**DHAQ**	1.33	0.12	285.83	117.23	197,827.3	974.43	**198,188.14**
**DBEAQ**	1.33	0.12	283.66	133.04	2,044.45	969.92	**2,419.96**
**DPivOHAQ**	1.25	0.1	336.73	147.46	684.83	1,079.83	**1,102.24**
**DBAQ**	1.25	0.11	331.68	136.68	412.17	1,069.38	**816.19**
**2,3-HCNQ**	1.18	0.1	366	110.52	113,322.07	1,140.46	**113,717.7**

Active material	$U_{d, cell}$ / V	I_d / A cm^{-2}	n_{stack}	$C_{electrolyte}$ / \$ kWh^{-1}	$C_{maintenance}$ / \$ kWh^{-1}	C_{power} / \$ kW^{-1}	$C_{Capital}$ / \$ kWh^{-1}
**2,6-DPPEAQ**	1.41	0.12	267.6	143.49	660.14	936.67	**1,037.79**
**TMHQ**	1.74	0.16	161.68	107.54	2,268,796.96	717.31	**2,269,083.82**
**2-2PEAQ**	1.14	0.1	402.97	133.66	412.57	1,217.02	**850.49**
**Viologens:**							
**MVi**	1.49	0.09	326.01	124.65	740,213.76	1,057.64	**740,602.82**
**BTMAP-Vi**	1.75	0.16	160.19	106.66	3,310.89	714.22	**3,596.1**
**MTMAP-Vi**	1.8	0.16	149.83	90.74	220,490.3	692.76	**220,754.24**
**BTMAP-TTZ-Vi**	1.49	0.14	220.19	153.32	103,498.37	838.47	**103,861.31**
**$(SPr)_2$-Vi**	1.48	0.13	227.72	173.62	23,436.73	854.08	**23,823.87**
**TEMPO:**							
**4-OH-TEMPO**	1.66	0.09	299.71	91.05	540,702.24	1,003.17	**541,044.08**
**TEMPOSP**	1.68	0.11	240.64	125.63	52,346.5	880.83	**52,692.34**
**TEMPTMA**	1.84	0.16	149.95	98.82	8,128.64	693.02	**8,400.72**
**Aza-aromatics:**							
**ACA**	1.27	0.03	1,165.49	113.16	41,065.82	2,796.22	**41,878.04**
**FMN-Na**	1.19	0.11	351.45	148.33	20,896.88	1,110.31	**21,322.79**

Active material	$U_{d, cell}$ / V	I_d / A cm^{-2}	n_{stack}	$C_{electrolyte}$ / \$ kWh^{-1}	$C_{maintenance}$ / \$ kWh^{-1}	C_{power} / \$ kW^{-1}	$C_{Capital}$ / \$ kWh^{-1}
**MB**	1.04	0.1	445.74	141.71	32,495.72	1,305.6	**32,963.84**
**Azobenzene:**							
**AADA**	1.24	0.1	371.65	128.81	173,861.61	1,152.15	**174,278.45**
**Phenazine:**							
**HP**	1.32	0.12	290.88	90.69	1,156,527.41	984.87	**1,156,864.31**
**AHP**	1.42	0.11	283.71	116.65	24,336.46	970.02	**24,695.61**
**BHPC**	1.42	0.12	252.73	100.43	2,463.97	905.87	**2,790.87**
**2,7-DGAP**	1.29	0.1	331.51	124.74	929,052.23	1,069.03	**929,444.24**
**2,7-DAAP**	1.31	0.1	334.57	125.53	525,181.5	1,075.35	**525,575.87**
**2,7-DPAP**	1.29	0.11	325.7	126.5	981,841.97	1,056.99	**982,232.71**
**2,7-DBAP**	1.3	0.11	316.08	131.47	1,464,229.38	1,037.06	**1,464,620.12**
**1,8-DGAP**	1.34	0.1	343.95	119.62	225,075.28	1,094.79	**225,468.59**
**1,6-DPAP**	1.33	0.1	336.71	123.75	49.78	1,079.78	**443.47**
**1,6-PFP**	1.22	0.05	807.16	128.87	218.2	2,054.1	**860.59**
**1,8-PFP**	1.24	0.08	474.81	125.03	186.42	1,365.8	**652.9**
**2,7-PFP**	1.27	0.09	373.56	129.39	1,581.27	1,156.12	**1,999.69**

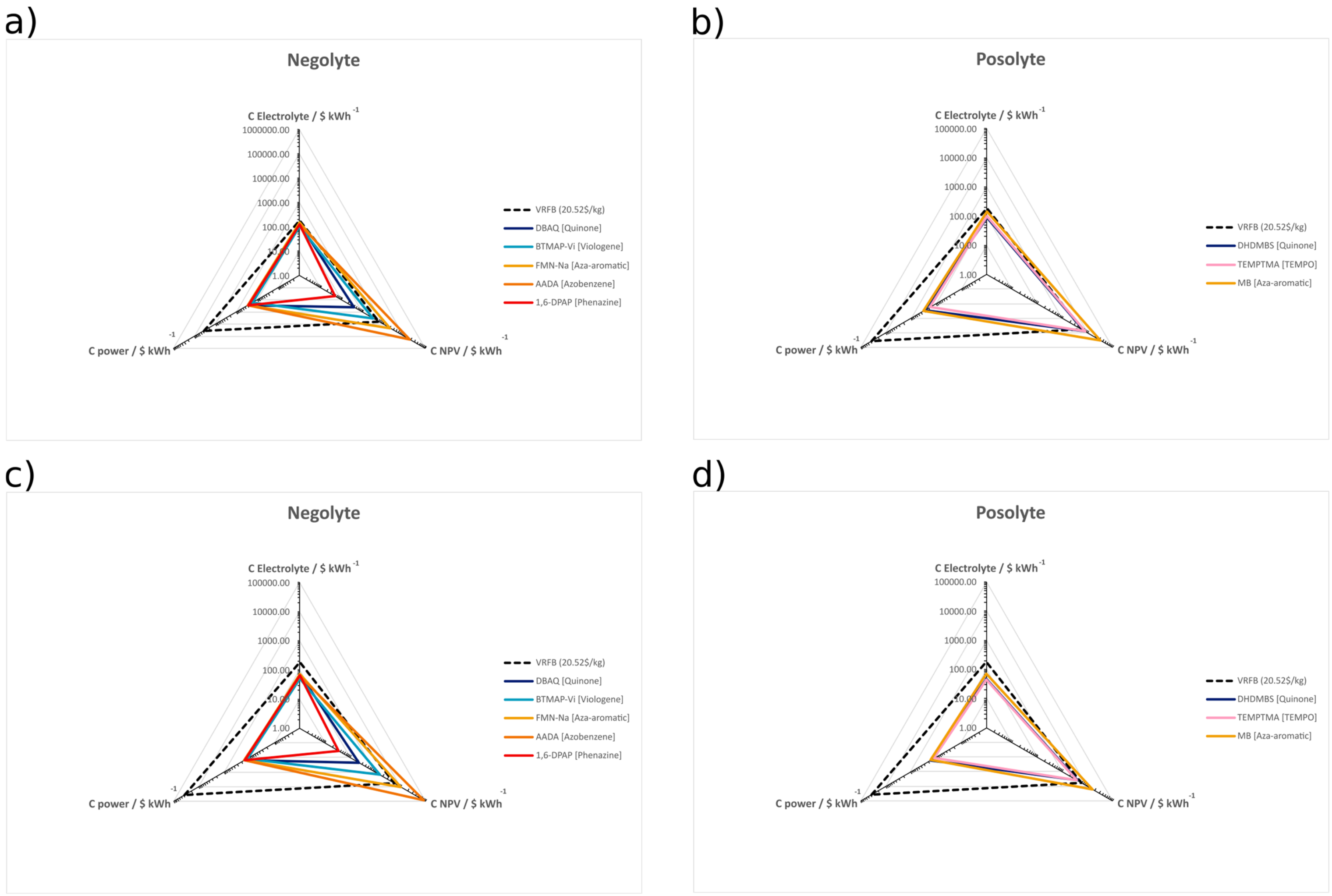

Figure S4: ***Spiderweb plot of active materials with lowest capital cost value for each molecular type assuming WP1 (discharge voltage efficiency 0.916).*** *a), b) Present Case; c), d) Future Case.*

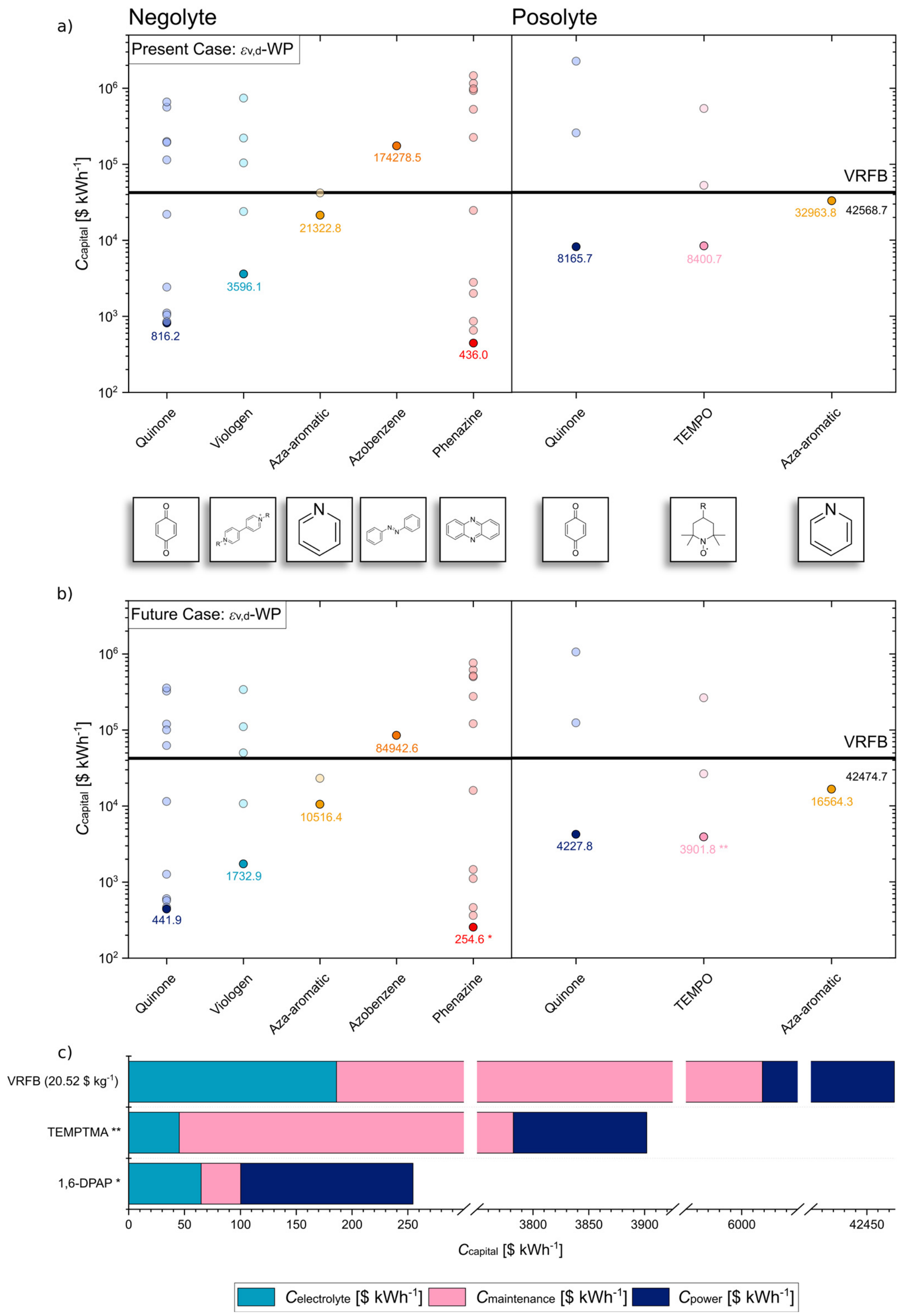

Figure S5: ***Result of the calculations performed with the RFB cost model assuming the working point WP2 (discharge voltage efficiency $\varepsilon_{v,\,d}$ of 0.916).*** *a), b) Data points as the result of the calculations performed with the RFB cost model assuming the two different scenarios: a) Present Case, b) Future Case. A distinction is made between the two scenarios discussed above, Present Case and Future Case, as well as between different classes of molecules and half-cell sides. c) Bar plot that shows the total cost broken down into the three main contributions for the most economical active materials per group (negolyte, posolyte and VRFB) based on the Future Case results. (All data points are listed in table S13 and S14, source data are provided as a Source Data file)*

As additional working point, further calculations were done based on the fixed discharge voltage efficiency ($\varepsilon_{v,\ d}$) of 0.916. The corresponding results are depicted in Figure S5 in supporting information. With changing the working point (WP) some organic active materials yield slightly reduced $C_{capital}$ in comparison to the fixed specific power 0.1 W cm^{-2} shown in Figure 1 a) and b). The phenazine benefits most from the change to the fixed discharge voltage efficiency with 254.6 \$ kWh^{-1}. On the posolyte side a slight increase of $C_{capital}$ is visual. The VRFB reveals a significant increase of $C_{capital}$ for either, Present Case as well as Future Case scenario (e. g., Future Case - WP1: 503.2 \$ kWh^{-1} vs WP2: 42458.7 \$ kWh^{-1} with assuming a vanadium price of 20.52 \$ kg^{-1}). Comparing Figure S5 c) with Figure 1 c) reveals no significant changes for the depicted organic active materials for all cost contributions. However, the VRFB shows major increase in C_{power} and $C_{maintenance}$ when applying the discharge voltage efficiency.

In equation (S6) the necessary electrode surface area *A* is calculated in dependency of the applied current density i_d and discharge cell voltage U_d. While using a fixed specific power in WP1 the surface area is independent of the individual parameters as their product is predefined. In WP2 (fixed discharged voltage efficiency) the calculated polarization curve of the individual active materials is necessary to determine i_d and U_d. Figure S6 shows the polarization curve as well as power curve for VRFB, highlighted are i_d and U_d used for the two WPs discussed before. The plot shows with assuming the discharge voltage efficiency WP the VRFB is operating at the beginning of the activation region at a very low specific power of $6.31 \cdot 10^{-4}$ W cm^{-2}. This leads to a significant increase of necessary cell stacks (443 stacks with WP1 vs 70227 stacks for WP2) to reach the target power for the assumed RFB system increasing C_{power} as well as the stack replacement costs in $C_{maintenance}$. The considered organic active materials are less influenced by this working point change because of a better kinetic of the electrochemical conversion (1,6-DPAP: $5.13 \cdot 10^{-4}$ cm s^{-1} [57] vs. VO^{2+}: $3.0 \cdot 10^{-7}$ cm s^{-1} [23]) and therefore a less prominent activation region (see Figure S6 c) and d)).

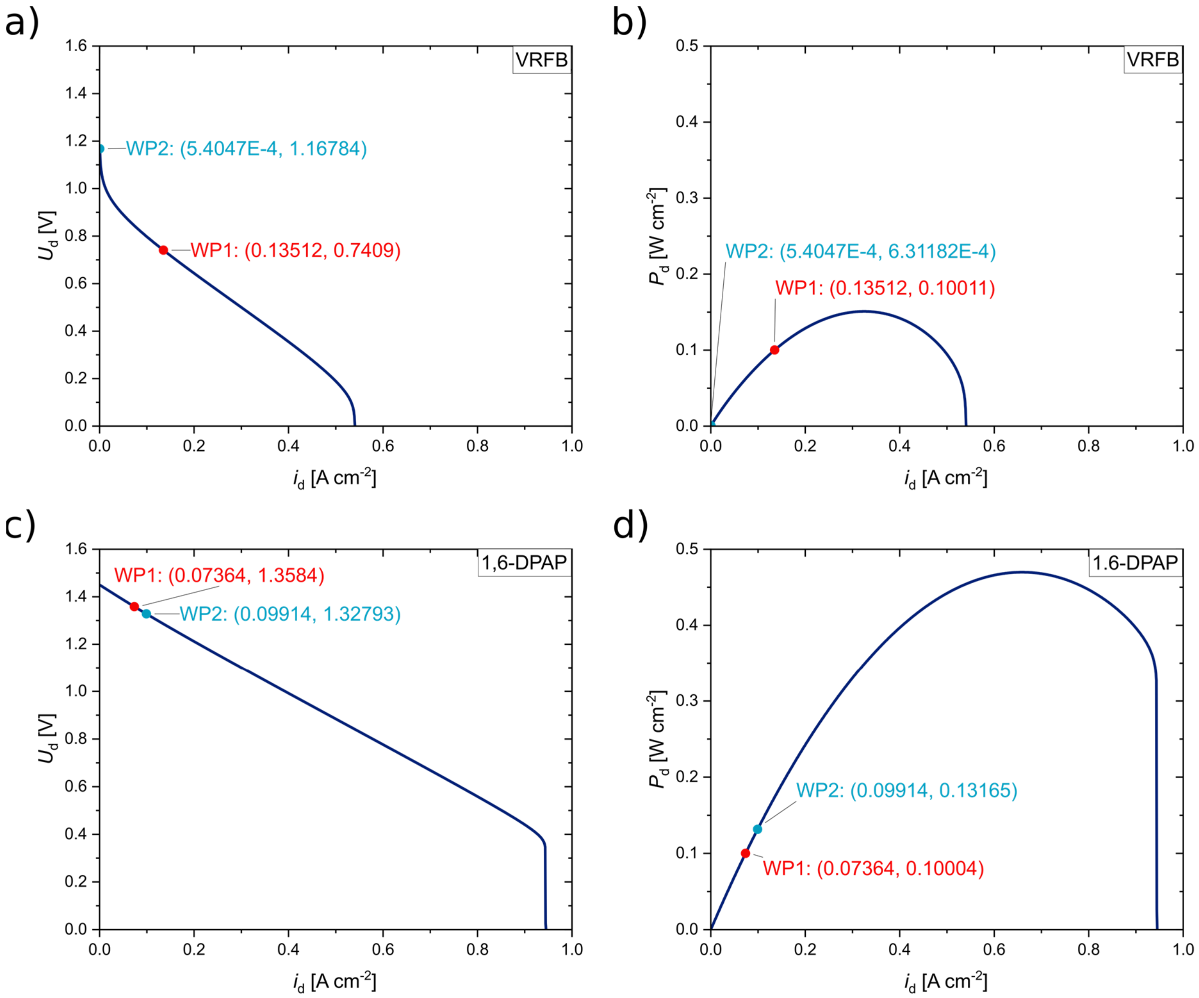

Figure S6: ***Side-by-side comparison of the polarization curves for VRFB and 1,6-DPAP.*** *Assumed polarization curves of a) VRFB and c) 1,6-DPAP as well as specific power plotted over discharge current of b) VRFB and d) 1,6-DPAP. The two in this publication discussed working points (WP1: fixed specific power of 0.1 W cm^{-2}; WP2: fixed discharge voltage efficiency $\varepsilon_{V,\,d}$ of 0.916) are highlighted.*

4. Supplementary Note: Sensitivity analysis

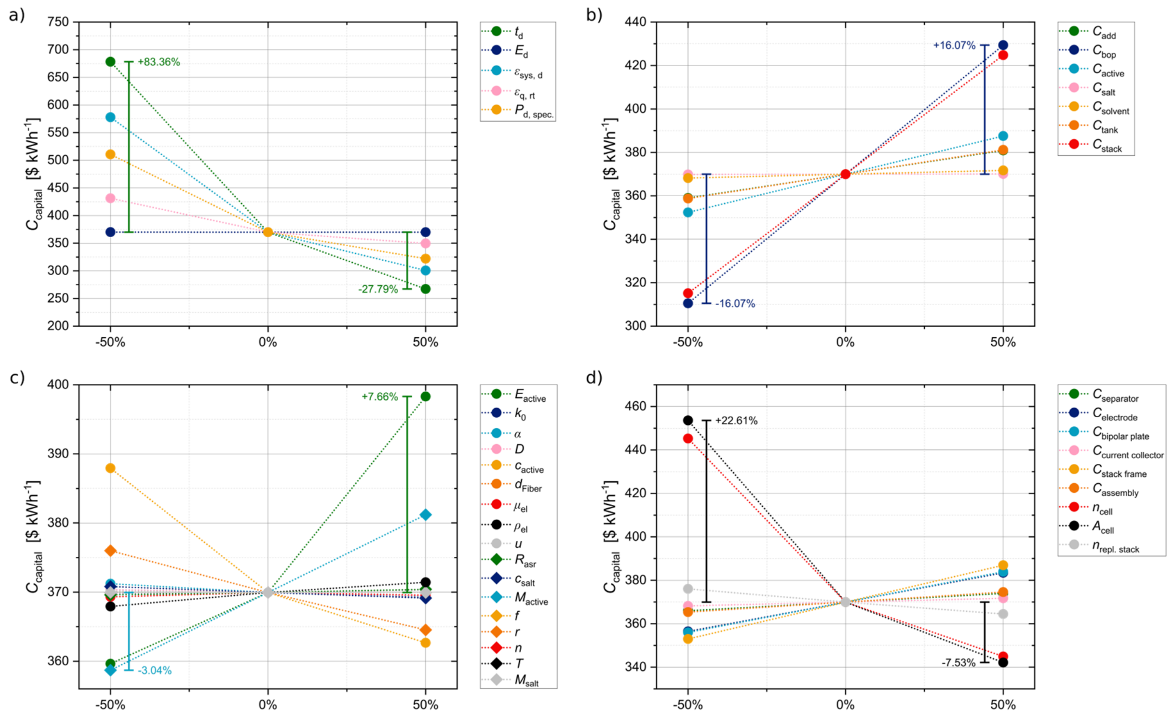

*Figure S7: **Results of local sensitivity analysis with input values of 1,6-DPAP with fixed specific power of 0.1 W cm^{-2} and Future Case assumed used as interest point.** Parameters are separated in different categories: a) input parameters that determine the RFB system, b) all costs that are involved in the calculation model, c) input parameters determined by the chemistry of the active material and electrolyte, d) stack relevant input parameters (source data are provided as a Source Data file).*

Figure S7 shows a local sensitivity analysis with the input for 1,6-DPAP at WP1 (fixed specific power) as reference point. The input parameters are varied by ±50% and categorized: Fig. S7 a) input parameters that determine the RFB system, b) all costs that are involved in the calculation model, c) input parameters determined by the chemistry of the active material and electrolyte, d) stack relevant input parameters.

Figure S7 a) indicates that system size-relevant parameters can highly influence the outcome RFB price. Especially the discharge time shows a significant relation to the capital price of 1,6-DPAP. In Figure S7 b), the costs with the most impact on the price of the selected RFB have C_{bop} and C_{stack} while other costs like the active material cost show only slight influence. Looking into detail on the cell chemistry input parameters in Figure S7 c) major capital cost changes can be triggered by alternating the active material formal potential. Out of the input parameters of the RFB stack the number of cell n_{cell} as well as the cell area A_{cell} have the most influence on C_{capital} depicted in Figure S7 d).

The trends depicted in Figure S7 indicate that by adapting certain parameters of the best-performing organic active material 1,6-DPAP even lower C_{capital} might be achievable. Creating active materials with even lower redox potentials or higher solubility might help reaching the goal of low-cost organic RFBs

while already optimized parameters like the annual electrolyte replacement fraction *f* show less potential for further improvement. To gain a comprehensive picture of relationships in-between input variables a more in-detail analysis is necessary in form of global sensitivity analysis and investigating the output for the three main contributions (C_{power}, $C_{electrolyte}$, and $C_{maintenance}$) separately.

Table S15: List of input values and input changes used to calculate the local sensitivity analysis. The input values of 1,6-DPAP with fixed specific power of 0.1 W cm⁻² was used as interest point.

Parameters	*Input fraction*	*Input value*	*Output $C_{capital}$ / $ kWh^{-1}*	*Change / %*
timeDischarge	-0.5	2	678.32578	83.36%
timeDischarge	0	4	369.95053	0%
timeDischarge	0.5	6	267.15879	-27.79%
energyCapacity	-0.5	2	370.15435	0.06%
energyCapacity	0	4	369.95053	0%
energyCapacity	0.5	6	369.8826	-0.02%
costAdd	-0.5	43.75	359.01303	-2.96%
costAdd	0	87.5	369.95053	0%
costAdd	0.5	131.25	380.88803	2.96%
efficiencySysDischarge	-0.5	0.47	577.80683	56.18%
efficiencySysDischarge	0	0.94	369.95053	0%
efficiencySysDischarge	0.5	1.41	300.6651	-18.73%
efficiencyCoulombicRoundTrip	-0.5	0.485	431.32201	16.59%
efficiencyCoulombicRoundTrip	0	0.97	369.95053	0%
efficiencyCoulombicRoundTrip	0.5	1.455	349.49338	-5.53%
numberCellsPerStack	-0.5	20	445.27214	20.36%
numberCellsPerStack	0	40	369.95053	0%
numberCellsPerStack	0.5	60	344.88747	-6.77%
cellArea	-0.5	0.03	453.58554	22.61%
cellArea	0	0.06	369.95053	0%
cellArea	0.5	0.09	342.11118	-7.53%
specificPowerDischarge	-0.5	0.05	510.62298	38.02%
specificPowerDischarge	0	0.1	369.95053	0%
specificPowerDischarge	0.5	0.15	322.08893	-12.94%
actMatReversiblePotential	-0.5	-0.84	359.63214	-2.79%
actMatReversiblePotential	0	-0.56	369.95053	0%
actMatReversiblePotential	0.5	-0.28	398.30555	7.66%
actMatReactionRate	-0.5	2.565E-4	369.90202	-0.01%
actMatReactionRate	0	5.13E-4	369.95053	0%
actMatReactionRate	0.5	7.695E-4	369.21206	-0.2%
actMatTransferCoeff	-0.5	0.265	371.2181	0.34%
actMatTransferCoeff	0	0.53	369.95053	0%
actMatTransferCoeff	0.5	0.795	369.43305	-0.14%
actMatDiffusionCoeff	-0.5	2.04E-6	370.37032	0.11%
actMatDiffusionCoeff	0	4.08E-6	369.95053	0%
actMatDiffusionCoeff	0.5	6.12E-6	369.47382	-0.13%
actMatConc	-0.5	0.5025	387.93707	4.86%
actMatConc	0	1.005	369.95053	0%

actMatConc	0.5	1.5075	362.65751	-1.97%
dFiber	-0.5	5E-6	370.01995	0.02%
dFiber	0	1E-5	369.95053	0%
dFiber	0.5	1.5E-5	370.34014	0.11%
solventViscosity	-0.5	0.496	369.30976	-0.17%
solventViscosity	0	0.992	369.95053	0%
solventViscosity	0.5	1.488	369.54531	-0.11%
solventDensity	-0.5	0.5217	367.92897	-0.55%
solventDensity	0	1.0434	369.95053	0%
solventDensity	0.5	1.5651	371.42383	0.4%
flowVelocity	-0.5	0.0505	370.14563	0.05%
flowVelocity	0	0.101	369.95053	0%
flowVelocity	0.5	0.1515	370.2805	0.09%
asr	-0.5	0.21	369.61277	-0.09%
asr	0	0.42	369.95053	0%
asr	0.5	0.63	370.4067	0.12%
costBOP	-0.5	237.7829	310.50481	-16.07%
costBOP	0	475.5658	369.95053	0%
costBOP	0.5	713.3487	429.39626	16.07%
actMatCost	-0.5	1.74	352.35838	-4.76%
actMatCost	0	3.48	369.95053	0%
actMatCost	0.5	5.22	387.54269	4.76%
saltCost	-0.5	0.046	369.82576	-0.03%
saltCost	0	0.092	369.95053	0%
saltCost	0.5	0.138	370.07531	0.03%
solventCost	-0.5	0.05	368.18797	-0.48%
solventCost	0	0.1	369.95053	0%
solventCost	0.5	0.15	371.7131	0.48%
tankCost	-0.5	205.5	358.74429	-3.03%
tankCost	0	411	369.95053	0%
tankCost	0.5	616.5	381.15678	3.03%
saltConc	-0.5	0.5	370.809	0.23%
saltConc	0	1	369.95053	0%
saltConc	0.5	1.5	369.13555	-0.22%
actMatMolMass	-0.5	177.185	358.7051	-3.04%
actMatMolMass	0	354.37	369.95053	0%
actMatMolMass	0.5	531.555	381.19597	3.04%
replacementFraction	-0.5	0.00274	369.95053	0%
replacementFraction	0	0.00548	369.95053	0%
replacementFraction	0.5	0.00821	369.95053	0%
interestRate	-0.5	0.01	375.99391	1.63%
interestRate	0	0.02	369.95053	0%
interestRate	0.5	0.03	364.52504	-1.47%
operationalLifetime	-0.5	10	369.95053	0%
operationalLifetime	0	20	369.95053	0%
operationalLifetime	0.5	30	369.95053	0%
stackOperationalTime	-0.5	5	376.02549	1.64%

stackOperationalTime	0	10	369.95053	0%
stackOperationalTime	0.5	15	364.44826	-1.49%
threshold	-0.5	0.4	369.95053	0%
threshold	0	0.8	369.95053	0%
threshold	0.5	1.2	369.95053	0%
M salt	-0.5	37.27415	369.96138	0%
M salt	0	74.5483	369.95053	0%
M salt	0.5	111.82245	369.93969	-0%
separatorCost	-0.5	15	365.97863	-1.07%
separatorCost	0	30	369.95053	0%
separatorCost	0.5	45	373.92244	1.07%
electrodeCost	-0.5	25.5	356.44607	-3.65%
electrodeCost	0	51	369.95053	0%
electrodeCost	0.5	76.5	383.455	3.65%
bipolarPlateCost	-0.5	51.5	355.97275	-3.78%
bipolarPlateCost	0	103	369.95053	0%
bipolarPlateCost	0.5	154.5	383.92832	3.78%
currentCollectorCost	-0.5	8	368.18525	-0.48%
currentCollectorCost	0	16	369.95053	0%
currentCollectorCost	0.5	24	371.71582	0.48%
stackFrameCost	-0.5	154	352.95963	-4.59%
stackFrameCost	0	308	369.95053	0%
stackFrameCost	0.5	462	386.94144	4.59%
assemblyCost	-0.5	0.255	365.33651	-1.25%
assemblyCost	0	0.51	369.95053	0%
assemblyCost	0.5	0.765	374.56456	1.25%

5. Supplementary Note: Additional Figures

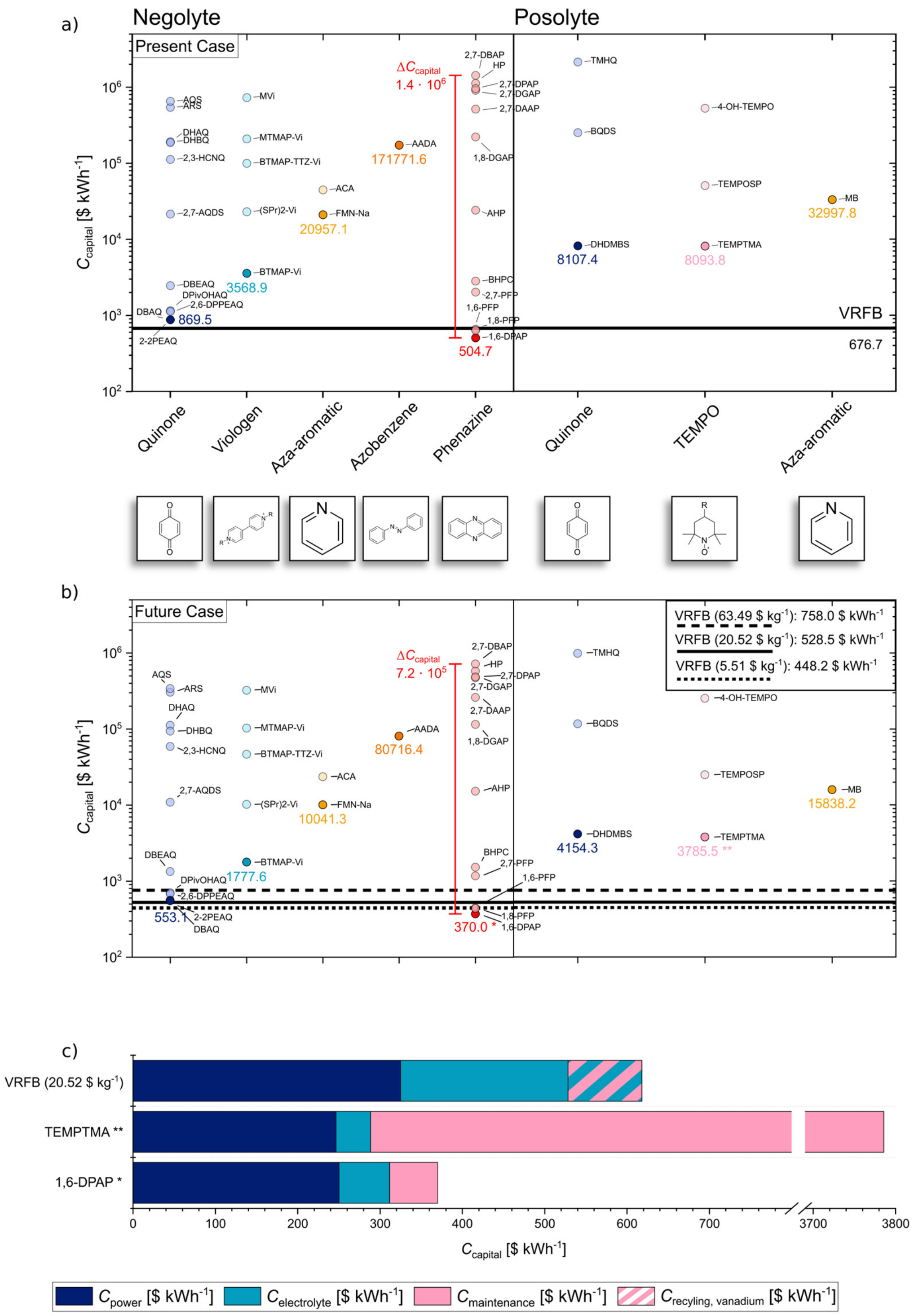

Figure S8: ***Extended version of Figure 1, each data point is assigned the abbreviation of the respective active material.*** *Main Result of the calculations performed with the RFB cost model assuming the working point WP1 (0.1 W cm^{-2} specific power). a) Data points representing the outcome of calculations assuming the Present Case conditions. b) Points depicted show data obtained by applying assumptions for the Future Case scenario. c) Bar plot that shows the total cost broken down into the three main contributions for the most economical active materials per group (negolyte, posolyte, and VRFB) based on the Future Case results (source data are provided as a Source Data file).*

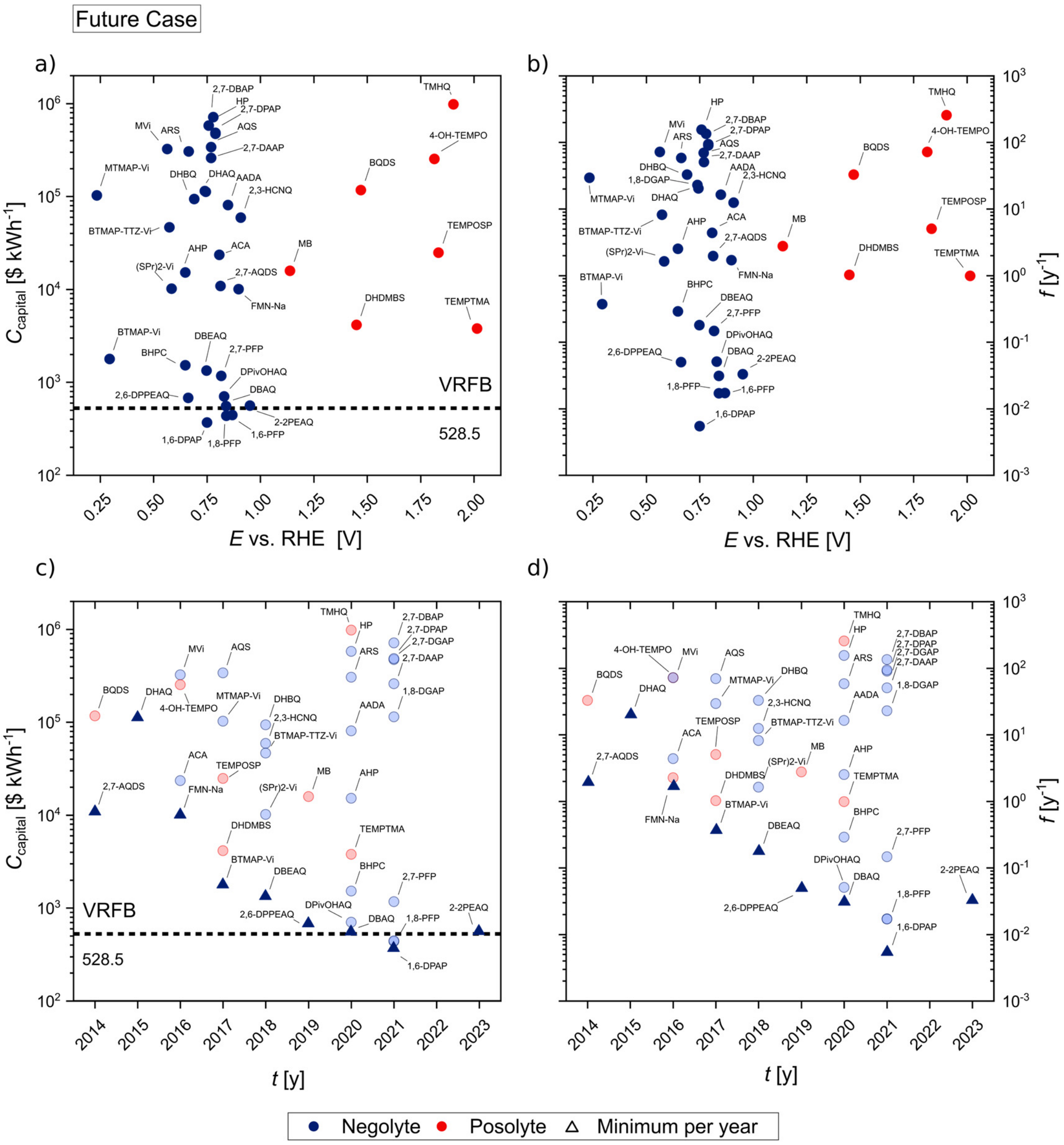

Figure S9: ***Extended version of Figure 2, each data point is assigned the abbreviation of the respective active material.*** *a) Future Case capital costs $C_{capital}$ and b) capacity fade rate f, as a function of the active material redox potential E referenced to the proton redox potential at the pH value (RHE) of the individual electrolyte for negolyte and posolyte materials. c) Overview of Future Case capital costs $C_{capital}$, and d) capacity fade rate f, in relation to each active materials individual publication year. The minimum capital cost value for each publication year is represented in dark color triangles while values above the minimum are depicted in lighter color (source data are provided as a Source Data file).*

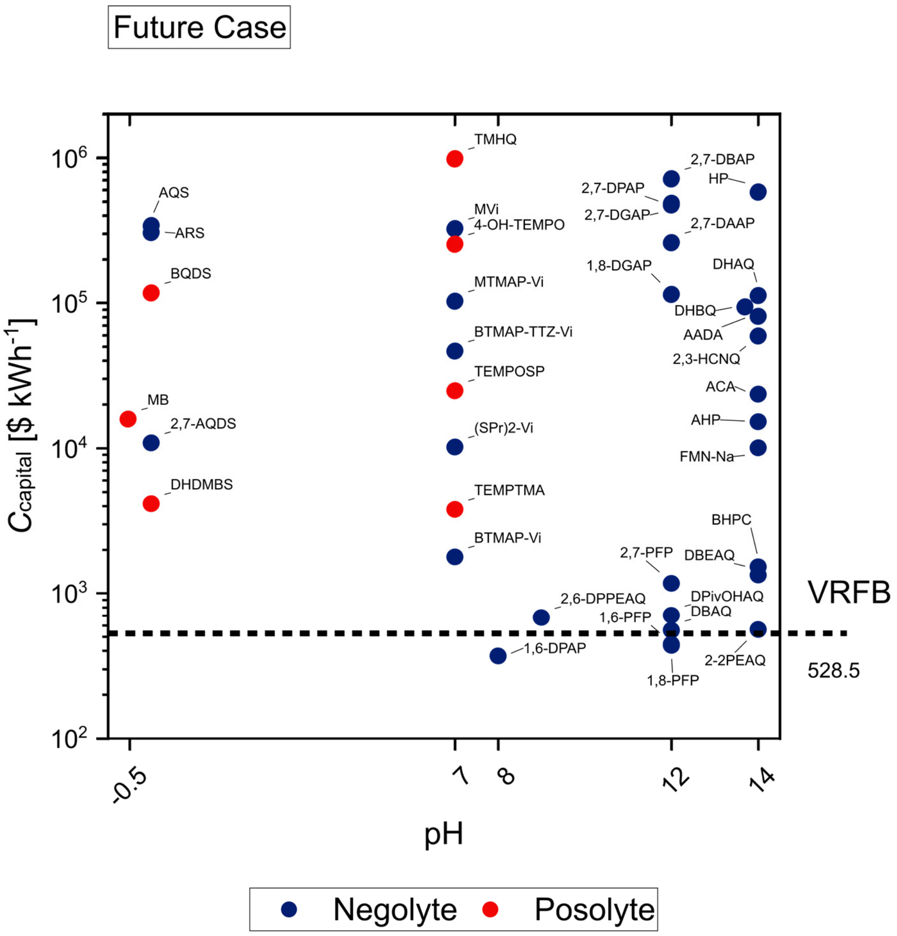

Figure S10: ***Extended version of Figure S3, each data point is assigned the abbreviation of the respective active material.*** *Capital costs in relation to the pH value of each electrolyte. Future Case capital costs $C_{capital}$ as a function of the electrolyte pH value at which the active materials have been electrochemically cycled for negolyte and posolyte active materials (WP1: spec. power 0.1 W cm^{-2}) (source data are provided as a Source Data file).*

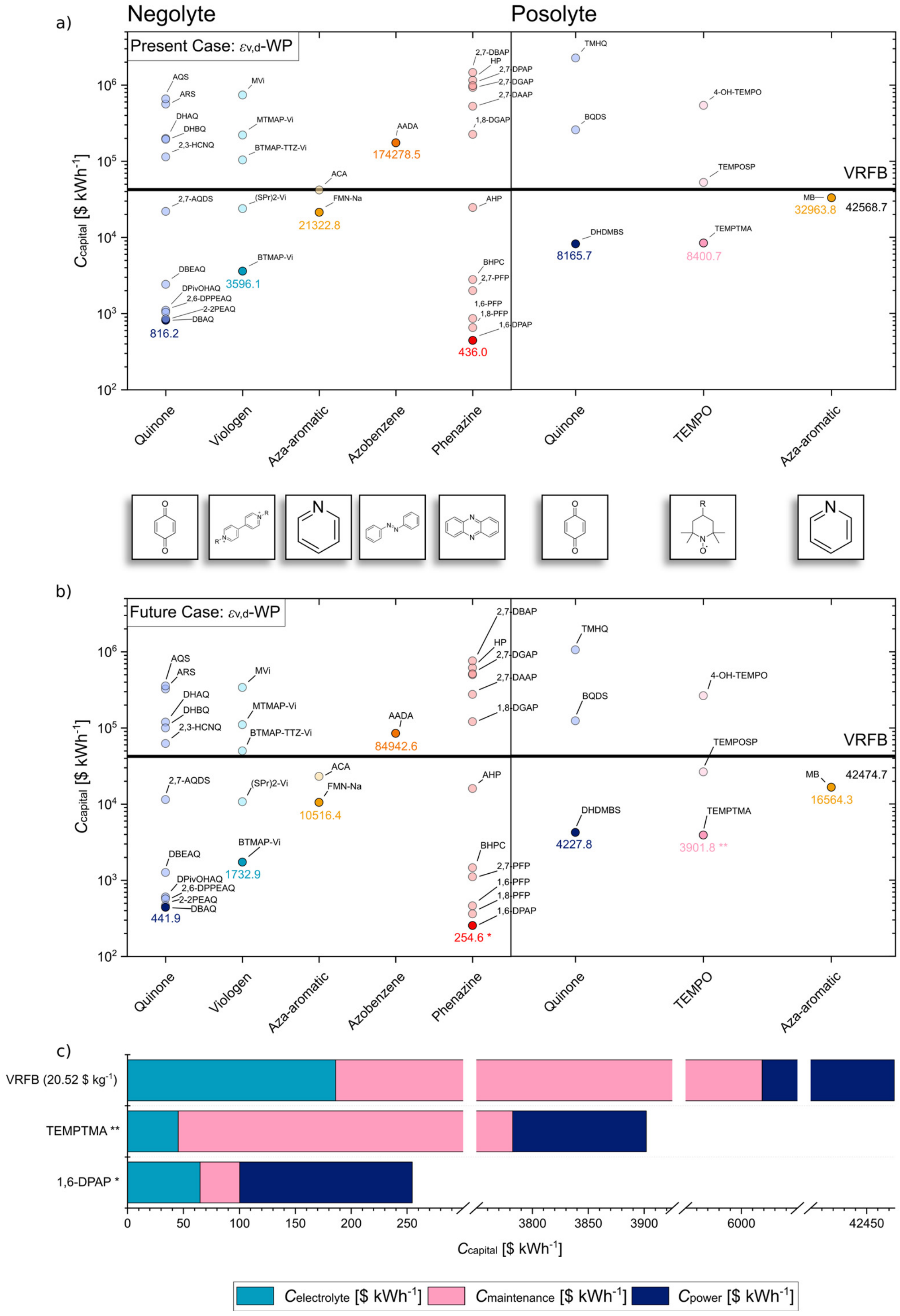

*Figure S11: **Extended version of Figure S5, each data point is assigned the abbreviation of the respective active material.** Result of the calculations performed with the RFB cost model assuming the working point WP2 (discharge voltage efficiency $\varepsilon_{v,\,d}$ of 0.916). a), b) Data points as the result of the calculations performed with the RFB cost model assuming the two different scenarios: a) Present Case, b) Future Case. A distinction is made between the two scenarios discussed above, Present Case and Future Case, as well as between different classes of molecules and half-cell sides. c) Bar plot that shows the total cost broken down into the three main contributions for the most economical active materials per group (negolyte, posolyte and VRFB) based on the Future Case results (All data points are listed in table S13 and S14, source data are provided as a Source Data file).*

6. Supplementary References

1. Darling, R. M., Gallagher, K. G., Kowalski, J. A., Ha, S. & Brushett, F. R. Pathways to low-cost electrochemical energy storage: A comparison of aqueous and nonaqueous flow batteries. *Energy Environ Sci* **7**, 3459–3477 (2014).
2. Dmello, R., Milshtein, J. D., Brushett, F. R. & Smith, K. C. Cost-driven materials selection criteria for redox flow battery electrolytes. *J Power Sources* **330**, 261–272 (2016).
3. Brushett, F. R., Aziz, M. J. & Rodby, K. E. On Lifetime and Cost of Redox-Active Organics for Aqueous Flow Batteries. *ACS Energy Lett* **5**, 879–884 (2020).
4. Minke, C., Kunz, U. & Turek, T. Techno-economic assessment of novel vanadium redox flow batteries with large-area cells. *J Power Sources* **361**, 105–114 (2017).
5. Minke, C. & Dorantes Ledesma, M. A. Impact of cell design and maintenance strategy on life cycle costs of vanadium redox flow batteries. *J Energy Storage* **21**, 571–580 (2019).
6. Milshtein, J. D., Darling, R. M., Drake, J., Perry, M. L. & Brushett, F. R. The Critical Role of Supporting Electrolyte Selection on Flow Battery Cost. *J Electrochem Soc* **164**, A3883–A3895 (2017).
7. Perry, M. L., Darling, R. M. & Zaffou, R. High Power Density Redox Flow Battery Cells. *ECS Trans* **53**, 7–16 (2013).
8. Janardhanan, V. M. & Deutschmann, O. Modeling of solid-oxide fuel cells. *Zeitschrift fur Physikalische Chemie* **221**, 443–479 (2007).
9. Hofmann, J. D. & Schröder, D. Which Parameter is Governing for Aqueous Redox Flow Batteries with Organic Active Material? *Chem Ing Tech* **91**, 786–794 (2019).
10. Esan, O. C. *et al.* Modeling and Simulation of Flow Batteries. *Adv Energy Mater* **10**, 1–42 (2020).
11. Bard, A. J. & Faulkner, L. R. *Electrochemical methods : fundamentals and applications*. (Wiley, 2001).
12. Hofmann, J. D. *et al.* Tailoring Dihydroxyphthalazines to Enable Their Stable and Efficient Use in the Catholyte of Aqueous Redox Flow Batteries. *Chemistry of Materials* **32**, 3427–3438 (2020).
13. Hamann, C. H. & Vielstich, Wolf. *Elektrochemie*. (Wiley-VCH, 2005).

14. Watt-Smith, M. J., Ridley, P., Wills, R. G. A., Shah, A. A. & Walsh, F. C. The importance of key operational variables and electrolyte monitoring to the performance of an all vanadium redox flow battery. *Journal of Chemical Technology and Biotechnology* **88**, 126–138 (2013).

15. M. Becker. Validation of a two-dimensional model for vanadium redox-flow batteries. (2020).

16. Gregory, T. D., Perry, M. L. & Albertus, P. Cost and price projections of synthetic active materials for redox flow batteries. *J Power Sources* **499**, 229965 (2021).

17. Martin, J., Schafner, K. & Turek, T. Preparation of Electrolyte for Vanadium Redox-Flow Batteries Based on Vanadium Pentoxide. *Energy Technology* **8**, 2000522 (2020).

18. LIVE Vanadium Price, News and Articles. https://www.vanadiumprice.com/ (2022).

19. Vanadium-Electrolyte-Solution-1.6-M-2012-114_V8.pdf. https://www.gfe.com/02_produkte_loesungen/03_vanadium-chemikalien/PDB/Vanadium-Electrolyte-Solution-1.6-M-2012-114_V8.pdf.

20. Roznyatovskaya, N. *et al.* Detection of capacity imbalance in vanadium electrolyte and its electrochemical regeneration for all-vanadium redox-flow batteries. *J Power Sources* **302**, 79–83 (2016).

21. Naumann, M., Karl, R. C., Truong, C. N., Jossen, A. & Hesse, H. C. Lithium-ion battery cost analysis in PV-household application. *Energy Procedia* **73**, 37–47 (2015).

22. Noack, J., Roznyatovskaya, N., Herr, T. & Fischer, P. The Chemistry of Redox-Flow Batteries. *Angewandte Chemie - International Edition* **54**, 9776–9809 (2015).

23. Weber, A. Z. *et al.* Redox flow batteries: A review. *J Appl Electrochem* **41**, 1137–1164 (2011).

24. Yamamura, T., Watanabe, N., Yano, T. & Shiokawa, Y. Electron-Transfer Kinetics of Np 3 + / Np 4 + , NpO + 2 at Carbon Electrodes. *J Electrochem Soc* **152**, A830 (2005).

25. Gattrell, M., Qian, J., Stewart, C., Graham, P. & MacDougall, B. The electrochemical reduction of VO2+ in acidic solution at high overpotentials. *Electrochim Acta* **51**, 395–407 (2005).

26. Luo, J. *et al.* Unprecedented Capacity and Stability of Ammonium Ferrocyanide Catholyte in pH Neutral Aqueous Redox Flow Batteries. *Joule* **3**, 149–163 (2019).

27. Yang, B. *et al.* High-Performance Aqueous Organic Flow Battery with Quinone-Based Redox Couples at Both Electrodes. *J Electrochem Soc* **163**, A1442–A1449 (2016).

28. Yang, B., Hoober-Burkhardt, L., Wang, F., Surya Prakash, G. K. & Narayanan, S. R. An Inexpensive Aqueous Flow Battery for Large-Scale Electrical Energy Storage Based on Water-Soluble Organic Redox Couples. *J Electrochem Soc* **161**, A1371–A1380 (2014).

29. Hoober-Burkhardt, L. *et al.* A New Michael-Reaction-Resistant Benzoquinone for Aqueous Organic Redox Flow Batteries. *J Electrochem Soc* **164**, A600–A607 (2017).

30. Murali, A. *et al.* Understanding and Mitigating Capacity Fade in Aqueous Organic Redox Flow Batteries. *J Electrochem Soc* **165**, A1193–A1203 (2018).

31. Yang, Z. *et al.* Alkaline Benzoquinone Aqueous Flow Battery for Large-Scale Storage of Electrical Energy. *Adv Energy Mater* **8**, 1702056 (2018).

32. Gerhardt, M. R. *et al.* Anthraquinone Derivatives in Aqueous Flow Batteries. *Adv Energy Mater* **7**, 1601488 (2017).

33. Huskinson, B. *et al.* A metal-free organic-inorganic aqueous flow battery. *Nature* **505**, 195–198 (2014).

34. Huskinson, B., Marshak, M. P., Gerhardt, M. R. & Aziz, M. J. Cycling of a Quinone-Bromide Flow Battery for Large-Scale Electrochemical Energy Storage. *ECS Trans* **61**, 27–30 (2014).

35. Zhang, S., Li, X. & Chu, D. An Organic Electroactive Material for Flow Batteries. *Electrochim Acta* **190**, 737–743 (2016).

36. Kwabi, D. G., Ji, Y. & Aziz, M. J. Electrolyte Lifetime in Aqueous Organic Redox Flow Batteries: A Critical Review. *Chem Rev* **120**, 6467–6489 (2020).

37. Lin, K. *et al.* Alkaline quinone flow battery. *Science (1979)* **349**, 1529–1532 (2015).

38. Kwabi, D. G. *et al.* Alkaline Quinone Flow Battery with Long Lifetime at pH 12. *Joule* **2**, 1894–1906 (2018).

39. Wu, M. *et al.* Extremely Stable Anthraquinone Negolytes Synthesized from Common Precursors. *Chem* **6**, 1432–1442 (2020).

40. Wang, C. *et al.* High-Performance Alkaline Organic Redox Flow Batteries Based on 2-Hydroxy-3-carboxy-1,4-naphthoquinone. *ACS Energy Lett* **3**, 2404–2409 (2018).

41. Ji, Y. *et al.* A Phosphonate-Functionalized Quinone Redox Flow Battery at Near-Neutral pH with Record Capacity Retention Rate. *Adv Energy Mater* **9**, 1–7 (2019).

42. Drazevic, E. *et al.* Investigation of Tetramorpholinohydroquinone as a Potential Catholyte in a Flow Battery. *ACS Appl Energy Mater* **2**, 4745–4754 (2019).

43. Amini, K. *et al.* An Extremely Stable, Highly Soluble Monosubstituted Anthraquinone for Aqueous Redox Flow Batteries. *Adv Funct Mater* **33**, 2211338 (2023).

44. Liu, T., Wei, X., Nie, Z., Sprenkle, V. & Wang, W. A Total Organic Aqueous Redox Flow Battery Employing a Low Cost and Sustainable Methyl Viologen Anolyte and 4-HO-TEMPO Catholyte. *Adv Energy Mater* **6**, 1501449 (2016).

45. DeBruler, C. *et al.* Designer Two-Electron Storage Viologen Anolyte Materials for Neutral Aqueous Organic Redox Flow Batteries. *Chem* **3**, 961–978 (2017).

46. Beh, E. S. *et al.* A neutral pH aqueous organic- organometallic redox flow battery with extremely high capacity retention. *ACS Energy Lett* **2**, 639–644 (2017).

47. Luo, J., Hu, B., Debruler, C. & Liu, T. L. A π-Conjugation Extended Viologen as a Two-Electron Storage Anolyte for Total Organic Aqueous Redox Flow Batteries. *Angewandte Chemie - International Edition* **57**, 231–235 (2018).

48. Debruler, C., Hu, B., Moss, J., Luo, J. & Liu, T. L. A Sulfonate-Functionalized Viologen Enabling Neutral Cation Exchange, Aqueous Organic Redox Flow Batteries toward Renewable Energy Storage. *ACS Energy Lett* **3**, 663–668 (2018).

49. Winsberg, J. *et al.* Aqueous 2,2,6,6-Tetramethylpiperidine-N-oxyl Catholytes for a High-Capacity and High Current Density Oxygen-Insensitive Hybrid-Flow Battery. *ACS Energy Lett* **2**, 411–416 (2017).

50. Janoschka, T., Martin, N., Hager, M. D. & Schubert, U. S. An Aqueous Redox-Flow Battery with High Capacity and Power: The TEMPTMA/MV System. *Angewandte Chemie - International Edition* **55**, 14427–14430 (2016).

51. Lin, K. *et al.* A redox-flow battery with an alloxazine-based organic electrolyte. *Nat Energy* **1**, 1–13 (2016).

52. Lee, W., Kwon, B. W. & Kwon, Y. Effect of Carboxylic Acid-Doped Carbon Nanotube Catalyst on the Performance of Aqueous Organic Redox Flow Battery Using the Modified Alloxazine and Ferrocyanide Redox Couple. *ACS Appl Mater Interfaces* **10**, 36882–36891 (2018).

53. Orita, A., Verde, M. G., Sakai, M. & Meng, Y. S. A biomimetic redox flow battery based on flavin mononucleotide. *Nat Commun* **7**, 1–8 (2016).

54. Zhang, C. *et al.* Phenothiazine-Based Organic Catholyte for High-Capacity and Long-Life Aqueous Redox Flow Batteries. *Advanced Materials* **31**, 1901052 (2019).

55. Zu, X., Zhang, L., Qian, Y., Zhang, C. & Yu, G. Molecular Engineering of Azobenzene-Based Anolytes Towards High-Capacity Aqueous Redox Flow Batteries. *Angewandte Chemie* **132**, 22347–22354 (2020).

56. Wang, C. *et al.* Molecular Design of Fused-Ring Phenazine Derivatives for Long-Cycling Alkaline Redox Flow Batteries. *ACS Energy Lett* **5**, 411–417 (2020).

57. Pang, S., Wang, X., Wang, P. & Ji, Y. Biomimetic Amino Acid Functionalized Phenazine Flow Batteries with Long Lifetime at Near-Neutral pH. *Angewandte Chemie - International Edition* **60**, 5289–5298 (2021).

58. Xu, J., Pang, S., Wang, X., Wang, P. & Ji, Y. Ultrastable aqueous phenazine flow batteries with high capacity operated at elevated temperatures. *Joule* **5**, 2437–2449 (2021).
